# Botulinum toxin effects on biochemical biomarkers related to inflammation-associated head and neck chronic conditions: a systematic review of clinical research

**DOI:** 10.1007/s00702-024-02869-w

**Published:** 2025-03-04

**Authors:** Ines Novo Pereira, Sara Durão, Haidar Hassan, Ana Cristina Braga, André Mariz Almeida, Ana Cristina Manso, Ricardo Faria-Almeida, Giancarlo De la Torre Canales

**Affiliations:** 1 Faculty of Dental Medicine, University of Porto (FMDUP), Rua Dr. Manuel Pereira da Silva, 4200-393 Porto, Portugal; 2 Barts & The London School of Medicine & Dentistry, Centre for Cutaneous Research, Blizard Institute of Cell and Molecular Science, Queen Mary University of London (QMUL), 4 Newark Street, Whitechapel, London, E1 2AT UK; 3 Department of Dental Implantology, Rey Juan Carlos University (URJC), Av. de Atenas, S/N, 28922 Alcorcón, Madrid Spain; 4 School of Engineering (EEUM), ALGORITMI Research Centre, LASI, University of Minho, Campus de Gualtar, 4710-057 Braga, Portugal; 5 Egas Moniz Center for Interdisciplinary Research (CiiEM), Egas Moniz School of Health & Science, Campus Universitário, Quinta da Granja, 2829-511 Caparica, Almada Portugal; 6 Division of Oral Diagnostics and Rehabilitation, Department of Dental Medicine, Karolinska Institutet, SE-14104 Huddinge, Sweden; 7 LAQV/REQUIMTE, Periodontology and Oral Surgery Department, Faculty of Dental Medicine, University of Porto (FMDUP), Rua Dr. Manuel Pereira da Silva, 4200-393 Porto, Portugal

**Keywords:** Botulinum Toxin, Biomarkers, Inflammation, Chronic conditions, Head and Neck, Clinical research

## Abstract

Botulinum toxin type A (BoNT) has emerged as a potential alternative to conventional therapies to many debilitating chronic diseases characterised by inflammatory states. However, the biological rationale remains ambiguous. Our review aimed to systematically assessed which biochemical biomarkers have been reported in clinical research to evaluate BoNT analgesic and mood-lifting effects in head and neck chronic conditions related to inflammation. We searched databases and registries between inception and September 29, 2023. Of the nine included studies, there were concerns about risk of bias for six studies. The leading biomarker with five studies was the calcitonin gene-related peptide (CGRP), followed by serotonin with two studies. Oxidative stress biomarkers were only reported in one study. Several important players in inflammatory processes and different immune cell classes have been evaluated in four studies. There was only one trial measuring changes in beta Tubulin and SNAP-25, and another study evaluating cutaneous neuropeptide substance-P. After BoNT, a significant effect was reported in six studies, including decrease in plasma levels of CGRP in chronic migraine and trigeminal neuralgia; serotonin decrease when collected from human tears in refractory intractable dry eye disease and increase in peripheral blood platelets in painful cervical dystonia associated to depression and anxiety; decrease in plasma concentration of markers of oxidative damage to proteins and increase in biomarkers for antioxidant power; decrease in expression of gene sets involved in inflammatory pathways and immune cells classes in the periosteum and metalloproteinase-9 molecule in the tears. BoNT seems to affect some biomarkers present in chronic inflammatory conditions. However, the certainty evidence found was very low to moderate. This study is registered on PROSPERO (CRD42023432131).

**Supplementary Information:**

The online version contains supplementary material available at 10.1007/s00702-024-02869-w.

## Introduction

The definition of inflammation is broad and continuously evolving (Charles et al. 2021), encompassing the response of living tissues to noxious stimulus, which entails a complex biological interplay of the immune, somatosensory, autonomic, and vascular systems (Matsuda et al. 2019). Currently, the term inflammation is used to describe three different types, namely traditional inflammation, neurogenic inflammation, and neuroinflammation (Charles et al. 2021; Matsuda et al. 2019).

The main signs characterising “traditional or classic” inflammation include redness, heat, swelling, pain, and the loss of function (Charles et al. 2021), with a repertoire of molecular, chemical, and cellular mediators of inflammation (e.g., histamine, cytokines, prostaglandins, and chemokines) (Charles et al. 2021; Matsuda et al. 2019). “Neurogenic inflammation” is triggered by the activation of the peripheral nervous system (PNS) c-fibres neurons (Charles et al. 2021; Marek-Jozefowicz et al. 2023). The neuronal activity results in the release of neuropeptides mainly including substance P (SP), calcitonin gene-related peptide (CGRP), and glutamate from peripheral nerves at different sites from the original stimulus (Matsuda et al. 2019). In addition, neurogenic inflammation may also be initiated by local inflammation events or by the orthograde or anterograde neuronal activation resulting in dorsal root reflex and the activation of the central nervous system (CNS) (Matsuda et al. 2019), which leads to functional changes such as increased vascular permeability and vasodilation (Charles et al. 2021). Lastly, “neuroinflammation” is described as a localised defence mechanism to injury, infection and trauma involving changes in cellular gene expression, and occasional in morphology, within neural tissue (Charles et al. 2021). Characteristic features include the activation of glial cells in dorsal root ganglia (DRG), spinal cord, and brain (Charles et al. 2021; Matsuda et al. 2019). In addition, neuroinflammation involve increased vascular permeability, leukocyte infiltration, increased production and release of inflammatory mediators including cytokines, chemokines, inducible nitric oxide synthase (iNOS), as well as reactive oxygen species (ROS), in both PNS and CNS (Matsuda et al. 2019; Fang et al. 2023).

The different types of inflammation play active roles in chronic conditions affecting a growing number of patients worldwide (Matsuda et al. 2019). So far, chronic inflammation has been implicated in a wide spectrum of diseases including metabolic disorders, allergies and respiratory diseases, cardiovascular diseases, arthritis and joint diseases, neurodegenerative disorders, and cancer (Mapunda et al. 2022). In particularly, neurogenic inflammation has been found to be strongly involved in scenarios of chronic pain such as migraine, and inflammatory conditions including psoriasis and asthma (Matsuda et al. 2019). Also, it has been suggested by compelling evidence that chronic neuroinflammation is a key pathological driver of many psychiatric illnesses, neurological diseases, chronic pain conditions, and traumatic brain injury (Corrigan et al. 2016).

Conventional treatment options to nonresolving inflammation are limited, often ineffective, and the required long-term challenging care contributes to the financial burden of chronic diseases (Fang et al. 2023). Therefore, the development of effective approaches for the prevention and treatment of inflammation-associated chronic conditions is urgent and it may need to target specific mechanisms within the different types of inflammation (Charles et al. 2021; Matsuda et al. 2019).

Botulinum toxin type A (BoNT) has showed great value in reducing headaches, inflammatory pain, and bacterial infection (Matsuda et al. 2019; Becker 2020). The BoNT ability to inhibit neurogenic inflammation by cleaving SNAP-25 (synaptosomal-associated protein of 25 kDa) and blocking the release of CGRP and other neuropeptides from peripheral C-fibre nerve endings is thought to have a critical therapeutic role in chronic migraine (Becker 2020). Other mechanisms that may also be important include the diminution of neurotransmitter and neuropeptide release in the trigeminal ganglion (potentially in the central terminals of the peripheral sensory neurons), the modulation of expression of certain receptors or ion channels (e.g., transient receptor potential vanilloid 1 (TRPV1), calcium (Ca^2^^+^), sodium (Na^+^)) in nociceptor cell membranes, and the regulation of pathways that mediate pain via gamma-aminobutyric acid (GABA) and opioid systems (Becker 2020; Hajj and Haddad 2021). In addition, the International Association for the Study of Pain (IASP) proposed BoNT as a third-line treatment for managing neuropathic pain (Finnerup et al. 2015). Although the IASP recommendations were considered weak, recent findings supported that BoNT suppressed the activation of microglia in the CNS on neuropathic pain (Li et al. 2021). The modulation of the development of neuroinflammation by inhibiting the overexpression of microglia-derived pro-inflammatory factors was also suggested to be involved in the reported therapeutic effects of BoNT on depression (Li et al. 2021).

In this direction, both clinical and preclinical trials have demonstrated that BoNT has analgesic, anti-inflammatory and mood lifting effects, mainly independent of its motor effect. However, the quality of evidence is not yet high enough to provide categorical assurance of the efficacy of BoNT therapy in specific chronic conditions (Finzi and Rosenthal 2014; Shi et al. 2020). Additionally, literature has shown BoNT mechanisms likely to be involved in inflammatory-associated chronic conditions, focusing on BoNT actions at the level of PNS, but also confirming indirect and direct effects on the CNS [Luvisetto 2021]. However, the biological rational of BoNT therapy remains ambiguous and further objective investigations are needed to improve our knowledge (Hajj and Haddad 2021).

Biomarkers are crucial to the rational development of diagnostics and therapeutics, although basic concepts regarding their use in research and clinical practice remains challenging to define, especially in the context of chronic diseases (Califf 2018; FDA-NIH Biomarker Working Group 2016). Noteworthy that the standardised Initiative on Methods, Measurement, and Pain Assessment in Clinical Trials (IMMPACT) recognised the importance of identifying valid and reliable biomarkers (Smith et al. 2017). Therefore, knowing which biomarkers have been used in available clinical studies, and BoNT effects, may serve as a useful starting point to promote a more coherent manner for measuring the therapeutic effects of BoNT in future research and pave the way to expand its clinical applications.

In this context, the aim of this systematic review was to identify and critically appraise which biomarkers have been reported in clinical research evaluating BoNT effects in each specific chronic condition associated to different types of inflammation. We have therefore focused on monitoring and pharmacodynamic/response classes of biochemical biomarkers and their level of changes in response to BoNT administration to detect evidence of its possible therapeutic effects.

## Methods

This study is registered on PROSPERO, number CRD42023432131 (Pereira. 2023). The Preferred Reporting Items for Systematic Reviews and Meta-Analyses (PRISMA) guidelines was followed for reporting this review (see Online Resource 1 for PRISMA 2020 checklist) (Page et al. 2020). The PICO framework was used to structure the reporting of eligibility criteria:

(P) Population—adult patients with head and neck chronic conditions related to classic inflammation and/or neurogenic inflammation and/or neuroinflammation; (I) Intervention—botulinum toxin (BoNT); (C) Comparisons: comparisons will be made regarding which biochemical biomarkers and biological sampling have been evaluated according to each specific chronic condition; (O) Outcome –key effect of BoNT on each biochemical biomarker, i.e., change in biomarkers level (increase, decrease or no change) following BoNT administration.

### Eligibility criteria

We included all published clinical studies either with a control group or without one and regardless of the sample size. All editorials, conferences abstracts, letters, review articles and systematic reviews and meta-analysis were excluded but have been screened for single eligible studies. There were no language restrictions, nor did we exclude studies based on the journal and date of publication.

The eligible studies had to meet all the following criteria: (1) Studies performed on human adults and without gender restrictions, (2) Studies on head and neck chronic conditions (migraine, arthritis, herpetic neuralgia, trigeminal neuralgia, temporomandibular joint pain, myofascial pain, keloids or hypertrophic scars, periodontitis, anxiety, chronic stress, depression, traumatic brain injury, intractable dry eye disease, ocular pain, rosacea, psoriasis, dermatitis, cephalalgia, and alopecia) that have been linked to inflammation, and/or neurogenic inflammation, and/or neuroinflammation, (3) Studies on BoNT (any dose, formulation, administration technique and injection-site) which involved biochemical biomarkers to evaluate outcomes.

We excluded studies on animals, in vitro and in silico disease models, studies evaluating BoNT effects and mechanisms in healthy individuals, or on acute conditions, studies related to muscles and glands hyperactivity rather than inflammation, on other body parts below head and neck, evaluating combined therapies or other interventions rather than BoNT, studies evaluating BoNT effects based solely on other tools/assessments rather than biochemical biomarkers, or when biomarker assessments were not reported following BoNT administration.

### Information sources and search strategy

For this systematic review we searched PubMed, Scopus, Web of Science databases. We searched US National Institutes of Health Ongoing Trials Register ClinicalTrials.gov (www.clinicaltrials.gov/), and the International Prospective Register of Systematic Reviews PROSPERO (www.crd.york.ac.uk/prospero/). The databases and registers were last searched on September 29, 2023. Additionally, we hand-searched the bibliographies of included and excluded articles to find additional studies not identified through the initial search strategy. The full search strategies for all databases, registers, and websites, including any filters and limits used, as well as the date when each source was last searched by two reviewers (INP, SD) are available on Online Resource 2.

### Selection process

Two researchers (INP, SD) independently and blinded screened titles and abstracts of all articles retrieved. In case of disagreement, consensus on which articles to screen full text was reached by discussion. When necessary, a third researcher was consulted to make the final judgement (GDC). In the second phase of the selection process, two researchers (INP, SD) independently screened full text articles identified as possibly relevant in the initial screening. Again, in case of disagreement, consensus was reached on inclusion or exclusion by open discussion and if necessary, the third researcher (GDC) was consulted. We imported titles and abstracts retrieved by the searches into Ryann software, a research collaboration platform for systematic reviews. Duplicate records were identified, manually reviewed, and then removed using Ryann automatic de‐duplication feature (see Online Resource 3 for full Ryann report). When we found non‐English language articles, we used Google Translate to determine potential eligibility.

### Data collection process

We developed a standardised data extraction table, which was pilot tested by two researchers (INP, SD) using four randomly selected studies. Two independent team members were involved in the data extraction. Any discrepancies were resolved through consensus and, when necessary, involved a third or fourth researcher. In cases of incomplete data (primary objective), attempts were made to contact the corresponding authors by web e-mail and following the pre-defined protocol (maximum of 3 attempts, 10-day intervals).

Data was extracted on first author name, year of publication, study design with assigned level of evidence based on those suggested by the latest Oxford Centre for Evidence-Based Medicine ratings (OCEBM Levels of Evidence Working Group 2011). We also sought for information relating to the characteristics of included studies and results as follows: specific population characteristics encompassing sample size (including sample size for each exposure group at each measurement point and included in analysis; number lost to follow up), sex distribution and age demographics, chronic condition/inflammatory state characteristics (tests or diagnostic criteria used, when reported), follow-up period, which biomarkers have been tested with unit and timing of measurement, as well as biological sampling or specimens, and any data on key BoNT exposure (anatomical site of intervention, toxin formulation and dose), and change in biomarkers (baseline and outcome assessments). For outcomes, we extracted event rates in each comparison group (dichotomous outcomes) and mean differences, or mean changes in final measurements from baseline assessments with associated standard deviations (or standard errors, 95% confidence intervals or relevant statistics, or P values) for each comparison group (continuous outcomes). In addition, we extracted data on other measurements of intervention effectiveness and clinical effects (improvement, aggravation, or no change in condition) and other characteristics of importance within the context of each study. When specific data point was entirely absent, we systematically documented and presented as "Not Reported" (NR) in our analysis. When an outcome was measured at multiple sites or subclasses, data from the site or subclass where effect is highest was included.

### Study characteristics, risk of bias, and certainty assessment

We evaluated the detailed methodology for all the included studies using the CASP critical appraisal checklists (Checklists et al. 2024), employing the CASP Randomised Controlled Trial (RCT) Standard Checklist and the CASP Cohort Standard Checklist when appraising a RCT or non-randomised study, respectively.

We assessed risk of bias of included RCTs using the revised Cochrane “Risk of bias” tool for RCT (RoB 2.0) (Sterne et al. 2019), employing the additional guidance and the latest version for crossover trials. Two researchers independently applied the RoB 2.0 official excel tool addressing five domains to each included RCT. The rationale for article appraisals was recorded at each stage, and for risk of bias assessment each domain was judged “low”, “some concerns”, or “high”. Any disagreement was resolved by consensus or adjudication of a third and fourth researcher. Following guidance given for RoB 2.0, for each specific outcome we created an overall summary “Risk of bias” judgement (low; some concerns; high). For each study the overall bias was given based on the highest bias score for each decision domain. A similar protocol was adopted to assess risk of bias of included studies that did not use randomisation to allocate interventions through an unofficial excel worksheet for the implementation of the Cochrane “risk of bias tool in non-randomised studies—of interventions” (ROBINS-I) (Marcolino 2020). This tool addresses seven domains and, for risk of bias assessment, each domain and overall bias was judged “low”, “moderate”, “serious”, “critical”, or “no information” (Sterne et al. 2016).

We used the five Grading of Recommendations Assessment, Development and Evaluation (GRADE) approach considerations to assess the certainty of the body of evidence for the prespecified outcomes (which biomarkers and BoNT key effect) (Schünemann et al. 2023). We assessed the certainty of evidence as high, moderate, low, or very low. All the decisions to down- or up-grade the certainty of studies were justified and recorded manually in summary of findings tables (Murad et al. 2017).

### Effect measures and syntheses methods

In line with the review protocol, we synthetised the evidence narratively and created tabular structures. First, we tabulated all extracted data according with the protocol for the complete and transparent reporting of the results and to provide a summary of the findings on the developed standardised table. Secondly, different tabular constructs were designed to provide studies characteristics, outcomes and a visual overview to compare data across studies for specific biomarkers used, each targeted condition, as well as specific biological sampling employed. Data was combined to try to conclude on the eligibility for measuring quantitative analysis.

## Results

**Fig. 1:**
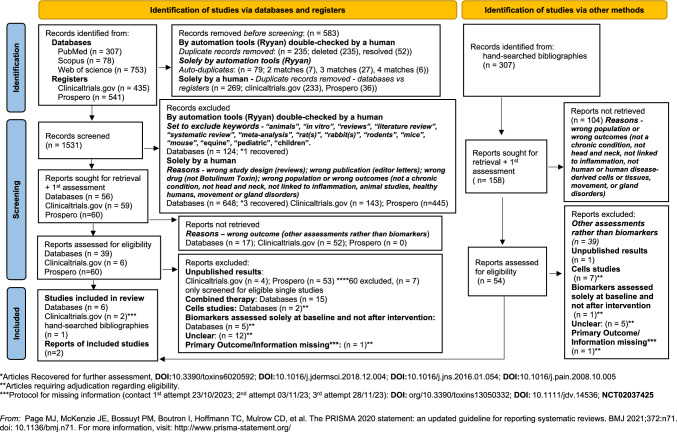
PRISMA 2020 flow diagram

We found 2114 records in databases and registers searching. After duplicates removal, we screened 1531 records, from which we sought for retrieval 175 full-text reports, and finally included eight studies (Zhang et al. 2020; Cernuda-Morollón et al. 2015; Dini et al. 2019; Cutrer et al. 2010; Karakulova and Loginova 2017; Choi et al. 2019; National Library of Medicine 2023; National Library of Medicine (US). Identifier NCT02037425. 2023). We also searched the bibliographies of included and excluded articles and we found one extra article that fulfilled inclusion criteria and two reports referring to studies already included (Gfrerer et al. 2022; Cady et al. 2014; Cutrer and Pittelkow 2006). Online Resource 3 displays the full Ryann report. Full list of citations that did not meet the inclusion criteria is available upon request. The process was schematised in the PRISMA Flow Diagram (Fig. 1).

From the reports assessed for eligibility, we excluded from our review 32 records requiring adjudication (Cutrer and Pittelkow 2006; Cernuda-Morollón et al. 2014; Leira et al. 2021; Domínguez et al. 2018; Moreno-Mayordomo et al. 2019; Domínguez Vivero et al. 2020; Xiaoxue et al. 2014; Gauglitz et al. 2012; Xiao et al. 2010, 2011; Hao et al. 2018; Park et al. 2019; Jeong et al. 2015; Shon et al. 2020; Zhang et al. 2020; Hubbard et al. 2016; Todberg et al. 2018; Kim et al. 2019, 2021; Bumb et al. 2013; Khatery et al. 2022; Aschenbeck et al. 2018; Philippova et al. 2021; Gazerani et al. 2009; Silva et al. 2014; Reyes et al. 2023; Borodic et al. 2014; Sebastianelli et al. 2023; Valente et al. 2021; Ozarslan et al. 2022; Tommaso et al. 2016; Lee et al. 2016), and we listed the reasons for exclusion in the Online Resource 4 tables. We have tried to contact the corresponding author for the trial with the registration ClinicalTrials.gov NCT01071096 to have more information on the reported histological examinations (National Library of Medicine (US). Identifier NCT01071096. 2023) Although we were unable to confirm the location for the skin punch biopsies, this was not a criterium for exclusion.

Of the nine studies included (Zhang et al. 2020; Cernuda-Morollón et al. 2015; Dini et al. 2019; Cutrer et al. 2010; Karakulova and Loginova 2017; Choi et al. 2019; National Library of Medicine (US). Identifier NCT01071096, 2023; National Library of Medicine (US). Identifier NCT02037425. 2023; Cady et al. 2014), two were randomised double blind placebo-controlled trials (Choi et al. 2019; National Library of Medicine (US). Identifier NCT01071096. 2023; Cady et al. 2014), one was a single case experimental design (Cutrer et al. 2010), and six were non-randomised clinical trials either with a control healthy group (Zhang et al. 2020; Dini et al. 2019; Karakulova and Loginova 2017), or a “within subject” follow-up design (Cernuda-Morollón et al. 2015; National Library of Medicine (US). Identifier NCT02037425. 2023; Gfrerer et al. 2022). From these, five studies generated effects for different subgroups of responders versus non-responders to BoNT treatment (Zhang et al. 2020; Cernuda-Morollón et al. 2015National Library of Medicine (US). Identifier NCT01071096. 2023; National Library of Medicine (US). Identifier NCT02037425. 2023; Gfrerer et al. 2022). Table 1 shows the characteristics of the included clinical trials. These were published between 2010 and 2022, with six out of nine studies reporting sources of funding (Cernuda-Morollón et al. 2015; Cutrer et al. 2010; Choi et al. 2019; National Library of Medicine (US). Identifier NCT01071096. 2023; National Library of Medicine (US). Identifier NCT02037425. 2023; Gfrerer et al. 2022). Sample-sizes ranged from 1 to 83 among 353 participants (61 males, 233 females and 59 unknown) of varying ages between 20 to 81 years old. Inclusion criteria varied among the studies, three studies targeted participants naive to BoNT treatments (n = 113) (Zhang et al. 2020; Dini et al. 2019; National Library of Medicine (US). Identifier NCT02037425. 2023), one study also reported two participants naive to BoNT among a study sample of 18 (Gfrerer et al. 2022), while another study included only one participant with history of repetitive BoNT treatment (Cutrer et al. 2010). OnaBoNT formulations were the most reported (Cernuda-Morollón et al. 2015; National Library of Medicine (US). Identifier NCT01071096. 2023, National Library of Medicine (US). Identifier NCT02037425. 2023; Gfrerer et al. 2022), with doses ranging from 2.5 to 195 units, depending on the targeted areas. The measurement time point for the specific biomarker used in each study ranged from 34 days to 7 months, with most studies performing the final assessment one month following the administration of BoNT (Cernuda-Morollón et al. 2015; Karakulova and Loginova 2017; Choi et al. 2019; Gfrerer et al. 2022).

**Table 1:**
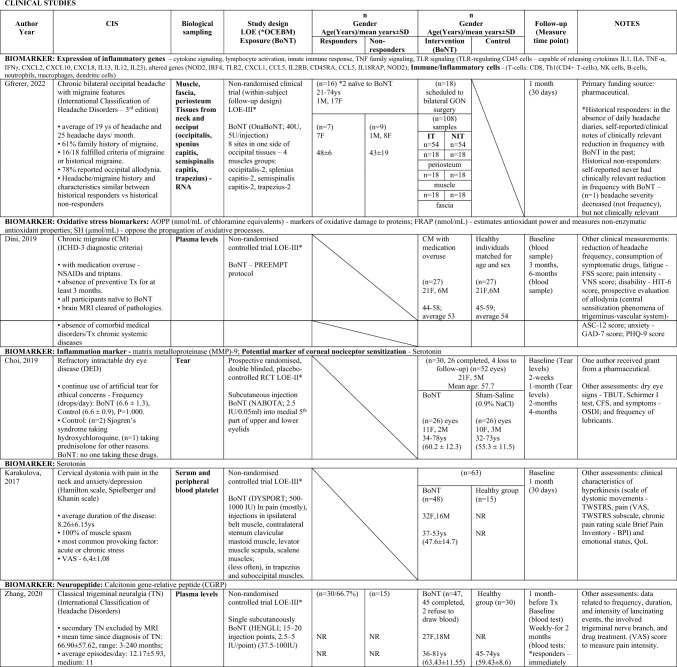

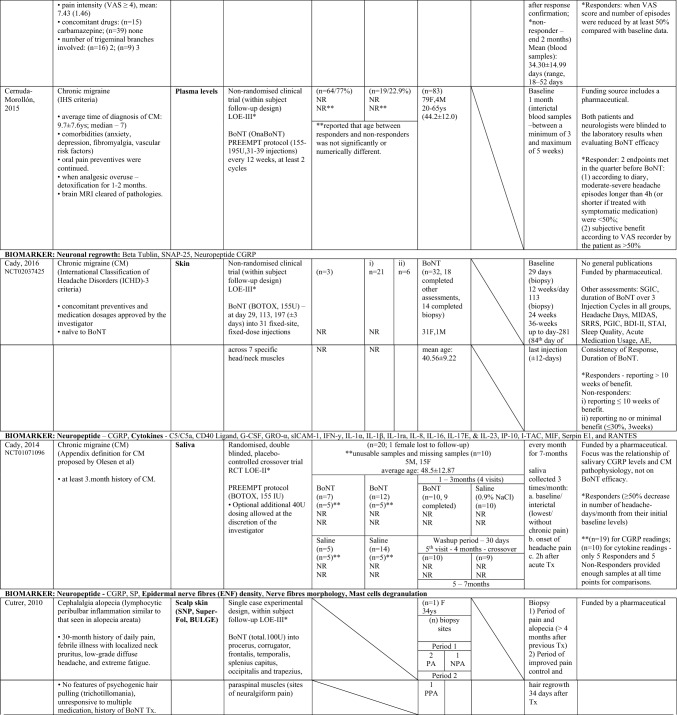
Summary of findings—Characteristics of the included clinical trials

*AE* adverse events, *AOPP* advanced oxidation protein products, *ASC-12* Allodynia Symptoms Checklist 12, *BDI-II* Beck Depression Inventory II, *BoNT* botulinum toxin, *BULGE* bulge area of hair follicles, *CGRP* calcitonin gene-related peptide, *CIS* chronic inflammatory state, *CM* chronic migraine, *CFS* corneal fluorescein staining, *DED* dry eye disease, *ENF* epidermal nerve fibre, *F* Female, *FRAP* ferric reducing antioxidant power, *FSS* Fatigue Severity Scale, *GAD–7* Generalized Anxiety Disorder, *GON* greater occipital nerve, *GRO-α* Growth Regulated Oncogene alpha, *G-CSF* Granulocyte Colony Stimulating Factor, *HIT-6* Headache Impact Test, *ICHD-3* International Classification of Headache Disorder 3rd edition, *IFN-y* Interferon gamma, *IHS* international headache society, *IL* interleukin, *IP-10* Interferon Gamma-Induced Protein 10, *IQ* interquartile range, *IT* injected tissues, *I-TAC* Interferon-inducible T cell-α chemoattractant, *LOE* level of evidence (* according to Oxford Centre of Evidence-Based Medicine ranking), *M* male, *MIDAS* Migraine Disability Assessment Scale, *MIF* Macrophage Migration Inhibitory Factor, *MMP* matrix metalloproteinase, *MRI* magnetic resonance imaging, *NIT* non-injected tissues, *NPA* no pain or alopecia, NR not reported, *NSAIDs*, non-steroidal anti-inflammatory drugs, *OSDI* ocular surface disease index, *PA* pain and alopecia, *PGIC* Physician Global Impression of Change, *PHQ–9* Patient Health Questionnaire, *PPA* previous pain and alopecia, *QoL* quality of life, *RANTES* Regulated Upon Activation Normal T-cell Expressed, *RCT* randomised controlled trial, *SD* Standard deviation, *SGIC* Subject Global Impression of Change, *SH* thiolic groups, *sICAM-1* Soluble Intercellular Adhesion Molecule, *SNAP-25* synaptosomal-associated protein of 25 kDa, *SNP* subepidermal neural plexus, *SP* substance-P, *SRRS* Social Readjustment Rating Scale, *STAI* State-Trait Anxiety Inventory, *Super-Fol* superficial dermis surrounding a hair follicle, *TBUT* tear film break-up time, *TGF-β1* transforming growth factor β1, *TN* trigeminal neuralgia, *TNF-α* tumor necrosis factor alfa, *TWSTRS* spasmodic torticollis scoring scale West Toronto, *Tx* treatment, *U* Units, *VNS* Verbal Numeric Scale, *VAS* visual analog scale, *Ys* Years

### Risk of bias assessment

**Fig. 2:**
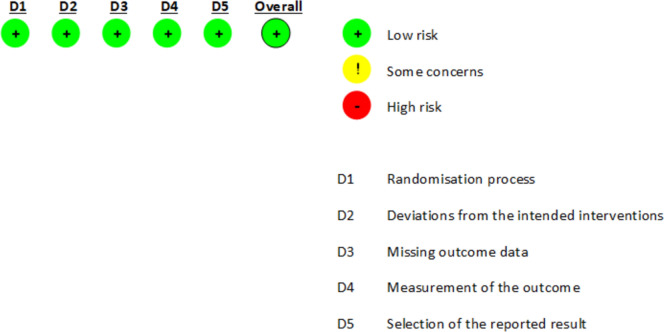
RoB 2.0 tool—risk of bias for individually randomised parallel-group trial (Choi 2019)

**Fig. 3:**
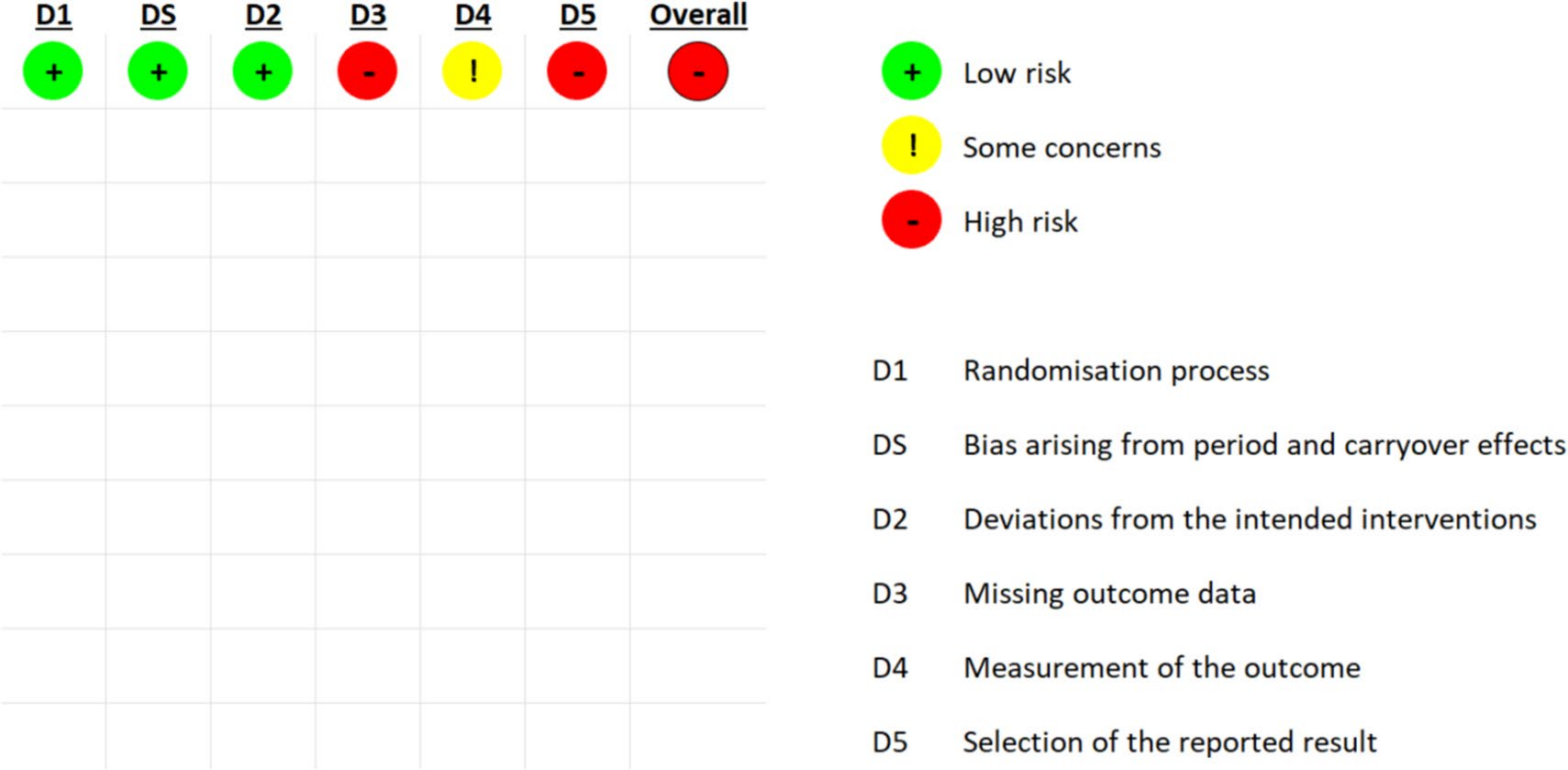
RoB 2.0 test version tool—risk of bias for crossover trials (Cady 2014)

**Fig. 4:**
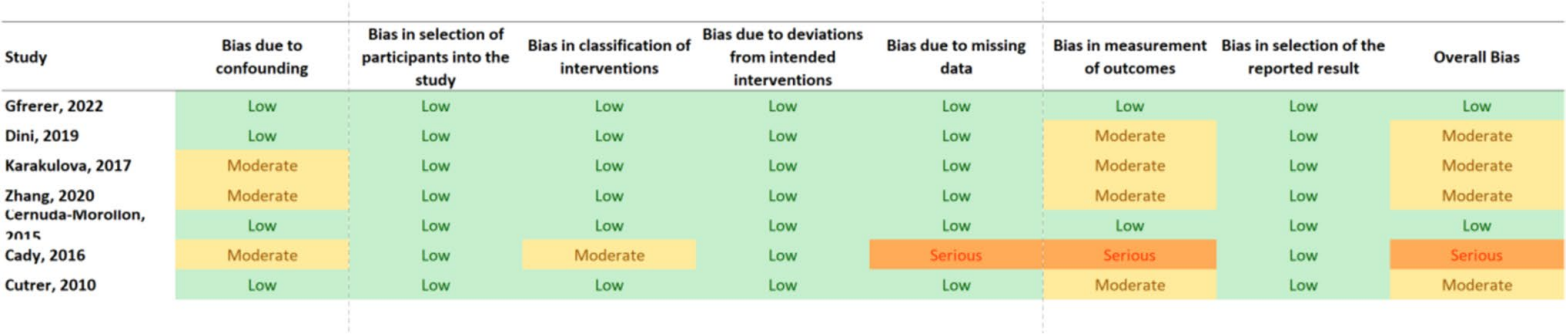
Unofficial excel worksheet—ROBINS-I assessment tool for non-randomised clinical trials

A summary of the risk of bias assessments is provided in Figs. 2, 3, 4. In terms of overall risk of bias, there were concerns for most studies (6/9), with two of these assessed as at high or serious risk of bias (National Library of Medicine (US). Identifier NCT01071096. 2023; National Library of Medicine (US). Identifier NCT02037425. 2023). Justifications for assessments were recorded in the CASP checklists, which are available upon request. Online Resource 5 provides a shortened report.

### Results of individual studies

Table 2 shows a summary of the outcomes of the included studies regarding BoNT key effects on each reported biomarker. To facilitate comparisons, Table 3 illustrates data across studies for specific biomarkers used, each targeted condition, as well as specific biological sampling employed.

**Table 2.**
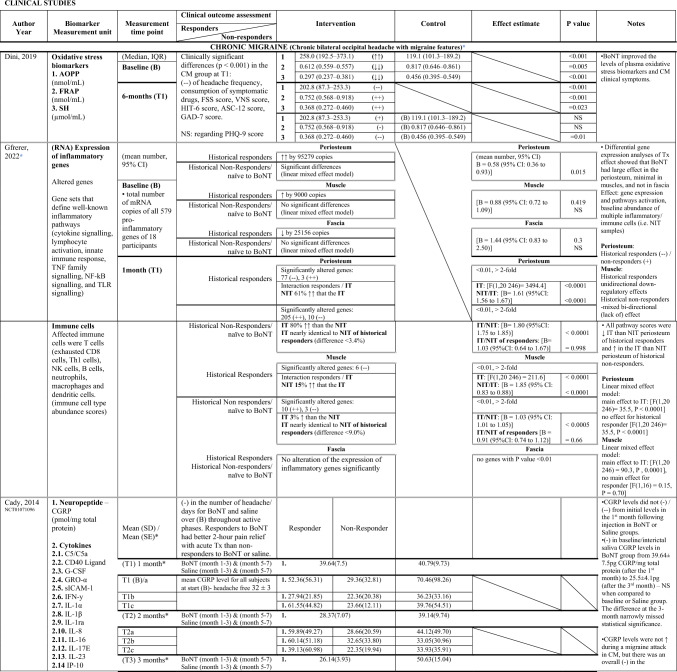

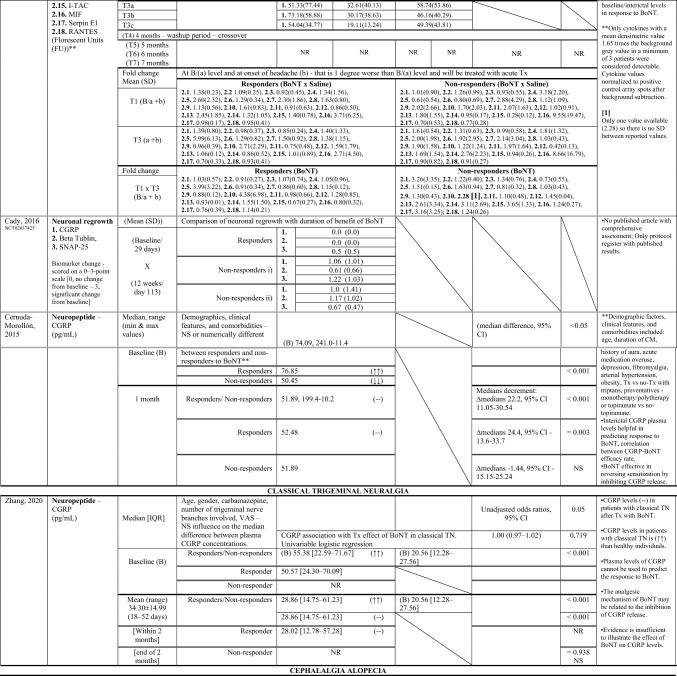

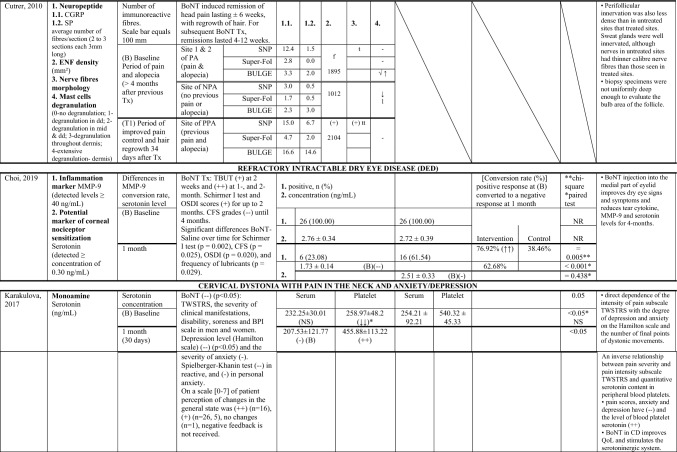
Summary of findings—Outcomes of the included clinical trials

**↑**, higher; **↓** lower; **↑↑**, significantly higher; **↓↓**, significantly lower; **(+ +)**, increased significantly; **( +)** increased but not significantly; **(–)**, decreased significantly; **(-)**; decreased but not significantly; **i)**, ≤ 10 weeks of benefit; **ii)**, no or minimal benefit (≤ 30%, 3 weeks); **a,** baseline/interictal (lowest/without chronic pain); **b,** onset of headache pain; **c,** 2 h after acute Tx; *t* thin, *tt* thick, *f* fragmented, *dd* deep dermis, *AOPP* advanced oxidation protein products, *BoNT* botulinum toxin, *BULGE* bulge area of hair follicles, *CD* Cervical dystonia, *CRP* C-reactive protein, *CGRP* calcitonin gene-related peptide, *CI* confidence interval, *CM* chronic migraine, *CFS* corneal fluorescein staining, *DED* dry eye disease, *ENF* epidermal nerve fibre, *F* Female, *FRAP* ferric reducing antioxidant power, *GON* greater occipital nerve, *GRO-α* Growth Regulated Oncogene-alpha, *G-CSF* Granulocyte-Colony Stimulating Factor, *IFN-y* Interferon-gamma, *IL* interleukin, *IP-10* Interferon Gamma-Induced Protein-10, *IQ/[IQR]* interquartile range, *IT* injected tissues, *I-TAC* Interferon-inducible T cell-α chemoattractant, *log2FC* log2-fold change, *M* male, *MIF* Macrophage Migration Inhibitory Factor, *MMP* matrix metalloproteinase, *MRI* magnetic resonance imaging, *NIT* non-injected tissues, *NPA* no pain/alopecia, NR not reported, *NS* not statistically significant, NSAIDs, non-steroidal anti-inflammatory drugs, PA pain + alopecia, *PGIC* Physician Global Impression of Change, *PHQ–9* Patient Health Questionnaire, *PPA* previous pain + alopecia, *QoL* quality-of-life, *RANTES* Regulated Upon Activation Normal T-cell Expressed, *RCT* randomised controlled trial, *SE* standard error, *SD* Standard deviation, *SH* thiolic groups, sICAM-1 Soluble Intercellular Adhesion Molecule, *SNAP-25* synaptosomal-associated protein of 25 kDa, SNP subepidermal neural plexus, *SP* substance-P, Super-Fol superficial dermis surrounding hair follicle, *TBUT* tear film break-up time, *TGF-β1* transforming growth factor-β1, *TN* trigeminal neuralgia, *TNF-α* tumor necrosis factor-alfa, TWSTRS spasmodic torticollis scoring scale West Toronto, *Tx* treatment, *U* Units, *VNS* Verbal Numeric Scale, *VAS* visual analog scale, *Ys* Years

**Table 3:**
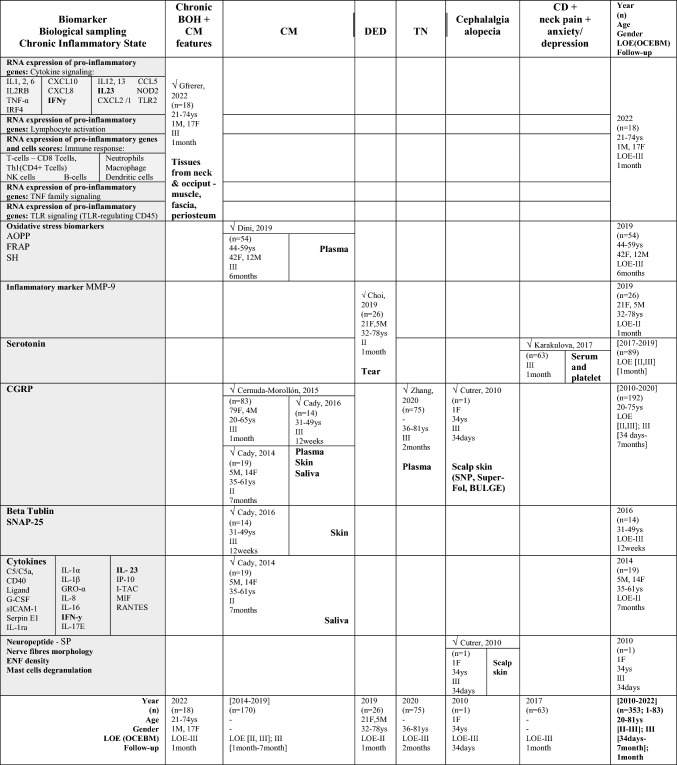
Biomarkers Versus Chronic Inflammatory State Versus Biological sampling

*AOPP* advanced oxidation protein products, *BOH* bilateral occipital headache, *BoNT* botulinum toxin, *BULGE* bulge area of hair follicles, *CD* Cervical dystonia, *CM* chronic migraine, *CRP* C-reactive protein, *CGRP* calcitonin gene-related peptide, CIS chronic inflammatory state, *CM* chronic migraine, DED dry eye disease, *ENF* epidermal nerve fibre, *F* Female, *FRAP* ferric reducing antioxidant power, *GON* greater occipital nerve, GRO-α Growth Regulated Oncogene alpha, *G-CSF* Granulocyte Colony Stimulating Factor, *IFN-y* Interferon gamma, *IL* interleukin, *IP-10* Interferon Gamma-Induced Protein 10, I-TAC Interferon-inducible T cell-α chemoattractant, *LOE* level of evidence (* according to Oxford Centre of Evidence-Based Medicine ranking), *M* Male, *MIF* Macrophage Migration Inhibitory Factor, *MMP* matrix metalloproteinase, *PA* pain and alopecia, *PPA* previous pain and alopecia, *RANTES* Regulated Upon Activation Normal T-cell Expressed, *SH* thiolic groups, *sICAM-1* Soluble Intercellular Adhesion Molecule, *SNAP-25* synaptosomal-associated protein of 25 kDa, SNP subepidermal neural plexus, *SP* substance-P, *Super-Fol* superficial dermis surrounding a hair follicle, *TGF-β1* transforming growth factor β1, *TN* trigeminal neuralgia, *TNF-α* tumor necrosis factor alfa, *Ys* Years

In line with what we anticipated, a meta-analysis could not be undertaken or would result in non-informative estimates due to the heterogeneity of chronic conditions, study designs features, biological sampling, biomarkers reported, and other outcome measures expressed on different scales or units of measurement.

### Results of synthesis

Several biomarkers have been identified and will be reported as follows:

Biomarkers—(A) biological sampling, (B) BoNT key effect, (C) bias in the results

#### CGRP

The leading biomarker assessed in five studies (n = 192) was the CGRP (Zhang et al. 2020; Cernuda-Morollón et al. 2015; Cutrer et al. 2010; National Library of Medicine (US). Identifier NCT02037425. 2023; Cady et al. 2014). This neuropeptide was primarily evaluated for chronic migraineurs (n = 116) treated with BoNT within three studies (Cernuda-Morollón et al. 2015; National Library of Medicine (US). Identifier NCT02037425. 2023; Cady et al. 2014). The remaining 76 participants suffered mostly from trigeminal neuralgia (n = 45) (Zhang et al. 2020), 30 were their healthy counterparts, and only one single case experiment reported CGRP levels in cephalalgia alopecia with lymphocytic peribulbar inflammation similar to that seen in alopecia areata (Cutrer et al. 2010).

A) Overall, the most common biological sampling point reported was the plasma (n = 212) (Zhang et al. 2020; Cernuda-Morollón et al. 2015; Dini et al. 2019). For CGRP, the plasma was selected in two studies, one in chronic migraine (n = 83) (Cernuda-Morollón et al. 2015), and the other one in trigeminal neuralgia (n = 75) (Zhang et al. 2020). Other samplings used for CGRP analysis, included saliva (n = 19) (Cady et al. 2014), skin (n = 14) (National Library of Medicine (US). 2023), and specifically the subepidermal neural plexus (SNP), superficial dermis surrounding a hair follicle (Super-Fol), and the bulge area of hair follicles (BULGE) within the scalp (n = 1) (Cutrer et al. 2010).
B) Two trials (n = 128 intervention, n = 30 control) reported that plasma CGRP concentration (pg/mL) significantly decreased following treatment with BoNT, when compared to healthy participants or to those without a favourable clinical response (Zhang et al. 2020; Cernuda-Morollón et al. 2015); the mean period for the collection of peripheral blood samples was 32.1 days. Of these, the study including only participants with classical chronic migraine suggested that interictal CGRP levels may be predictive of treatment response (Cernuda-Morollón et al. 2015), while the study providing measurements in trigeminal neuralgia patients and healthy controls demonstrated the opposite (Zhang et al. 2020). In another trial (n = 19), CGRP levels (pmol/mg total protein) were measured in saliva of chronic migraineurs following BoNT and saline administration (Cady et al. 2014). This exploratory pilot study also reported a decrease in baseline/interictal saliva CGRP levels for participants receiving BoNT, although this difference was only observed after the third month and narrowly missed statistical significance when compared to placebo-control group. The same author conducted another clinical study and reported no significant CGRP change levels (0–3-point scale) in the skin of 14 patients suffering from chronic migraine at 12 weeks after BoNT treatment (National Library of Medicine (US). 2023); the biomarker change level scores were lower for patients classified as “responders”. The last study focusing on CGRP, calculated the average number of fibres per section (two to three sections each 3 mm long, per biopsy specimen) within the scalp of a single female patient diagnosed with cephalgia alopecia (Cutrer et al. 2010). At 34 days after receiving BoNT, the number of immunoreactive CGRP fibres were higher, specially at the BULGE area.
C) One non-randomised clinical trial and one crossover RCT evaluating CGRP in chronic migraine were judged at low and high risk of bias, respectively (Cernuda-Morollón et al. 2015; National Library of Medicine (US). Identifier NCT01071096. 2023; Cady et al. 2014). Another non-randomised clinical trial in chronic migraine was judged at serious risk of bias, given the lack of information on participants baseline characteristics (e.g., potential comorbidities and medications used approved by the investigator) and the collection of data at different time points (not at the time of intervention), being unclear if it was possible to avoid differential misclassification (National Library of Medicine (US). Identifier NCT02037425. 2023). There were also some concerns for the remaining studies in different clinical settings (Zhang et al. 2020; Cutrer et al. 2010). When evaluating CGRP level in trigeminal neuralgia scenarios, we cannot exclude the risk of bias due to confounding and bias in measurement of outcomes (Zhang et al. 2020). As for the CGRP in the context of cephalgia alopecia (Cutrer et al. 2010), the moderate risk of bias was only ascribed to the potential bias in measurement of outcomes, i.e., it was unclear if the risk surrounding classification of the biomarker change on a scale could be considered negligible (see Online Resource 5 for detailed explanations for the judgements).

#### Serotonin

The second biomarker involving a higher number of participants (n = 89) was the monoamine transmitter serotonin reported in two controlled studies evaluating the effect of BoNT in different conditions (Karakulova and Loginova 2017; Choi et al. 2019).

A. Serotonin was collected from the tears of 26 subjects with refractory intractable dry eye disease (DED) or harvested from the serum and peripheral blood platelet (n = 63) when focusing on cervical dystonia with pain in the neck and associated to depression and anxiety (Karakulova and Loginova 2017; Choi et al. 2019).
B. The concentration of serotonin (ng/mL) was measured one month after BoNT treatment (Karakulova and Loginova 2017; Choi et al. 2019). When collected from the tears (n = 13 intervention, n = 13 saline control), serotonin concentrations detected ≥ 0.30 ng/mL were reduced in comparison to baseline (Choi et al. 2019); this difference was only significant for the BoNT group. Serum serotonin levels were reduced after BoNT therapy and significantly increased when harvested from peripheral blood platelets (n = 48 intervention, n = 15 healthy subjects) (Karakulova and Loginova 2017).
C. One individually parallel-group RCT and one non-randomised controlled clinical trial reported on serotonin levels. The first was judged at low risk of bias (Choi et al. 2019), whereas the latter was considered at moderate risk owning to bias due to confounding and bias in measurement of outcomes (Online Resource 5) (Karakulova and Loginova 2017).

#### Oxidative stress biomarkers

Oxidative stress biomarkers such as advanced oxidation protein products (AOHH), ferric reducing antioxidant power (FRAP), and thiol groups (SH), were only reported in one study for chronic migraine enrolling 54 participants (Dini et al. 2019).

A) Oxidative damage markers AOPP, FRAP (that estimates antioxidant power and measures non-enzymatic antioxidant properties), and SH (which oppose the propagation of oxidative processes) were harvested from the plasma of 27 chronic migraineurs reporting medication overuse and 27 healthy participants.
B) Plasma concentration measurements of AOPP (nmol/mL of chloramine equivalents), FRAP (nmol/mL), and SH (µmol/mL) took place six months after BoNT administration (n = 27 intervention, n = 27 control). An above-average final assessment demonstrated that BoNT significantly reduced AOPP (P < 0.001) and significantly increased FRAP (P < 0.001) and SH (P = 0.023). At six months, AOPP and FRAP levels were normalised, while SH concentration remained significantly lower when compared to healthy controls.
C) One non-randomised study was judged at moderate risk due to potential bias in measurement of outcomes (Online Resource 5).

#### Players in inflammatory processes and immune cell classes

Several important players in inflammatory processes such as pathways underlying adaptative and innate immune response, lymphocyte activation, cytokine and chemokine signalling, as well as different immune cell classes have been evaluated in four studies (n = 64) in cephalgia alopecia, refractory intractable DED, chronic migraine and chronic bilateral occipital headache with migraine features (Cutrer et al. 2010; Choi et al. 2019; National Library of Medicine (US). Identifier NCT01071096. 2023; Gfrerer et al. 2022).

A) The most recent study (2022) provided an inflammatory gene expression analysis based on 108 tissue samples from the neck and occiput and segregated these tissues by fascia, muscles, and periosteum (Gfrerer et al. 2022). This study reported a panel of 579 inflammation-related genes and multiple inflammatory/ immune cells scores. Another study performed scalp biopsies in a 34-year-old female and reported on mast cells degranulation (Cutrer et al. 2010). In addition, studies also focused on the matrix metalloproteinase-9 (MMP-9) inflammatory marker in tear samples from 52 human eyes (Choi et al. 2019), and samples for cytokine levels in saliva, namely C5/C5a, CD40 Ligand, G-CSF, GRO-α, sICAM-1, interferon gamma (IFN-y), interleukin (IL)-1α, 1β, 1ra, 8, 16, 17E, & 23, interferon gamma-induced protein 10 (IP-10), I-TAC, MIF, serpin E1, and regulated upon activation normal T-cell expressed (RANTES) (National Library of Medicine (US). Identifier NCT01071096. 2023).
B) After 34 days, BoNT completely inhibited the mast cells degranulation observed in non-treated areas previously with/without associated pain and alopecia in the scalp of one female patient (Cutrer et al. 2010). The study performing targeted transcriptome analyses within occiput and neck tissues (n = 18 participants, n = 108 samples), reported that 30 days after BoNT injections there were significant alterations in the expression of inflammatory genes in the periosteum, minimal in muscle and none in fascia (Gfrerer et al. 2022). Focusing on the periosteum, the expression of inflammatory genes in non-injected sites was significantly lower in participants without a previous positive response to BoNT than in historical responders. After BoNT treatment, in historical responders’ periosteum, BoNT significantly decreased the expression of most significantly altered genes and gene sets involved in inflammatory pathways, whereas in historical non-responders it increased gene expression, but not significantly (approximately to the level of the historical responders non-injected site) (Gfrerer et al. 2022). This study, providing a differential gene expression analysis of BoNT treatment effect in chronic bilateral occipital headache, also calculated the immune cell type abundance scores and reported the same response pattern (Gfrerer et al. 2022). This means that in non-injected samples there was abundance of multiple immune cells such as T-cells (CD8, type 1 T helper (Th1): CD4 + T-cells), NK cells, B-cells, neutrophils, macrophages, and dendritic cells, which were higher in the periosteum of historical responders than in the historical non-responders. One month after BoNT administration, the abundance was significantly decreased in the historical responders’ periosteum and increased (although not significantly) in the historical non-responders´ periosteum. In addition, MMP-9 concentration levels ≥ 40 ng/mL in tear samples of subjects with refractory intractable DED (n = 13 intervention, n = 13 placebo) significantly decrease after one month when participants received either BoNT or saline, although the percentage (conversion rate) was significantly higher in the BoNT group (Choi et al. 2019). Furthermore, cytokines concentration (florescent unit) in the saliva of chronic migraineurs (n = 9 BoNT, n = 10 placebo), detectable when mean densimetric value was 1.65 times the background grey value in a minimum of three patients (National Library of Medicine (US). Identifier NCT01071096. 2023). Mean fold changes and associated standard deviations were reported in responders versus non-responders, between first or third treatment months with BoNT and saline, as well as between first and third treatment months with BoNT. Overall, cytokines observed among the maximum values included the macrophage migration inhibitory factor (MIF), soluble intercellular adhesion molecule one (sICAM-1), IL-8, and interferon-inducible T cell alpha chemoattractant (I-TAC), while minimum figures included the granulocyte colony stimulating factor (G-CSF), I-TAC, Serpin E1, IL-17E, and growth regulated oncogene alfa (GRO-α).
C) Two exploratory “within subject” follow-up studies (n = 19), and the two RCTs (n = 45) presented discrepant judgement of risk of bias. The only study evaluated at high risk of bias was the protocol with the RCT crossover design (National Library of Medicine (US). Identifier NCT01071096. 2023), due to imprecision and reporting bias with laboratory errors related to processing of samples for cytokine levels in saliva, and hence missing data and re-processing of samples requirements (Online Resource 5).

#### Substance P

Cutaneous SP was only evaluated in a single patient suffering from cephalgia alopecia by assessing the average number of immunoreactive fibres per Sect. (2–3 sections each 3 mm long, per biopsy specimen) (Cutrer et al. 2010).

A. Scalp biopsies were performed to evaluate SNP, Super-Fol, and BULGE areas.
B. 34 days after BoNT, SP-positive nerve fibres were considerably higher along the SNP, at the surface and bulge area in areas previously with/without associated pain and alopecia.
C. Potential bias in measurement of outcomes resulted in the overall score of moderate risk of bias in this single case study.

#### Nerve fibres

The study in a 34-year-old female also reported on epidermal nerve fibres morphology and density observations (Cutrer et al. 2010).

A) The nerve fibres were evaluated within the scalp.
B) 34 days following BoNT, the nerve fibres increased density (ENF/mm^2^), thickness of the calibre, and were normal in appearance, with full nerve bundles coursing from deep dermis and forming a robust SNP. The study also reported that epidermal nerve fibres were abundant and uniformly distributed, demonstrating higher density in the treated than the untreated areas.
C) The single case experimental study was judged at moderate risk of bias (Online Resource 5).

#### Neuronal regrowth

There was only one clinical trial, including 30 patients suffering from chronic migraine, providing histological examinations of neural changes associated with regeneration of terminal neuronal endplates measured through changes in beta Tubulin and SNAP-25 (National Library of Medicine (US). Identifier NCT02037425. 2023).

A) Skin biopsies were performed at 12 weeks after BoNT.
B) The assessment of mean changes in beta Tubulin and SNAP-25 were scored on a 0–3-point scale. There were no significant changes reported, particularly for patients with > 10 weeks of clinical benefit.
C) The non-randomised trial was judged at moderate risk of bias for domain 1 (covering confounding and focusing on issues before the interventions start) and domain 3 (relating to the intervention itself). Domains concerning to issues after the interventions start, such as domain 5 (missing data, given that only a subset of subjects (14/30) completed the endpoint of interest–neuronal regrowth) and domain 6 (measurement of outcomes) were considered at serious risk of bias (Online Resource 5).

### Certainty of evidence

Overall, the evidence from the two RCTs was downgraded one step for imprecision (Choi et al. 2019), or two to three steps (National Library of Medicine (US). Identifier NCT01071096. 2023; Cady et al. 2014), once for bias and once or twice for imprecision. One non-randomised study was upgraded for large effect based on a well-done trial without important risk of bias or other limitations (Gfrerer et al. 2022). The remaining studies were kept at the initial low certainty evidence score (Zhang et al. 2020; Cernuda-Morollón et al. 2015; Dini et al. 2019; Cutrer et al. 2010; Karakulova and Loginova 2017), or very low if they presented serious risk of bias (National Library of Medicine (US). Identifier NCT02037425. 2023). In summary: CGRP, very low to low certainty evidence; SEROTONIN, low to moderate certainty evidence; OXIDATIVE STRESS BIOMARKERS, low certainty evidence; PLAYERS IN INFLAMMATORY PROCESSES, low to moderate certainty evidence; IMMUNE CELL CLASSES, very low to moderate certainty evidence; SUBSTANCE P, low certainty evidence; NERVE FIBRES, low certainty evidence; NEURONAL REGROWTH, very low certainty evidence. See summary of findings table with the certainty of evidence assessment (Table 4). Online Resource 5 provides the explanations for the judgements.

**Table 4:**
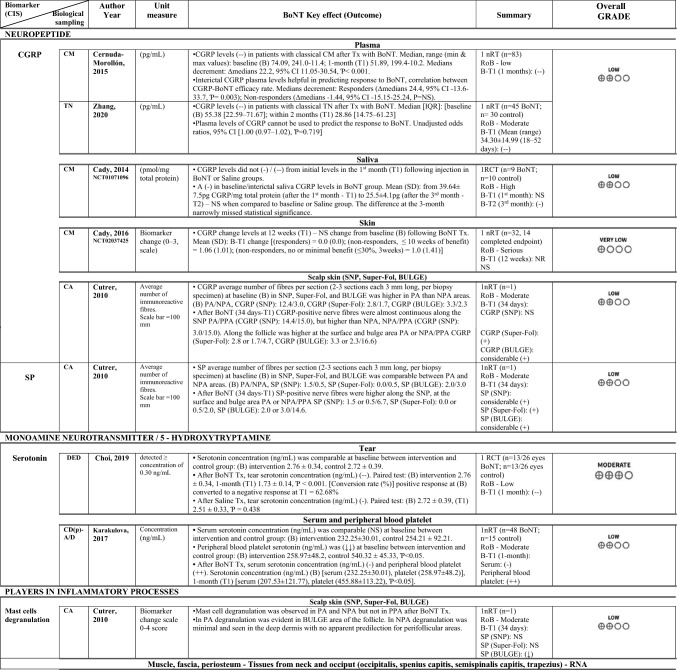

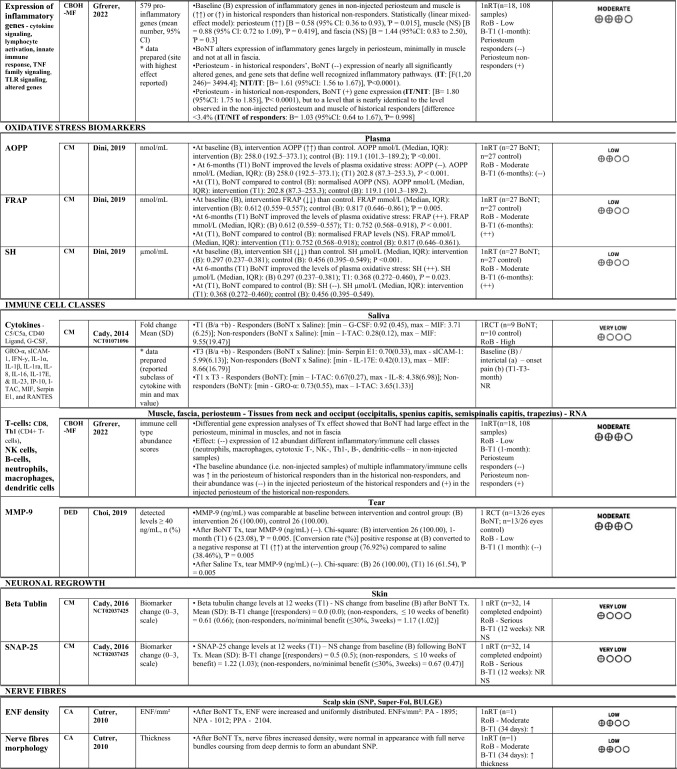
GRADE certainty assessment

↑, higher; ↓ lower; ↑↑, significantly higher; ↓↓, significantly lower; (+ +), increased significantly; ( +) increased but not significantly; (–), decreased significantly; (–); decreased but not significantly; (i), reporting ≤ 10 weeks of benefit; (ii), reporting no/minimal benefit (≤ 30%, 3 weeks); *AOPP* advanced oxidation protein products; *BoNT* botulinum toxin, *BULGE* bulge area of hair follicles, *CD(p)-A/D* Cervical dystonia with pain in the neck and anxiety/depression, *CRP* C-reactive protein, *CGRP* calcitonin gene-related peptide, *CA* cephalgia alopecia, *CBOH-MF* Chronic bilateral occipital headache with migraine features, *CI* confidence interval, *CIS* chronic inflammatory state, *CM* chronic migraine, *DED* dry eye disease, *ENF* epidermal nerve fibre,, *FRAP* ferric reducing antioxidant power, GON greater occipital nerve, GRO-α Growth Regulated Oncogene alpha, *G-CSF* Granulocyte Colony Stimulating Factor, *IFN-y* Interferon-gamma, IL interleukin, IP-10 Interferon Gamma-Induced Protein-10, IQ/[IQR] interquartile range, IT injected tissues, *I-TAC* Interferon-inducible T cell-α chemoattractant, log2FC log2 fold change, *MIF* Macrophage Migration Inhibitory Factor, *MMP* matrix metalloproteinase, *NIT* non-injected tissues, NPA no pain/alopecia, *NR* not reported, nRT non-randomised clinical trial, NS not statistically significant, *PA* pain + alopecia, *PPA* previous pain + alopecia, RANTES Regulated Upon Activation Normal T-cell Expressed, *RCT* randomised controlled trial, RoB Risk of bias, SE standard error, *SD* Standard deviation, *SH* thiolic groups, sICAM-1 Soluble Intercellular Adhesion Molecule, *SNAP-25* synaptosomal-associated protein 25 kDa, SNP subepidermal neural plexus, *SP* substance-P, Super-Fol superficial dermis surrounding a hair follicle, *TBUT* tear film break-up time, *TGF-β1* transforming growth factor-β1, *TN* trigeminal neuralgia, *TNF-α* tumor necrosis factor-alfa, *Tx* treatment, *U* Units

KEY:

GRADE certainty ratings

Very low: The true effect is probably markedly different from the estimated effect

Low: The true effect might be markedly different from the estimated effect

Moderate: The authors believe that the true effect is probably close to the estimated effect

High: The authors have a lot of confidence that the true effect is similar to the estimated effect

## Discussion

The main results of this systematic review show that after BoNT administration in humans suffering from inflammation-associated chronic conditions, a significant effect was reported in levels of some biomarkers, including CGRP (very low to low certainty evidence), serotonin (low to moderate certainty evidence), oxidative stress biomarkers (low certainty evidence), expression of gene sets involved in inflammatory pathways and immune cells classes as well as metalloproteinase-9 molecule (very low to moderate certainty evidence).

Although we need to exercise caution in interpreting the findings of this review because the meaning of biomarkers response is complex, not straightforward, and is based on a small number of studies, mostly with concerns about risk of bias, these appear to be largely in line with the current literature indicating that BoNT not only can reverse clinical features associated to the chronic conditions under investigation, as the mechanisms underlying an analgesic and mood lifting effect goes beyond the recognised toxin capacity to inhibit the presynaptic release of acetylcholine at the neuromuscular junction.

From this review, it seems like the most studied scenario evaluating BoNT effect with biomarkers was the prevention of chronic migraine, which may be explained by the fact that BoNT is a well-established and licensed treatment for this condition. In contrast, BoNT has been approved to treat cervical dystonia since 2000, being nowadays the first line of treatment, but we only found one study in cervical dystonia with pain in the neck, anxiety, and depression (Karakulova and Loginova 2017). This may be due to a paradigm shift in clinical research in BoNT effects, that is now focusing on the sensory aspects of cervical dystonia symptoms, including pain and mental illness that have been linked to inflammation (Kaji et al. 2018). BoNT is also an emerging therapy for neuropathic pain, and we found one study in trigeminal neuralgia and one RCT focusing on refractory intractable DED (Zhang et al. 2020; Choi et al. 2019), which is characterised by inflammation and features of neuropathic pain, such as pain resulting from nerve dysfunction. The other remaining condition was cephalgia alopecia with lymphocytic peribulbar inflammation, which is currently set on the premise that headaches result in the repeated activation of trigeminal and upper cervical branches implicated in the innervation of hair cells, triggering neurogenic inflammation, and leading to hair loss and disruption of immune system regulation (Cutrer et al. 2010).

The outcomes of this review suggest that after BoNT a significant effect was reported in six studies (Zhang et al. 2020; Cernuda-Morollón et al. 2015; Dini et al. 2019; Karakulova and Loginova 2017; Choi et al. 2019; Gfrerer et al. 2022). Significant key effects reported included a decrease in plasma levels of CGRP in chronic migraine and trigeminal neuralgia (Zhang et al. 2020; Cernuda-Morollón et al. 2015); decrease in serotonin concentrations when collected from human tears in refractory intractable DED (Choi et al. 2019), and increase in peripheral blood platelets in painful cervical dystonia associated to depression and anxiety (Karakulova and Loginova 2017); decrease in plasma concentration of markers of oxidative damage to proteins and increase in biomarkers for antioxidant power that measures non-enzymatic antioxidant properties and other components of plasma antioxidant barrier in chronic migraine (Dini et al. 2019); decrease in expression of gene sets involved in inflammatory pathways and immune cells classes in the periosteum of chronic migraineurs that responded well to past therapy (Gfrerer et al. 2022), and MMP-9 molecule in the tears samples of participants suffering from DED (Choi et al. 2019). Other studies also observed differences following BoNT, although it was not reported statistical significance, which included a decrease in salivary levels of CGRP after the third month in chronic migraine (National Library of Medicine (US). Identifier NCT01071096. 2023); CGRP and SP-positive nerve fibres were higher in the scalp areas previously with/without associated pain and alopecia and the nerve fibres increased density and thickness (Cutrer et al. 2010). Finally, there were apparently no mean changes in CGRP, beta Tubulin and SNAP-25 in the skin of patients suffering from chronic migraine (National Library of Medicine (US). Identifier NCT02037425. 2023).

Remarkably, while the statistical significance reported informed that an effect exists in six studies, the P value cannot reveal the size of the effect. Therefore, once the effect size was not reported, it was not possible to understand the magnitude of the differences found. We highlight that effect sizes should be reported consistently to complement significance tests for the interpretation of results and understand the full impact of BoNT in biomarkers of interest and relevant clinical effects. Moreover, the correct reporting of effect size will help to determine sample sizes for future studies, and to facilitate comparison between studies in meta-analyses. Overall, very low to moderate-certainty evidence was found for the BoNT key effects on biomarkers used in clinical research in chronic conditions linked to inflammation, which reflects the outcomes in the GRADE evaluation. That said, there are several observations in the included studies worthy of discussion and further research.

The two studies evaluating serotonin concentration (ng/mL) suggested that BoNT analgesic effect may be primarily related with a weakening of the muscle contraction that interrupts the vicious reflex cycle in refractory intractable DED and cervical dystonia with pain in the neck and associated with depression and anxiety (Karakulova and Loginova 2017; Choi et al. 2019). Nevertheless, both studies reported that BoNT had significant effect on serotonin levels in the tears and peripheral blood platelet. Noteworthy that serotonin is known to be involved in complex biological functions such as mood, cognition, learning, and physiological processes including vasoconstriction and wound healing. It is thought that in human tears, serotonin may act as a surrogate marker for corneal nociceptor sensitization, while there is evidence suggesting that platelets with serotonin content may be a reliable peripheral surrogate in neuropsychiatric research (Zhuang et al. 2018). Hence, the results from these studies may contribute to validate a biomarker in refractory intractable DED and non-motor symptoms associated to cervical dystonia, by demonstrating that the relationship between the change in serotonin and the change in clinical outcome is generalisable across interventions, including BoNT therapy.

Another interesting finding, based on differential gene expression analyses of treatment effect, was that BoNT demonstrated to have a large effect on the expression of inflammatory genes and pathways activation, as well as on the abundance of multiple inflammatory/immune cells, in the periosteum (Gfrerer et al. 2022). This study offered a new potential avenue for BoNT mechanism of action in preventing chronic migraine, set on the BoNT power to reduce pre-existing inflammation via localized interaction, and hence decrease the ample quantity of classic immune cells in the calvarium periosteum. Further studies providing larger sample sizes and a control group should focus on this novel possibility that BoNT may have anti-inflammatory properties driven by its capacity to modulate nociceptors release of neuropeptides and chemokines in highly innervated tissues. Reinforcing the potential role of BoNT in modulating inflammation, one RCT evaluated the tears level of chronic inflammation marker (MMP-9) in refractory intractable DED and observed significant outcome changes (Choi et al. 2019). Indeed, experimental research and ongoing clinical studies searching for an effective treatment of DED have targeted the inhibition of molecule MMP-9 (Shoari et al. 2021). Thus, this RCT offers a new treatment possibility and adds to the evidence required to validate MMP-9 biomarker in DED. However, the true effect of BoNT could not be ascertained due to some factors that may have affected the outcome. The effects of such variables, including age-related changes in the lid function, meibomian and lacrimal grands, the use of lubricants or drugs for other reasons (e.g., systemic immunomodulators) that may have contributed to the inhibition of MMP-9 activity, needs to be examined in future well-designed RCTs with representative samples.

In addition, it seems that BoNT treatment may ameliorate the functioning of antioxidant mechanisms in chronic migraineurs. One study confirmed that BoNT not only was a successful prophylactic therapy for chronic migraine, as it improved the concentration of plasma oxidative stress biomarkers (Dini et al. 2019). Considering the far-reaching implications of BoNT putative indirect antioxidant properties for the treatment of other conditions, it may be worth to clarify the role of BoNT in the reported treatment outcomes. Bearing in mind that biological pathways and therapeutic effects are multifactorial and not simple, future studies should define the weight of other variables of interest on the level of oxidative stress impairment (e.g., severity of chronic migraine and preventives overuse) and on the level of antioxidative activity reduction (e.g., concomitant use of triptans and/or nonsteroidal anti-inflammatory drugs consumption). Notwithstanding some important variables that have already been considered in some studies, with apparently no impact on the change in biomarker and/or clinical outcome. For example, analysis reported that age, gender, visual analog scale (VAS) scores, use of carbamazepine, and the number of trigeminal nerve branches involved had no significant influence on the median difference between plasma CGRP concentrations in trigeminal neuralgia (Zhang et al. 2020). Moreover, it was reported no numerical or statistical differences between clinical outcomes in chronic migraine and demographic factors such as age, clinical features including duration of condition, history of aura, and acute medication overuse, as well as comorbidities (Cernuda-Morollón et al. 2015). These included depression, fibromyalgia, arterial hypertension, and obesity. Other variables which effects were excluded in the context of chronic migraine involved the use of triptans, preventatives (monotherapy or polytherapy) and topiramate. In contrast, one study found an inverse relationship between self-reported pain severity and intensity in cervical dystonia and quantitative serotonin content in peripheral blood platelets (Karakulova and Loginova 2017), which should be confirmed in further randomised clinical trials measuring both self-reported outcome and biomarker.

In line with the current prevailing thought, two studies indicated that CGRP is likely to be involved in the pathophysiology of chronic migraine and trigeminal neuralgia and suggested that the analgesic mechanism of BoNT in the treatment of both conditions may be associated with the blockage of CGRP release, leading to the reversal of peripheral and central sensitization (Zhang et al. 2020; Cernuda-Morollón et al. 2015). However, while the two studies reported that CGRP plasma levels were significantly reduced after treatment with BoNT, the conclusions diverged regarding the value of CGRP concentration measurements before starting the therapy to predict the response to BoNT. Only the study involving chronic migraineurs reported that interictal CGRP plasma levels may be helpful in predicting the response to BoNT (Cernuda-Morollón et al. 2015), which was in line with the results from four studies evaluating plasma or serum levels of CGRP and other pain-producing molecules (see Online Resource 4Table 2). These observational studies were all in chronic migraine but were excluded from analysis given that biomarkers levels were only collected before BoNT administration (Cernuda-Morollón et al. 2014; Leira et al. 2021; Domínguez et al. 2018; Domínguez Vivero et al. 2020). Noteworthy another study included in Online Resource 4 Table 2, which provided a peripheral blood genomic DNA analysis of 156 participants with chronic migraine and observed that polymorphic variations of genes that encode for CGRP might play a role as prognostic markers of efficacy of BoNT (Moreno-Mayordomo et al. 2019). There is a need for results refinement and the authors were aware of one registered protocol (CRD42021265014) for a systematic review on biomarker predictors of BoNT efficacy in chronic migraine that may be able to further elucidate this matter (Sari 2021).

Moreover, given the limited clinical studies available in this field, it may be relevant for future research the scrutiny of the validity of preclinical evidence. The importance of knowing which biomarkers have been used, as well as BoNT key effect, in animal or cells studies has been suggested by the authors on another registered protocol (CRD42023432411) or highlighted in the results of the studies included in Online Resource 4 Table 3, respectively (Pereira 2023).

### Limitations

We acknowledge some limitations. The included studies were often limited by small sample sizes, and it was unclear if the follow-up time was adequate for the development of the studied chronic diseases. Moreover, the small number of studies and lack of direct comparisons prevented a meta-analysis for several outcomes and only narrative analyses were possible. One of the primary limitations was the heterogeneity of chronic conditions and biological sampling across studies, although this review restricted the use of BoNT to head and neck conditions. On the other hand, expanding the targeted areas and indications below the eligibility criteria (e.g., below head and neck plaque psoriasis, hypertrophic scars and keloids as reported in Online Resource 4 Table 4) may amplify the results. Moreover, the study design and reporting methods varied widely across studies, while in one sense the review was more inclusive (e.g., studies with/without controls have been included), allowing to have a good representation of the available evidence on this topic, baseline confounding is likely to be an issue in most or all non-randomised studies. Potential confounders or other sources of bias were also identified in the included studies. Most studies reported knowledge of the assigned intervention, with six out of nine studies reporting sources of funding, what could interfere with patient reported outcomes (such as level of pain) and investigator-reported outcomes requiring some judgement. Therefore, while such concerns do not apply to the main outcome (quantitative biomarker levels upon BoNT administration), it may apply to potential relationships between biomarker change and clinical subjective effect (measurement of bias). For example, some studies adopted different subgroups of responders versus non-responders to BoNT treatment, which was primarily based on subjective self-report and clinical measures. This was not considered to interfere with the primary outcome objective laboratory assessments, especially in studies with blinded outcome assessors (Cernuda-Morollón et al. 2015; Gfrerer et al. 2022), whereas it was judged as a moderate to serious risk of bias for the studies with open label design and outcome measurements involving assessor classification of the biomarker change on a scale, which may have introduced some bias in measurement of outcomes (Cutrer et al. 2010; National Library of Medicine (US). Identifier NCT02037425. 2023). Moreover, although based on histological examinations, the results for some outcomes were not comprehensively presented: no power calculation undertaken, no potential sources of bias identified, no statistical tests or *P* values reported.

## Conclusion

In the present review study, the evidence achieved from clinical studies in BoNT was summarised. A significant effect was reported in levels of CGRP, levels of serotonin, concentration of oxidative stress biomarkers, expression of gene sets involved in inflammatory pathways, immune cells classes and MMP-9 molecule. However, these findings are supported by low-quality data concerning BoNT effects measured with objective biomarkers. Further well‐designed trials in this area are needed to increase certainty about BoNT effects for chronic conditions linked to inflammation.

## Registration and protocol

This systematic review has been registered in the international prospective register of systematic reviews (PROSPERO) database under the registration number: CRD42023432131, and the protocol has been published (Pereira. 2023). Differences between protocol and review: (1) Inclusion of “systematic review” in the title to follow PRISMA 2020 guidelines. (2) The “risk of bias” methods was updated with the new tool ROB 2.0 in line with guidance from the new version of the Cochrane Handbook for Systematic Reviews of Interventions. (3) Given the small amount of RCTs and the inclusion of non-randomised studies to the evidence synthesis, we have updated the methods with the ROBIN-I. (4) We included the GRADE approach to rate the quality of evidence for each outcome. (5) We could not assess potential sources of effect heterogeneity as planned. (6) Meanwhile, the first author has been granted a doctoral scholarship.

## Supplementary Information

Below is the link to the electronic supplementary material.

Supplementary file1 (DOCX 36 KB)

Supplementary file2 (DOCX 22 KB)

Supplementary file3 (DOCX 20 KB)

Supplementary file4 (DOCX 44 KB)

Supplementary file5 (DOCX 29 KB)

Supplementary file6 (DOCX 29 KB)

Supplementary file7 (DOCX 52 KB)

Supplementary file8 (DOCX 68 KB)

## Data Availability

Full list of citations that did not meet the inclusion criteria was recorded in the Ryann platform and is available upon request. Justifications for risk of bias assessments and study quality evaluations were recorded in the CASP checklists, which are available upon request.
