# Supplementary material for: Botulinum toxin effects on biochemical biomarkers related to inflammation-associated head and neck chronic conditions: a systematic review of clinical research
Source: J Neural Transm (Vienna). 2025 Mar 4;132(12):1851–74. doi: 10.1007/s00702-024-02869-w (PMC12669376; doi:10.1007/s00702-024-02869-w)
Supplement: Supplementary file 2 — Supplementary file2 (DOCX 22 KB) [file 702_2024_2869_MOESM2_ESM.docx]

**Table 1 – Reviewer 1 (IP) search strategy**

Terms used in the research.

| **Database** | **Search Format** |
| --- | --- |
| PUBMED | (((neurotoxin) OR (neuromodulator)) AND ((inflammation) OR (inflammatory state) OR (chronic orofacial pain) OR (orofacial neuropathic pain) OR (chronic migraine) OR (chronic arthritis) OR (herpetic neuralgia) OR (trigeminal neuralgia) OR (temporomandibular joint pain) OR (head and neck myofascial pain) OR (facial hypertrophic scars) OR (facial keloids) OR (periodontitis) OR (mood disorders) OR (anxiety) OR (chronic stress) OR (depression) OR (rosacea) OR (psoriasis) OR (dermatitis) OR (cephalalgia) OR (alopecia)) AND ((neuroinflammation) OR (neurogenic inflammation) OR (neuropeptides) OR (substance P) OR (calcitonin gene relative peptide) OR (glutamate) OR (anti-inflammatories (pain mediators) OR (inflammation mediators) OR (cytokines) OR (chemokines) OR (interleukin – 6) OR (interleukin – 10) OR (interleukin – 1 beta) OR (tumor necrosis factor alfa) OR (tumor necrosis factor weak inducer of apoptosis) OR (C-reactive protein) OR (monocyte chemoattractant protein-1) OR (brain-derived neurotrophic factor) OR (nerve growth factor) OR (astroglia markers) OR (microglia markers) OR (glial fibrillary acidic protein) OR (5-hydroxytryptamine) OR (ionized calcium-binding adaptor molecule 1) OR (antioxidant stress) OR (reactive oxidative stress) OR (reactive oxygen species) OR (secondary messengers) OR (markers) OR (biomarkers))) AND (botulinum toxin)  *A)* ***Filters****: Full text, Case Reports, Clinical Study, Clinical Trial, Clinical Trial Protocol, Comparative Study, Controlled Clinical Trial, Meta-Analysis, Multicenter Study, Observational Study, Randomized Controlled Trial, Review, Systematic Review, Humans.* ***134 selected items*** Sent On: Fri Jun 23 10:01:36 2023  *B)* ***Filters****: Full text, Systematic Review, Humans.* ***10 selected items*** Sent On: Fri Jun 23 18:16:13 2023  C) ***Filters****: Full text, Review, Systematic Review, Humans.* ***87 selected items*** Sent On: Fri Jun 23 18:55:19 2023  D) **Filters**: Full text, Clinical Study, Clinical Trial, Clinical Trial, Phase I, Clinical Trial, Phase II, Clinical Trial, Phase III, Clinical Trial, Phase IV, Controlled Clinical Trial, Meta-Analysis, Observational Study, Randomized Controlled Trial, Systematic Review, Humans. **40 *selected items*** Sent On: Fri Jun 23 19:18:27 2023 |
| SCOPUS | Your query : (ALL("**botulinum toxin**") AND ALL("**chronic inflammation**") AND ( EXCLUDE ( DOCTYPE,"ch" ) OR EXCLUDE ( DOCTYPE,"bk" ) OR EXCLUDE ( DOCTYPE,"re" ) OR EXCLUDE ( DOCTYPE,"cp" ) OR EXCLUDE ( DOCTYPE,"ed" ) OR EXCLUDE ( DOCTYPE,"le" ) OR EXCLUDE ( DOCTYPE,"no" ) OR EXCLUDE ( DOCTYPE,"sh" ) OR EXCLUDE ( DOCTYPE,"er" ) ) AND ( **LIMIT-TO** ( EXACTKEYWORD,"**Humans**" ) OR LIMIT-TO ( EXACTKEYWORD,"Human" ) OR LIMIT-TO ( EXACTKEYWORD,"**Adult**" ) OR EXCLUDE ( EXACTKEYWORD,"Animals" ) OR **EXCLUDE** ( EXACTKEYWORD,"**Animal**" ) OR EXCLUDE ( EXACTKEYWORD,"**Aged**" ) OR EXCLUDE ( EXACTKEYWORD,"**Nonhuman**" ) OR LIMIT-TO ( EXACTKEYWORD,"Botulinum Toxin" ) OR EXCLUDE ( EXACTKEYWORD,"**Interstitial Cystitis**" ) OR EXCLUDE ( EXACTKEYWORD,"Child" ) OR EXCLUDE ( EXACTKEYWORD,"**Cystitis**" ) OR EXCLUDE ( EXACTKEYWORD,"**Bladder Biopsy**" ) OR EXCLUDE ( EXACTKEYWORD,"**Spinal Cord Injury**" ) OR EXCLUDE ( EXACTKEYWORD,"**Hyaluronic Acid**" ) OR EXCLUDE ( EXACTKEYWORD,"**Urothelium**" ) OR EXCLUDE ( EXACTKEYWORD,"**Urodynamics**" ) OR EXCLUDE ( EXACTKEYWORD,"**Neurogenic Bladder**" ) OR EXCLUDE ( EXACTKEYWORD,"**Shoulder Pain**" ) OR EXCLUDE ( EXACTKEYWORD,"**Urinary Bladder, Overactive**" ) OR EXCLUDE ( EXACTKEYWORD,"**Uvomorulin**" ) OR EXCLUDE ( EXACTKEYWORD,"**Vulvodynia**" ) ) ) Number of results : **60** On: Fri Jun 30 18:16:13 2023 |
| WEB OF SCIENCE | (ALL=(**botulinum toxin**)) AND ALL=(**chronic**) and 1.142 Urology or 1.82 Gait & Posture or 1.95 Gastrointestinal & Esophageal Diseases or 1.43 Anesthesiology or 1.129 Back Pain or 1.128 Fertility, Endometriosis & Hysterectomy or 1.37 Cardiology - General or 1.134 Trauma & Emergency Surgery or 1.44 Nutrition & Dietetics or 1.105 Strokes or 1.137 Sleep Science & Circadian Systems or 1.151 Pancreas & Gall Bladder Disorders or 3.91 Contamination & Phytoremediation or 1.112 Palliative Care or 1.130 Lymphomas or 1.163 Parasitology - General or 1.172 Sports Science or 1.194 Tuberculosis & Leprosy or 1.199 Lung Cancer or 1.233 Pelvic & Renal Disorders or 1.243 Kidney Diseases or 1.23 Antibiotics & Antimicrobials or 1.65 Allergy or 1.150 Hearing Loss or 1.195 Neuroendocrine & Intestinal Disorders or 1.173 Cosmetic Surgery or 1.216 Abdominal Surgery or 1.168 Vascular, Cardiac & Thoracic Surgery or 3.232 Veterinary Sciences or 3.60 Herbicides, Pesticides & Ground Poisoning or 3.2 Marine Biology or 1.94 Cardiac Arrhythmia or 1.315 Laser Surgery, Therapy & Protection or 1.14 Nursing or 1.120 Inflammatory Bowel Diseases & Infections or 1.81 Reproductive Biology or 1.273 Health Literacy & Telemedicine or 1.313 History Of Medicine or 1.324 Bacterial Toxins & Diseases or 2.176 Drug Delivery Chemistry or 2.211 Mass Spectrometry or 3.16 Phytochemicals or 3.198 Mycotoxins or 3.45 Soil Science or 3.51 Dairy & Animal Sciences or 3.83 Bioengineering or 1.68 Lipids or 4.289 Biophotonics & Electromagnetic Field Safety (**Exclude – Citation Topics Meso**) and 1.247.1001 Dystonia or 1.34.982 Achilles Tendon or 1.247.2375 Hyperhidrosis or 1.7.661 Saccades or 1.253.1927 Compartment Syndrome or 1.157.1089 Lung Transplantation or 1.21.1604 Electroconvulsive Therapy or 1.218.658 Carotid Body or 1.235.1679 Foreign Body or 1.34.1657 Elbow or 1.34.158 Total Arthroplasty or 1.281.1192 Gout or 1.36.2358 Retrobulbar Anesthesia or 1.5.894 Nicotine or 1.5.1674 Ketogenic Diet (**Exclude – Citation Topics Micro**) and 1.235.1617 Deglutition (**Exclude – Citation Topics Micro**) and Letter or Note or Editorial Material or Meeting Abstract or Retracted Publication or News Item or Correction or Book Chapters (**Exclude – Document Types**) and Obstetrics Gynecology or Respiratory System or Language Linguistics or Nursing or Nutrition Dietetics or Telecommunications or Reproductive Biology or Health Policy Services or Urology Nephrology or Sport Sciences or Pediatrics (**Exclude – Web of Science Categories**) and Proceeding Paper (**Exclude – Document Types**) and 1.21.952 Obsessive-compulsive Disorder or 1.158.2035 Hidradenitis Suppurativa or 1.34.485 Shoulder or 1.34.440 Anterior Cruciate Ligament or 1.34.1020 Scaphoid or 1.265.939 Allergic Contact Dermatitis or 1.253.1100 Carpal Tunnel Syndrome or 1.222.542 Epilepsy Surgery (**Exclude – Citation Topics Micro**) and CURRENT SLEEP MEDICINE REPORTS or GASTROENTEROLOGY or ANNALES DE CHIRURGIE PLASTIQUE ESTHETIQUE or INTERNATIONAL JOURNAL OF LOWER EXTREMITY WOUNDS (**Exclude – Publication Titles**) and 1.222.2122 Conversion Disorder or 1.203.1500 Guillain-barre Syndrome (**Exclude – Citation Topics Micro**). **634** ***selected items (421 articles; 213 review articles)*** Sent On: Wed Jul 19 12:23:35 2023 |
| **Website/Register** | **Search Format** |
| Cinicaltrials.gov | Intervention/treatment = (“**Botulinum toxin**”)  Condition/disease = (“**chronic**”) Number of results : **172** On: Sun Jul 02 17:17:24 2023  Intervention/treatment = (“**Botulinum toxin**”)  Condition/disease = (“**temporomandibular disorders**”) (“**pain**”) Number of results : **10** On: Fri Jul 14 11:49:20 2023  Intervention/treatment = (“**Botulinum toxin**”)  Condition/disease = (“**trigeminal neuralgia**”) Number of results : **5** On: Fri Jul 14 11:19:26 2023  Intervention/treatment = (“**Botulinum toxin**”)  Condition/disease = (“**hypertrophic scars**”) (“**oral face**”) Number of results : **1** On: Fri Jul 14 12:17:00 2023  Intervention/treatment = (“**Botulinum toxin**”)  Condition/disease = (“**mood disorders**”) (“**anxiety**”) (“**depression**”) (“**chronic stress**”) Number of results : **7** On: Fri Jul 14 12:26:09 2023  Intervention/treatment = (“**Botulinum toxin**”)  Condition/disease = (“**rosacea**”) (“**dermatitis**”) (“**psoriasis**”) Number of results : **4** On: Fri Jul 14 23:11:08 2023  Intervention/treatment = (“**Botulinum toxin**”)  Condition/disease = (“**alopecia**”) Number of results : **5** On: Fri Jul 14 23:59:43 2023 |
| PROSPERO | **508** records found for **botulinum toxin NOT Animal:DB** On: Fri Jul 07 18:47:18 2023 |

**Table 2 – Reviewer 2 (SD) search strategy**

Terms used in the research.

| **Database** | **Search Format** |
| --- | --- |
| PUBMED | (((neurotoxin) OR (neuromodulator)) AND ((inflammation) OR (inflammatory state) OR (chronic orofacial pain) OR (orofacial neuropathic pain) OR (chronic migraine) OR (chronic arthritis) OR (herpetic neuralgia) OR (trigeminal neuralgia) OR (temporomandibular joint pain) OR (head and neck myofascial pain) OR (facial hypertrophic scars) OR (facial keloids) OR (periodontitis) OR (mood disorders) OR (anxiety) OR (chronic stress) OR (depression) OR (rosacea) OR (psoriasis) OR (dermatitis) OR (cephalalgia) OR (alopecia)) AND ((neuroinflammation) OR (neurogenic inflammation) OR (neuropeptides) OR (substance P) OR (calcitonin gene relative peptide) OR (glutamate) OR (anti-inflammatories (pain mediators) OR (inflammation mediators) OR (cytokines) OR (chemokines) OR (interleukin – 6) OR (interleukin – 10) OR (interleukin – 1 beta) OR (tumor necrosis factor alfa) OR (tumor necrosis factor weak inducer of apoptosis) OR (C-reactive protein) OR (monocyte chemoattractant protein-1) OR (brain-derived neurotrophic factor) OR (nerve growth factor) OR (astroglia markers) OR (microglia markers) OR (glial fibrillary acidic protein) OR (5-hydroxytryptamine) OR (ionized calcium-binding adaptor molecule 1) OR (antioxidant stress) OR (reactive oxidative stress) OR (reactive oxygen species) OR (secondary messengers) OR (markers) OR (biomarkers))) AND (botulinum toxin)  **Filters**: Full text, Clinical Study, Clinical Trial, Meta-Analysis, Randomized Controlled Trial, Systematic Review, Humans. **36 *selected items*** Sent On: Thu Sep 28 13:27 2023 |
| SCOPUS | KEY ( botulinum  AND toxin  AND  chronic  AND inflammation )  AND  ( LIMIT-TO ( DOCTYPE ,  "ar" )  OR  LIMIT-TO ( DOCTYPE ,  "re" ) )  AND  ( LIMIT TO ( EXACTKEYWORD ,  "Human" )  OR  LIMIT-TO ( EXACTKEYWORD ,  "Botulinum Toxin A" )  OR  LIMIT-TO ( EXACTKEYWORD ,  "Humans" )  OR  LIMIT-TO ( EXACTKEYWORD ,  "Article" )  OR  LIMIT-TO ( EXACTKEYWORD ,  "Review" )  OR  LIMIT-TO ( EXACTKEYWORD ,  "Inflammation" )  OR  LIMIT-TO ( EXACTKEYWORD ,  "Botulinum Toxin" )  OR  LIMIT-TO ( EXACTKEYWORD ,  "Male" )  OR  LIMIT-TO ( EXACTKEYWORD ,  "Adult" )  OR  LIMIT-TO ( EXACTKEYWORD ,  "Botulinum Toxins, Type A" )  OR  LIMIT-TO ( EXACTKEYWORD ,  "Pain" )  OR  LIMIT-TO ( EXACTKEYWORD ,  "Treatment Outcome" )  OR  LIMIT-TO ( EXACTKEYWORD ,  "Drug Efficacy" )  OR  LIMIT-TO ( EXACTKEYWORD ,  "Chronic Disease" )  OR  LIMIT-TO ( EXACTKEYWORD ,  "Chronic Inflammation" )  OR  LIMIT-TO ( EXACTKEYWORD ,  "Clinical Article" )  OR  LIMIT-TO ( EXACTKEYWORD ,  "Chronic Pain" )  OR  LIMIT-TO ( EXACTKEYWORD ,  "Migraine" )  OR  LIMIT-TO ( EXACTKEYWORD ,  "Headache" )  OR  LIMIT-TO ( EXACTKEYWORD ,  "Protein Expression" )  OR  LIMIT-TO ( EXACTKEYWORD ,  "Analgesia" )  OR  LIMIT-TO ( EXACTKEYWORD ,  "Drug Effect" )  OR  LIMIT-TO ( EXACTKEYWORD ,  "Neurogenic Inflammation" )  OR  LIMIT-TO ( EXACTKEYWORD ,  "Neuropathic Pain" )  OR  LIMIT-TO ( EXACTKEYWORD ,  "Calcitonin Gene Related Peptide" )  OR  LIMIT-TO ( EXACTKEYWORD ,  "Case Report" )  OR  LIMIT-TO ( EXACTKEYWORD ,  "Female" )  OR  LIMIT-TO ( EXACTKEYWORD ,  "Clinical Trial" )  OR  LIMIT-TO ( EXACTKEYWORD ,  "Botox" )  OR  LIMIT-TO ( EXACTKEYWORD ,  "Botulinum Toxins" )  OR  LIMIT-TO ( EXACTKEYWORD ,  "Tumor Necrosis Factor" )  OR  LIMIT-TO ( EXACTKEYWORD ,  "Interleukin 6" )  OR  LIMIT TO ( EXACTKEYWORD ,  "Depression" )  OR  LIMIT-TO ( EXACTKEYWORD ,  "Antiinflammatory Activity" )  OR  LIMIT-TO ( EXACTKEYWORD ,  "Gene Expression" )  OR  LIMIT-TO ( EXACTKEYWORD ,  "Interleukin 1beta" )  OR  LIMIT-TO ( EXACTKEYWORD ,  "Substance P" )  OR  LIMIT-TO ( EXACTKEYWORD ,  "Cytokine" )  OR  LIMIT-TO ( EXACTKEYWORD ,  "Mast Cell" )  OR  LIMIT-TO ( EXACTKEYWORD ,  "Neuralgia" )  OR  LIMIT-TO ( EXACTKEYWORD ,  "Neuromodulation" )  OR  LIMIT-TO ( EXACTKEYWORD ,  "Immune Response" )  OR  LIMIT-TO ( EXACTKEYWORD ,  "Nervous System Inflammation" )  OR  LIMIT-TO ( EXACTKEYWORD ,  "Systematic Review" )  OR  LIMIT-TO ( EXACTKEYWORD ,  "Biological Marker" )  OR  LIMIT-TO ( EXACTKEYWORD ,  "Immunology" )  OR  LIMIT-TO ( EXACTKEYWORD ,  "Osteoarthritis" )  OR  LIMIT-TO ( EXACTKEYWORD ,  "Multiple Sclerosis" )  OR  LIMIT-TO ( EXACTKEYWORD ,  "Synaptosomal Associated Protein 25" )  OR  LIMIT-TO ( EXACTKEYWORD ,  "Botulinum Toxin Type A" )  OR  EXCLUDE ( EXACTKEYWORD ,  "Nonhuman" )  OR  EXCLUDE ( EXACTKEYWORD ,  "Priority Journal" )  OR  EXCLUDE ( EXACTKEYWORD ,  "Pathophysiology" )  OR  EXCLUDE ( EXACTKEYWORD ,  "Quality Of Life" )  OR  EXCLUDE ( EXACTKEYWORD ,  "Amitriptyline" )  OR  EXCLUDE ( EXACTKEYWORD ,  "Corticosteroid" )  OR  EXCLUDE ( EXACTKEYWORD ,  "Lidocaine" )  OR  EXCLUDE ( EXACTKEYWORD ,  "Neuromuscular Agents" )  OR  EXCLUDE ( EXACTKEYWORD ,  "Nuclear Magnetic Resonance Imaging" )  OR  EXCLUDE ( EXACTKEYWORD ,  "Pathology" )  OR  EXCLUDE ( EXACTKEYWORD ,  "Prevalence" )  OR  EXCLUDE ( EXACTKEYWORD ,  "Gabapentin" )  OR  EXCLUDE ( EXACTKEYWORD ,  "Hyaluronic Acid" )  OR  EXCLUDE ( EXACTKEYWORD ,  "Pathogenesis" )  OR  EXCLUDE ( EXACTKEYWORD ,  "Antibiotic Agent" )  OR  EXCLUDE ( EXACTKEYWORD ,  "Human Tissue" )  OR  EXCLUDE ( EXACTKEYWORD ,  "Interstitial Cystitis" )  OR  EXCLUDE ( EXACTKEYWORD ,  "Physiotherapy" )  OR  EXCLUDE ( EXACTKEYWORD ,  "Nonsteroid Antiinflammatory Agent" )  OR  EXCLUDE ( EXACTKEYWORD ,  "Unclassified Drug" )  OR  EXCLUDE ( EXACTKEYWORD ,  "Cystoscopy" )  OR  EXCLUDE ( EXACTKEYWORD ,  "Metabolism" )  OR  EXCLUDE ( EXACTKEYWORD ,  "Muscle Relaxant Agent" )  OR  EXCLUDE ( EXACTKEYWORD ,  "Differential Diagnosis" )  OR  EXCLUDE ( EXACTKEYWORD ,  "Visual Analog Scale" )  OR  EXCLUDE ( EXACTKEYWORD ,  "Animals" )  OR  EXCLUDE ( EXACTKEYWORD ,  "Computer Assisted Tomography" )  OR  EXCLUDE ( EXACTKEYWORD ,  "Nausea" )  OR  EXCLUDE ( EXACTKEYWORD ,  "Transformed Migraine" )  OR  EXCLUDE ( EXACTKEYWORD ,  "Urinary Tract Infection" )  OR  EXCLUDE ( EXACTKEYWORD ,  "Urodynamics" )  OR  EXCLUDE ( EXACTKEYWORD ,  "Animal" )  OR  EXCLUDE ( EXACTKEYWORD ,  "Chondroitin Sulfate" )  OR  EXCLUDE ( EXACTKEYWORD ,  "Cystalgia" )  OR  EXCLUDE ( EXACTKEYWORD ,  "Dimethyl Sulfoxide" )  OR  EXCLUDE ( EXACTKEYWORD ,  "Flu Like Syndrome" )  OR  EXCLUDE ( EXACTKEYWORD ,  "Pentosan Polysulfate" )  OR  EXCLUDE ( EXACTKEYWORD ,  "Clindamycin" )  OR  EXCLUDE ( EXACTKEYWORD ,  "Cystitis" )  OR  EXCLUDE ( EXACTKEYWORD ,  "Disease Severity" )  OR  EXCLUDE ( EXACTKEYWORD ,  "Drug Withdrawal" )  OR  EXCLUDE ( EXACTKEYWORD ,  "Dysphagia" )  OR  EXCLUDE ( EXACTKEYWORD ,  "Lower Urinary Tract Symptom" )  OR  EXCLUDE ( EXACTKEYWORD ,  "Muscle Spasm" )  OR  EXCLUDE ( EXACTKEYWORD ,  "Muscle Weakness" )  OR  EXCLUDE ( EXACTKEYWORD ,  "Pelvis Pain Syndrome" )  OR  EXCLUDE ( EXACTKEYWORD ,  "Physical Examination" )  OR  EXCLUDE ( EXACTKEYWORD ,  "Tacrolimus" )  OR  EXCLUDE ( EXACTKEYWORD ,  "Cystitis, Interstitial" )  OR  EXCLUDE ( EXACTKEYWORD ,  "Child" )  OR  EXCLUDE ( EXACTKEYWORD ,  "Fatigue" )  OR  EXCLUDE ( EXACTKEYWORD ,  "Overactive Bladder" )  OR  EXCLUDE ( EXACTKEYWORD ,  "Urinary Frequency" )  OR  EXCLUDE ( EXACTKEYWORD ,  "Urinary Urgency" )  OR  EXCLUDE ( EXACTKEYWORD ,  "Bladder Biopsy" )  OR  EXCLUDE ( EXACTKEYWORD ,  "Bladder" )  OR  EXCLUDE ( EXACTKEYWORD ,  "Bladder Pain Syndrome" )  OR  EXCLUDE ( EXACTKEYWORD ,  "Dizziness" )  OR  EXCLUDE ( EXACTKEYWORD ,  "Cystectomy" )  OR  EXCLUDE ( EXACTKEYWORD ,  "Doxycycline" ) )  **Parte superior do formulário**  **10** document results Sent On: Thu Sep 28 19:31 2023Parte inferior do formulário |
| WEB OF SCIENCE | (**botulinum toxin for chronic inflammation** (All Fields) **119 *selected items (76 articles; 40 review articles)*** Sent On: Fri Sep 29 10:16:38 2023 |
| **Website/Register** | **Search Format** |
| Cinicaltrials.gov | Intervention/treatment = (“**Botulinum toxin type A**”)  Condition/disease = (“**chronic pain**”) Number of results : **54** On: Fri Sep 29 12:48:14 2023  Intervention/treatment = (“**Botulinum toxin**”)  Condition/disease = (“**chronic disorders**”) Number of results : **159** On: Fri Sep 29 12:52:30 2023  Intervention/treatment = (“**Botulinum toxin**”)  Condition/disease = (“**chronic disorders**”) (“**head and neck**”) Number of results : **18** On: Fri Sep 29 12:54:23 2023 |
| PROSPERO | **33** records found for **botulinum toxin AND (blood_and_immune_system OR Ccare_of_the_elderly OR dental OR ear_nose_and_throat OR eye_disorders OR Mental health and behavioural conditions OR Neurological OR Oral health):HA**  On: Fri Sep 29 13:47:18 2023 |
