# Supplementary material for: Botulinum toxin effects on biochemical biomarkers related to inflammation-associated head and neck chronic conditions: a systematic review of clinical research
Source: J Neural Transm (Vienna). 2025 Mar 4;132(12):1851–74. doi: 10.1007/s00702-024-02869-w (PMC12669376; doi:10.1007/s00702-024-02869-w)
Supplement: Supplementary file 3 — Supplementary file3 (DOCX 20 KB) [file 702_2024_2869_MOESM3_ESM.docx]

**Ryyan REPORT**

Possible Duplicates: Unresolved **0**; Deleted **235**; Not duplicates **9**; Resolved **52**

Auto Deduplicated: 2 exact matches **7**; 3 exact matches **27**; 4 exact matches **6**

**(2-1)x7 + (3-1)x27 + (4-1)x6 = 7 + 54 + 18 = 79**

Inclusion decisions: Undecided **0**; Maybe **16**; Included **6**; Excluded **802**

Keywords for include: randomized **66**; placebo **53**; trial **51**; double blind **37**; placebo controlled **33**; CGRP **27**; compared with **16**; controlled trial **15**; randomized controlled trial **10**; randomised **9**; controlled study **7**; randomly **5**; randomly assigned **3**; parallel group **3**; assigned to **3**; biomarker **3**; randomised controlled trial **1**; randomly allocated **1**; controlled design **1**; double blinded **1**; cross over **1**; crossover **1**; RCT **1**; parallel groups **0**; control groups **0**; double marked **0**; doubleblinded **0**; single masked **0**; single blind **0**; doubleblind **0**; CCT **0**

Keywords for exclude: systematic review **42**; meta-analysis **28**; this review **19**; reviews **13**; animal **12**; children **10**; literature review **9**; pediatric **6**; in vitro **6**; mouse **4**; mice **4**; rats **4**; rat **4**; rodents **1**; equine **1**; sensitivity and specificity **0**; regression analyses **0**; healthy control **0**; non-randomised **0**; healthy human **0**; nonrandomised **0**; nonrandomized **0**; case control **0**; age-matched **0**; transgenic **0**; cadaveric **0**; broilers **0**; cadavers **0**; purebred **0**; animals **0**; beagles **0**; broiler **0**; cadaver **0**; piglets **0**; porcine **0**; rabbits **0**; beagle **0**; canine **0**; murine **0**; rabbit **0**; rodent **0**; fish **0**; soil **0**

Search methods:

Uploaded References [pubmed-neurotoxin-set.txt] **10**

Uploaded References [pubmed-neurotoxin-set(1).txt] **40**

Uploaded References [pubmed2.txt] **87**

Uploaded References [pubmedSR1.(5).txt] **134**

Uploaded References [scopus(1).csv] **60**

Uploaded References [wos1.txt] **634**

Uploaded References [pubmedSR2.(5).txt] **36**

Uploaded References [scopus(1).csv] **18**

Uploaded References [wos1.txt] **119**

Labels: not head and neck **82**; not chronic inflammatory condition **28**; animal study **23**; combined therapy **15**; chemodenervation of glands **12**; not biomarkers **12**; focus on muscle activity **8**; not adults **8**; chronic condition with inflammatory component? **6**; biomarkers only collected before BoNTA treatment. Biomarker tested as predictor for favourable response **5**; in vitro study? **5**; chronic pain? cervical dystonia (movement disorder) **4**; chronic? **2**; vital signs - evaluating treatment safety **1**; healthy human **1**; to be confirmed if chronic inflammatory condition **1**; skin blood perfusion?biomarker **1**; primary outcome -data missing **1**

Exclusion reasons: wrong outcome **387**; wrong study design **363**; wrong drug **284**; wrong population **228**; other assessment rather than biomarker **150**; background article **26**; wrong publication type **2**

Topics: Botulinum Toxins, Type A, Botulinum Toxins, Migraine Disorders, Humans, Female, Male, Treatment Outcome, Adult, Middle Aged, Tension-Type Headache, Headache Disorders, Botulinum Toxins, Type A/*therapeutic use, Aged, Neuralgia, Prospective Studies, Animals, Temporomandibular Joint Disorders, Neurotoxins, Double-Blind Method, Botulinum Toxins, Type A/*administration & dosage, Humanism, Humanities, Chronic Disease, Botulinum Toxins, Type A/therapeutic use, Quality of Life, Pilot Projects, Young Adult, Calcitonin Gene-Related Peptide, Neuromuscular Agents/*therapeutic use*Botulinum Toxins, Type A/therapeutic use

Locations: USA, Italy, London, Boston, CAMA, Irvine, Taiwan, Denmark, Rome, UK, California, Hualien, Germany, AZ, Spain, Phoenix, Washington, Buddhist Tzu Chi General Hospital, San Francisco, Canada, Baltimore, Philadelphia, Chicago, Massachusetts, South Los Angeles, France, Austria, Brazil

Main language: eng **155**; ger **4**; fre **1**; jpn **1**; spa **1**; rus **1**; eng,spa **1**

Publication types: Journal Article **820**; Review **79**; Research Support, Non-U.S. Gov't **36**; Randomized Controlled Trial **28**; Clinical Trial **23**; Systematic Review **20**; Comparative Study **15**; Meta-Analysis **12**; Case Reports **10**; Multicenter Study **9**; Controlled Clinical Trial **7**; Observational Study **6**; Research Support, N.I.H., Extramural **5**; Clinical Trial, Phase III **4**; English Abstract **3**; Letter **3**; Clinical Trial, Phase II **1**; Research Support, U.S. Gov't, Non-P.H.S. **1**; Congress **1**; Clinical Trial, Phase IV **1**; Consensus Development Conference **1**

Journal: HEADACHE **55**; TOXINS **24**; JOURNAL OF HEADACHE AND PAIN **23**; CEPHALALGIA **20**; REVISTA DE NEUROLOGIA **18**; Toxins **14**; CURRENT PAIN AND HEADACHE REPORTS **12**; Cephalalgia : an international journal of headache **7**; Headache **7**; CURRENT TREATMENT OPTIONS IN NEUROLOGY **7**; INTERNATIONAL JOURNAL OF ORAL AND MAXILLOFACIAL SURGERY **6**; EXPERT REVIEW OF NEUROTHERAPEUTICS **6**; PLASTIC AND RECONSTRUCTIVE SURGERY **6**; SCHMERZ **6**; AKTUELLE NEUROLOGIE **5**; EUROPEAN JOURNAL OF NEUROLOGY **5**; NERVENARZT **5**; DRUGS & AGING **5**; LARYNGOSCOPE **5**; Neurological sciences: official journal of the Italian Neurological Society and of the Italian Society of Clinical Neurophysiology **5**; Pain **4**; ARQUIVOS DE NEURO-PSIQUIATRIA **4**; CURRENT NEUROLOGY AND NEUROSCIENCE REPORTS **4**; JOURNAL OF COSMETIC DERMATOLOGY **4**; JOURNAL OF PAIN RESEARCH **4**; JOURNAL OF ORAL AND MAXILLOFACIAL SURGERY **4**; PAIN AND THERAPY **4**; CNS DRUGS **4**; BRITISH JOURNAL OF ORAL & MAXILLOFACIAL SURGERY **4**; NEUROLOGICAL SCIENCES **4**; CANADIAN JOURNAL OF NEUROLOGICAL SCIENCES **4**; CLINICAL NEUROLOGY AND NEUROSURGERY **4**; Dermatologic surgery : official publication for American Society for Dermatologic Surgery [et al.] **4**; EXPERT OPINION ON PHARMACOTHERAPY **4**; COCHRANE DATABASE OF SYSTEMATIC REVIEWS **4**; CRANIO-THE JOURNAL OF CRANIOMANDIBULAR & SLEEP PRACTICE **4**; INTERNATIONAL JOURNAL OF MOLECULAR SCIENCES **4**; NERVENHEILKUNDE **3**; FRONTIERS IN PHARMACOLOGY **3**; JOURNAL OF CRANIOFACIAL SURGERY **3**; PAIN **3**; ACTA NEUROLOGICA BELGICA **3**; PAIN MEDICINE **3**; Current pharmaceutical design **3**; NEUROLOGY ASIA **3**; FRONTIERS IN NEUROLOGY **3**; CUREUS JOURNAL OF MEDICAL SCIENCE **3**; Current pain and headache reports **3**; SEMINARS IN NEUROLOGY **3**; JOURNAL OF VOICE **3**; FORTSCHRITTE DER NEUROLOGIE PSYCHIATRIE **3**; Neurology **3**; JOURNAL OF NEURAL TRANSMISSION **3**; NOROPSIKIYATRI ARSIVI-ARCHIVES OF NEUROPSYCHIATRY **3**; ANNALS OF OTOLOGY RHINOLOGY AND LARYNGOLOGY **3**;The Cochrane database of systematic reviews **3**; TOXICON **3**; JOURNAL OF THE NEUROLOGICAL SCIENCES **3**; SPRINGERPLUS **3**; European urology **2**; BRITISH JOURNAL OF PHARMACOLOGY **2**; MILITARY MEDICINE **2**; PAIN PRACTICE **2**; The Clinical journal of pain **2**; INTERNATIONAL JOURNAL OF NEUROSCIENCE **2**; NATURE REVIEWS NEUROLOGY **2**; EUROPEAN REVIEW FOR MEDICAL AND PHARMACOLOGICAL SCIENCES **2**; Journal of the American Academy of Dermatology **2**; ANNALS OF PLASTIC SURGERY **2**;PARKINSONISM & RELATED DISORDERS **2**; Expert opinion on emerging drugs **2**; JOURNAL OF ORAL SCIENCE **2**; PROGRESS IN NEUROBIOLOGY **2**; JOURNAL OF CLINICAL MEDICINE **2**; CLINICAL COSMETIC AND INVESTIGATIONAL DERMATOLOGY **2**; HEALTH PSYCHOLOGY RESEARCH **2**; JOURNAL OF DRUGS IN DERMATOLOGY **2**; NEUROLOGIA I NEUROCHIRURGIA POLSKA **2**; The Journal of clinical psychiatry **2**; HEADACHE QUARTERLY-CURRENT TREATMENT AND RESEARCH **2**; NEUROTHERAPEUTICS **2**; Urology **2**; CURRENT OPINION IN NEUROLOGY **2**; PLASTIC AND RECONSTRUCTIVE SURGERY-GLOBAL OPEN **2**; ACTA NEUROLOGICA SCANDINAVICA **2**; PLOS ONE **2**; Pharmacological research **2**; LARYNGOSCOPE INVESTIGATIVE OTOLARYNGOLOGY **2**; JOURNAL OF ORAL REHABILITATION **2**; JOURNAL OF NEUROSCIENCE RESEARCH **2**; ORAL AND MAXILLOFACIAL SURGERY CLINICS OF NORTH AMERICA **2**; JOURNAL OF PHARMACOLOGY AND EXPERIMENTAL THERAPEUTICS  **2**; BMJ clinical evidence **2**; JOURNAL OF ORAL & FACIAL PAIN AND HEADACHE **2**; THERAPEUTIC ADVANCES IN NEUROLOGICAL DISORDERS **2**;CURRENT MEDICAL RESEARCH AND OPINION **2**; LARYNGO-RHINO-OTOLOGIE **2**; OTOLARYNGOLOGY-HEAD AND NECK SURGERY **2**; JOURNAL OF CRANIO-MAXILLOFACIAL SURGERY **2**; EUROPEAN JOURNAL OF PHYSICAL AND REHABILITATION MEDICINE **2**

Authors: Dodick, DW **20**; Silberstein, SD **20**; Diener, HC **18**; Dodick, David W. **12**; Lipton, RB **11**; Silberstein, Stephen D. **11**; Brin, MF **11**; Blumenfeld, AM **10**; Martelletti, P **10**; DeGryse, RE **10**; Aurora, SK **9**; Diener, Hans-Christoph **9**; Lipton, Richard B. **9**; Mathew, NT 9; Straube, A **9**; Blumenfeld, Andrew M. **8**; Turkel, CC **8**; Kaye, AD **8**; Grazzi, L **7**; Kuo HC **7**; Brin, Mitchell F. **7**; Martelletti, Paolo **7**; Aurora, Sheena K. **7**; Kaye, Alan D. **7**; Tassorelli, C **7**; Evers, S **7**; Mathew, Ninan T. **7**; Ahmed, F **7**; Blumenfeld, A **7**; Irimia, P **6**; Dodick DW **6**; DeGryse, Ronald E. **6**; Schoenen, J **6**;Tassorelli, Cristina **6**; DeGryse, R **6**; Gaul, C **6**; Ahmed, Fayyaz **6**; Adams, AM **5**; Grazzi, Licia **5**; Gobel, H **5**; Viswanath, Omar **5**; Viswanath, O **5**; Guerrero, AL **5**; Elkind, AH **5**; Straube, A. **5**; May, A **5**; Irimia, Pablo **5**; Pascual, J **5**; Pollmann, W **5**; Feneberg, W **5**; Arendt-Nielsen L **5**; Schoenen, Jean **5**; Pozo-Rosich, P **5**; Jost, WH **5**; Silberstein SD **5**; Goadsby PJ **5**; Cady, R **4**; Martinez-Pias, Enrique **4**; Gawel, M **4**; Sommer, K **4**; Henze, T **4**; Guerrero, Angel L. **4**; Garcia-Azorin, David **4**; Obermann, M **4**; Turkel, Catherine C. **4**; Urits, I **4**; Gazerani P **4**; Pozo-Rosich, Patricia **4**; Pascual, Julio **4**; Schwartz, M **4**; Atchison, WD **4**; Urits, Ivan **4**; Sacco, S **4**; Guntinas-Lichius, O **4**; Steinbrecher, A **4**; Sierra, Alvaro **4**; Costa, J **4**; Sommer, Katherine **4**; Bendtsen, L **4**; Garcia-Azorin, D **4**; Gaul, Charly **4**; Martinez-Pias, E **4**; Sierra, A **4**; Straube, Andreas **4**; Sacco, Simona **4**; Turkel, C **4**; Christie, SN **3**; VanDenburgh, Amanda M. **3**; Blumenfeld, Andrew **3**; Diener, H. C. **3**; Vecsei, L **3**; Matharu, Manjit **3**; Cohen JL **3**; Delussi, M **3**; Janis, JE **3**; Ailani, J **3**; Negro, A **3**; VanDenburgh, AM 3; Goadsby, Peter J. **3**; Smania, N **3**

Year: 2023 **42**; 2022 **71**; 2021 **68**; 2020 **64**; 2019 **68**; 2018 **35**; 2017 **54**; 2016 **30**; 2015 **51**; 2014 **32**; 2013 **33**; 2012 **32**; 2011 **27**; 2010 **29**; 2009 **25**; 2008 **33**; 2007 **17**; 2006 **24**; 2005 **16**; 2004 **18**; 2003 **17**; 2002 **14**; 2001 **6**; 2000 **4**; 1999 **6**; 1998 **1**; 1997 **2**; 1995 **1**; 1994 **1**; 1993 **1**; 1992 **1**; 1976 **1**
