# Supplementary material for: Botulinum toxin effects on biochemical biomarkers related to inflammation-associated head and neck chronic conditions: a systematic review of clinical research
Source: J Neural Transm (Vienna). 2025 Mar 4;132(12):1851–74. doi: 10.1007/s00702-024-02869-w (PMC12669376; doi:10.1007/s00702-024-02869-w)
Supplement: Supplementary file 4 — Supplementary file4 (DOCX 44 KB) [file 702_2024_2869_MOESM4_ESM.docx]

**Supplementary Information 4 Table 1:** Biomarkers in Clinical Research on Botulinum Toxin effects on Chronic Inflammatory State. Listed reasons for inclusion versus exclusion studies requiring adjudication regarding eligibility

**REPORT**: From the reports assessed for eligibility, we excluded from our review 32 records requiring adjudication (33 - 64), and we listed the reasons for exclusion in the **appendix** **4** tables. Of these, we excluded six studies mostly involving chronic migraineurs, because they evaluated diagnostic, prognostic, or predictive classes of biomarkers, i.e., did not report on monitoring or pharmacodynamic/response biomarkers collected after intervention (33 - 38), nine experiments carry out on cultured cells-derived from humans with the condition (androgenic alopecia, hypertrophic scar and keloid) (39 - 47), two studies on healthy individuals with experimentally induced chronic disease linked to inflammation (model of persistent myofascial temporomandibular disorder pain, trigeminal pain, neurogenic inflammation and cutaneous pain) (56, 57), ten studies providing bio functional measurements, physiological characteristics, and/or clinical outcome assessments rather than evaluating molecular or cellular biomarkers (48, 50, 51, 58 - 64), four studies on recalcitrant plaque psoriasis, multiple sclerosis with neurogenic detrusor overactivity, hypertrophic scars and keloids, that although reporting biomarkers change after BoNT administration, the targeted inflammation-associated chronic conditions were below head and neck (49, 52, 53, 55), and one study on unilateral intractable chronic occipital neuralgia reporting that serologic results were obtained before and after BoNT intervention but data was unavailable following the three attempts to contact the corresponding author (54). Noteworthy that given the inconsistencies between a published protocol (ClinicalTrials.gov, NCT02577185) and the corresponding published report (49), we have also unsuccessfully tried to contact another author for more information regarding the location of psoriasis vulgaris lesions prior to excluding from our review. We have tried to contact the corresponding author for the trial with the registration ClinicalTrials.gov NCT01071096 to have more information on the reported histological examinations (30); Although we were unable to confirm the location for the skin punch biopsies, this was not a criterium for exclusion. In addition, we excluded four documents that are ongoing clinical trials registered in ClinicalTrials.gov and awaiting publication of primary data (NCT03381261, NCT05720065, NCT00816517, NCT05456087) that may be included in a future update of this review.

| **INCLUDED REFERENCES** | **STUDY - LOE** | **CONDITION** | **BIOMARKER** | **INCLUSION/EXCLUSION** |
| --- | --- | --- | --- | --- |
| **INCLUDED with confidence** | | | | |
| **CITATION-Hand SEARCHING** | | | | |
| Gfrerer, 2022  Gfrerer, L., Xu, W., Austen, W., Ashina, S., Melo-Carrillo, A., Longhi, M. S., Adams, A. M., Houle, T., Brin, M. F., & Burstein, R. (2022). OnabotulinumtoxinA alters inflammatory gene expression and immune cells in chronic headache patients. *Brain : a journal of neurology*, *145*(7), 2436–2449. https://doi.org/10.1093/brain/awab461 | Clinical trial cohort LOE-III | Chronic bilateral occipital headache with migraine features | **Muscle, fascia, periosteum tissues from neck and occiput (occipitalis, spenius capitis, semispinalis capitis, trapezius)**  **Expression of inflammatory genes** - cytokine signaling, lymphocyte activation, innate immune response (T-cells – CD8, Th1(CD4+ T-cells); NK cells, B-cells, neutrophils, macrophages, dendritic cells), TNF family signaling, TLR signaling (TLR-regulating CD45 cells – capable of releasing cytokines IL1, IL6, TNF-α, IFNγ, CXCL2, CXCL10, CXCL8, IL13, IL12, IL23. (by transcriptome analyses) | √ |
| **DATABASES** | | | | |
| Zhang, 2020  Zhang, Y., Lian, Y., Zhang, H., Xie, N., & Chen, Y. (2020). CGRP Plasma Levels Decrease in Classical Trigeminal Neuralgia Patients Treated with Botulinum Toxin Type A: A Pilot Study. *Pain medicine (Malden, Mass.)*, *21*(8), 1611–1615. https://doi.org/10.1093/pm/pnaa028 | Clinical Pilot Study – LOE-III | Classical Trigeminal Neuralgia | **Plasma levels**  **Neuropeptide** – CGRP | √  “recent studies revealed that inflammation could have played a close and important role in the progression and etiology of TN.” Yao, Y., Chang, B., & Li, S. (2020). Relationship of Inflammation With Trigeminal Neuralgia. *The Journal of craniofacial surgery*, *31*(2), e110–e113. https://doi.org/10.1097/SCS.0000000000005879 |
| Cernuda-Morollón, 2015  Cernuda-Morollón, E., Ramón, C., Martínez-Camblor, P., Serrano-Pertierra, E., Larrosa, D., & Pascual, J. (2015). OnabotulinumtoxinA decreases interictal CGRP plasma levels in patients with chronic migraine. *Pain*, *156*(5), 820–824. https://doi.org/10.1097/j.pain.0000000000000119 | Prospective clinical study - LOE-III | Chronic Migraine | **Plasma levels**  **Neuropeptide** – CGRP | √ |
| Dini, 2019  Dini E, Mazzucchi S, De Luca C, Cafalli M, Chico L, Lo Gerfo A, Siciliano G, Bonuccelli U, Baldacci F, Gori S. **Plasma Levels of Oxidative Stress Markers**, before and after *BoNT/A* Treatment, in Chronic Migraine. *Toxins*. 2019; 11(10):608. <https://doi.org/10.3390/toxins11100608> | Clinical Trial  Prospective study – LOE-III | Chronic Migraine | **Plasma levels**  **Oxidative stress biomarkers** - AOPP, FRAP, SH | √  “The mechanisms underlying this chronicity are unresolved but are hypothesized to involve a degree of inflammation. relevant literature related inflammation and migraine, propose that the increase in migraine frequency leading to chronic migraine involves neurogenic neuroinflammation” |
| Cutrer, 2010  Cutrer F.M., Sandroni P., Wendelschafer-Crabb G. Botulinum toxin treatment of cephalalgia alopecia increases substance P and calcitonin gene-related peptide-containing cutaneous nerves in scalp. Cephalalgia. 2010;30:1000–1006. doi: 10.1111/j.1468-2982.2009.01987.x. | Single case experimental design – LOE-III | Cephalalgia Alopecia  (lymphocytic peribulbar inflammation similar to that seen in alopecia areata) | **Scalp**  **Neuropeptide** – CGRP, SP  **Nerve fibres** - PGP9.5^+^  **Mast cells** | √  “Alopecia areata is a chronic inflammatory non-scarring condition affecting the hair follicle that leads to hair loss” |
| Cady, 2014  Cady, R., Turner, I., Dexter, K., Beach, M. E., Cady, R., & Durham, P. (2014). An exploratory study of salivary calcitonin gene-related peptide levels relative to acute interventions and preventative treatment with onabotulinumtoxinA in chronic migraine. *Headache*, *54*(2), 269–277. https://doi.org/10.1111/head.12250 | Randomized, placebo-controlled, crossover pilot LOE-II | Chronic migraine | **Saliva**  CGRP | √ |
| Choi, 2019  Choi, M. G., Yeo, J. H., Kang, J. W., Chun, Y. S., Lee, J. K., & Kim, J. C. (2019). Effects of botulinum toxin type A on the treatment of dry eye disease and tear cytokines. *Graefe's archive for clinical and experimental ophthalmology = Albrecht von Graefes Archiv fur klinische und experimentelle Ophthalmologie*, *257*(2), 331–338. | Prospective randomized clinical trial LOE-II | Intractable dry eye disease (DED) | **Tear**  matrix metalloproteinase (MMP)-9 and serotonin | √  “ocular pain in DED has been proposed to be a kind of neuropathic pain” “Many patients diagnosed with DED report features of neuropathic pain, defined as pain resulting from nerve dysfunction” |
| Karakulova, 2017  Karakulova, Y. V., & Loginova, N. V. (2017). Éffektivnost' botulinoterapii v korrektsii stepeni bolevogo sindroma i kachestva zhizni patsientov s tservikal'noĭ distonieĭ [The efficacy of botulinotherapy in the correction of the pain syndrome and quality of life of patients with cervical dystonia]. *Zhurnal nevrologii i psikhiatrii imeni S.S. Korsakova*, *117*(12), 33–36. https://doi.org/10.17116/jnevro201711712133-36 | Open comparative follow-up clinical study LOE-III | Cervical dystonia with pain and depression | **Serum**  Blood platelet serotonin | √ cervical dystonia is not a chronic inflammatory condition. However, it was evaluated associated pain in the neck and degree of emotional stimulus (anxiety and depression) – neuroinflammation |
| ClinicalTrial.Gov | | | | |
| NCT01071096 | Completed with results | Chronic Migraine |  | Cady, 2014 |
| NCT02037425 Sponsor: Cady, Roger; Study competition: 2015-09; Last update: 2016-08-11  Exploratory Study of the Natural History, Clinical Outcomes, and Neuronal Endplate Changes in Subjects Reporting Short Duration vs. Long Duration of Benefit for OnabotulinumtoxinA in Treatment of Chronic Migraine | Completed with results LOE-III | Chronic Migraine | **Skin**  Change in neuronal regrowth in the skin biopsies | √ MISSING DATA contact the authors for biopsy location details. However, not a criterion for exclusion as it is a head and neck condition. Correspondence: email: rcady@headachecare.com |
| **UNCLEAR (EXCLUDED/INCLUDED) without confidence. Provisional Appendix 3** | | | | |
| **CITATION-Hand SEARCHING** | | | | |
| Hubbard, 2016  Hubbard, C.S.; Becerra, L.; Smith, J.H.; DeLange, J.M.; Smith, R.M.; Black, D.F.; Welker, K.M.; Burstein, R.; Cutter, F.M.; Borsook, D. Brain changes in responders vs. non-responders in chronic migraine: Markers of disease reversal. Front. Hum. Neurosci. 2016, 10, 497. | Retrospective clinical study LOE-IV | Chronic migraine | **Brain**  Cortical thickness | √ ? Unclear? (BIO) FUNCTIONAL MEASUREMENTS? Cortex measurements only taken after treatment?  morphometric and functional brain changes between responders and not-responders. However, it is unclear the premorbid brain state (i.e., no baseline cortex measurements to clarify whether drug or placebo) and its contributions to the observed morphological and functional brain changes and whether these changes represent reliable markers of migraine chronification and reversal. |
| Todberg, 2018 **NCT02577185**  Todberg T., Zachariae C., Bregnhoj A., Hedelund L., Bonefeld K.K., Nielsen K., Iversen L., Skov L. The effect of botulinum neurotoxin A in patients with plaque psoriasis—An exploratory trial. J. Eur. Acad. Dermatol. Venereol. 2018;32:e81–e82. doi: 10.1111/jdv.14536. | Randomised double-blind LOE-II | Plaque Psoriasis | **Lesional & perilesional skin**  **Epidermal density** - ENFs  **Neuropeptides -** SP and CGRP | √ CONDITION/LESIONS NOT in HEAD & NECK (although it reported biomarkers evaluating BoNTA effect) Study protocol register (ClinicalTrials.gov, ID: NCT02577185) refers on inclusion criteria: “A diagnosis of psoriasis vulgaris with lesions located on arms and/or legs and/or trunk.”, only. However, there were discrepancies between register and published article. Thus, it should be confirmed that no head and neck lesions prior to exclusion. Correspondence: T. Todberg. E-mail: [tanja.todberg@regionh.dk](mailto:tanja.todberg@regionh.dk) |
| Kim, 2019  Kim MJ, Kim JH, Cheon HI, Hur MS, Han SH, Lee YW, et al. Assessment of Skin Physiology Change and Safety After Intradermal Injections With Botulinum Toxin: A Randomized, Double-Blind, Placebo-Controlled, Split-Face Pilot Study in Rosacea Patients With Facial Erythema. Dermatol Surg. 2019;45(9):1155–62. | Randomized, Double-Blind, Placebo-Controlled LOE-II | Rosacea | Clinician Erythema Assessment (CEA) score, Global Aesthetic Improvement Scale (GAIS) score, **skin hydration, transepidermal water loss (TEWL), melanin content, erythema index, elasticity, and sebum secretions (corneometer, mexameter, reviscometer, sebumeter)** | √ ? Unclear? (BIO) FUNCTIONAL MEASUREMENTS? biomarker for healthy skin  The change in quantified biophysical measurements provides a reliable signal for the expected therapeutic response, or these easily measurable biophysical do not reflect true pharmacodynamic responses?  These are instead clinical outcome assessments (COAs) that are directly important to the patients rather than biomarkers that serve to link a measurement to a prediction of COAs? |
| Bumb, 2013  Bumb, A.; Seifert, B.; Wetzel, S.; Agosti, R. Patients profiling for Botox (onabotulinum toxin A) treatment for migraine: A look at white matter lesions in the MRI as a potential marker. Springer Plus 2013, 2, 377. | Retrospective observational study | Migraine | **Axial T2 and coronar FLAIR (fluid attenuated inversion recovery) sequences -** white matter lesions (WML) | √ ? Unclear? (BIO) FUNCTIONAL MEASUREMENTS? marker only collected before BoNTA treatment - tested as predictor for favourable response. |
| Khatery, 2022  Khatery, B. H. M., Hussein, H. A., Abd-El-Raheem, T. A., El Hanbuli, H. M., & Yassen, N. N. (2022). Assessment of intralesional injection of botulinum toxin type A in hypertrophic scars and keloids: Clinical and pathological study. *Dermatologic therapy*, *35*(10), e15748. https://doi.org/10.1111/dth.15748 | Clinical study | Hypertrophic scars and keloids | Histologic grading scores - quality of collagen and elastic tissues.  Image analysis to detect their quantitative morphometric changes.  Clinical assessment: Vancouver Scar Scale (VSS), Observer Scar Assessment Scale (OSAS), and the Patient Scar Assessment Scale (PSAS). | √ CONDITION/LESIONS NOT in HEAD & NECK (although it reported biomarkers evaluating BoNTA effect) “Exclusion criteria: Pregnancy, lactation, lesions in the face and neck area, lesions size less than 2 cm, allergy to BTX-A and previous treatment in the last 6 months” |
| **DATABASES** | | | | |
| Aschenbeck, 2018  Aschenbeck K.A., Hordinsky M.K., Kennedy W.R., Wendelschafer-Crabb G., Ericson M.E., Kavand S., Bertin A., Dykstra D.D., Panoutsopoulou I.G. Neuromodulatory treatment of recalcitrant plaque psoriasis with onabotulinumtoxinA. J. Am. Acad. Dermatol. 2018;79:1156–1159. doi: 10.1016/j.jaad.2018.07.058. | Single-centre Clinical Pilot Study -LOE-IV | Recalcitrant plaque psoriasis | **Lesional & perilesional skin**  **Epidermal density -** ENFs  **Neuropeptides -** SP and CGRP | √ CONDITION/LESIONS NOT in HEAD & NECK (although it reported biomarkers evaluating BoNTA effect) (elbow, back, knee, leg, foot)  “Psoriasis is a chronic, relapsing immune-mediated inflammatory disease” |
| Kim, 2021  Kim, H., Jang, B., & Kim, S. T. (2021). Botulinum Toxin Type-A (Botulax^®^) Treatment in Patients with Intractable Chronic Occipital Neuralgia: A Pilot Study. *Toxins*, *13*(5), 332. https://doi.org/10.3390/toxins13050332 | Pilot study – clinical trial  LOE -IV | Unilateral intractable chronic occipital neuralgia | Blood – serologic, hematologic exam  pain visual analog scale (VAS), quality of life | √ (?) MISSING PRIMARY OUTCOME  Needs contacting the authors as no values for serologic evaluation given – primary outcome Correspondence: [ca.shuy@0506578k](mailto:dev@null) |
| Philippova, 2021  Philippova, E. S., Bazhenov, I. V., Ziryanov, A. V., & Bazarny, V. V. (2021). Impact of intradetrusor botulinum toxin A injections on serum and urinary concentrations of nerve growth factor and brain-derived neurotrophic factor in patients with multiple sclerosis and neurogenic detrusor overactivity. *Neurourology and urodynamics*, *40*(1), 95–101. https://doi.org/10.1002/nau.24534 | Prospective cohort LOE-III | Multiple Sclerosis and neurogenic detrusor overactivity | **Serum & Urine levels**  **Neurotrophic factors** – BDNF, NGF | √ CONDITION/LESIONS NOT in HEAD & NECK (although it reported biomarkers evaluating BoNTA effect) “Multiple sclerosis (MS) is a chronic inflammatory disease of the central nervous system (CNS)” Yonnet, G. J., Fjeldstad, A. S., Carlson, N. G., & Rose, J. W. (2013). Advances in the management of neurogenic detrusor overactivity in multiple sclerosis. *International journal of MS care*, *15*(2), 66–72. <https://doi.org/10.7224/1537-2073.2012-031> |
| Gazerani, 2008  Gazerani, P., Pedersen, N. S., Staahl, C., Drewes, A. M., & Arendt-Nielsen, L. (2009). Subcutaneous Botulinum toxin type A reduces capsaicin-induced trigeminal pain and vasomotor reactions in human skin. *Pain*, *141*(1-2), 60–69. https://doi.org/10.1016/j.pain.2008.10.005 | Randomised clinical trial | capsaicin-induced trigeminal pain, neurogenic inflammation and experimentally induced cutaneous pain modalities. | pain intensity, pain area, area of secondary hyperalgesia, area of visible flare and vasomotor reactions, cutaneous heat, electrical and pressure pain thresholds. | HEALTHY INDIVIDUALS - EXPERIMENTALLY INDUCED-DISEASE? Unclear? (BIO) FUNCTIONAL MEASUREMENTS? |
| da Silva, 2014  da Silva LB, Kulas D, Karshenas A, et al. Time course analysis of the effects of botulinum neurotoxin type A on pain and vasomotor responses evoked by glutamate injection into human temporalis muscles. *Toxins (Basel)*. 2014;6(2):592-607. Published 2014 Feb 10. doi:10.3390/toxins6020592 | Randomised clinical trial | pain and vasomotor responses evoked by glutamate injection into human temporalis muscles – model of persistent myofascial TMD pain | Pain intensity, pain area, and neurogenic inflammation (skin temperature and skin blood perfusion) | HEALTHY INDIVIDUALS - EXPERIMENTALLY INDUCED-DISEASE? Unclear? (BIO) FUNCTIONAL MEASUREMENTS? thermal imaging of the muscle “Experimental designs based on glutamate injection into muscle can provide an appropriate model for elucidating persistent myofascial pain conditions.” Castrillon, E. E., Cairns, B. E., Ernberg, M., Wang, K., Sessle, B., Arendt-Nielsen, L., & Svensson, P. (2008). Glutamate-evoked jaw muscle pain as a model of persistent myofascial TMD pain?. *Archives of oral biology*, *53*(7), 666–676. https://doi.org/10.1016/j.archoralbio.2008.01.008 |
| Reyes, 2023  Reyes, N., Huang, J. J., Choudhury, A., Pondelis, N., Locatelli, E. V., Felix, E. R., Pattany, P. M., Galor, A., & Moulton, E. A. (2023). Botulinum toxin A decreases neural activity in pain-related brain regions in individuals with chronic ocular pain and photophobia. *Frontiers in neuroscience*, *17*, 1202341. https://doi.org/10.3389/fnins.2023.1202341 | Cohort LOE-III | chronic ocular pain and photophobia | whole brain blood oxygen level dependent (BOLD) responses to light stimuli, functional magnetic resonance imaging (fMRI).  collected tear parameters - tear breakup time, fluorescein corneal staining (indicating regular/irregular epithelium), and tear production Schirmer strips test (measures tear production) | √ ? Unclear? (BIO) FUNCTIONAL MEASUREMENTS? “immune disorders have been most closely linked to chronic ocular surface pain” Mehra, D., Cohen, N. K., & Galor, A. (2020). doi.org/10.1007/s40123-020-00263-9. “The variability of symptom presentation and of ocular surface exam findings suggest that chronic ocular pain can be driven by both nociceptive and neuropathic mechanisms. Nociceptive sources of pain, including inflammation and epithelial disruption” |
| Borodic, 2014  Borodic, G. E., Caruso, P., Acquadro, M., & Chick, S. (2014). Parry-Romberg syndrome vasculopathy and its treatment with botulinum toxin. *Ophthalmic plastic and reconstructive surgery*, *30*(1), e22–e25. https://doi.org/10.1097/IOP.0b013e31828de9c0 | Case report LOE-V | Parry-Romberg syndrome vasculopathy with chronic pain and atrophy – right brow, forehead, and scalp – and progressive hair loss | Muscle and skin - focal inflammation (baseline biopsy only)  Facial and orbit atrophy (MRI)  Brain – blood flow (MRI) | √ ? Unclear? (BIO) FUNCTIONAL MEASUREMENTS? (MRI not evaluating BoNTA effects?)  “Various mechanisms have been proposed to explain the development of the disorder, including inflammation in the nerves supplying skin and fat causing an autoimmune reaction, disturbance of fat metabolism, trauma, infection, vascular malformations, trigeminal neurovasculitis, autoimmune, hyperactivity of the brain stem, cervical sympathetic dysfunction, and genetics.” [Gary L. Legault, MD](https://eyewiki.org/User:Gary.L.Legault), [Krista Stewart, MD](https://eyewiki.org/User:Krista.Stewart), [Michael T Yen, MD](https://eyewiki.org/User:Michael.Yen), [Cat Nguyen Burkat, MD FACS](https://eyewiki.org/User:Cat.N.Burkat) |
| Sebastianelli, 2023  Sebastianelli, G., Casillo, F., Di Renzo, A., Abagnale, C., Cioffi, E., Parisi, V., Di Lorenzo, C., Serrao, M., Pierelli, F., Schoenen, J., & Coppola, G. (2023). Effects of Botulinum Toxin Type A on the Nociceptive and Lemniscal Somatosensory Systems in Chronic Migraine: An Electrophysiological Study. *Toxins*, *15*(1), 76. https://doi.org/10.3390/toxins15010076 | Clinical trial | Chronic migraine | cephalic and extracephalic nociceptive and lemniscal sensory systems - nociceptive blink reflex (nBR), trigemino-cervical reflex (nTCR), pain-related cortical evoked potential (PREP), upper limb somatosensory evoked potential (SSEP) | √ ? Unclear? (BIO) FUNCTIONAL MEASUREMENTS? |
| Valente, 2021  Valente, M., Lettieri, C., Russo, V., Janes, F., & Gigli, G. L. (2021). Clinical and Neurophysiological Effects of Botulinum Neurotoxin Type A in Chronic Migraine. *Toxins*, *13*(6), 392. https://doi.org/10.3390/toxins13060392 | Clinical trial | Chronic migraine | transcranial magnetic stimulation (TMS) to evaluate changes in cortical excitability and plasticity | √ ? Unclear? (BIO) FUNCTIONAL MEASUREMENTS? |
| Ozarslan, 2022  Ozarslan, M., Matur, Z., Tuzun, E., & Oge, A. E. (2022). Cutaneous allodynia and thermal thresholds in chronic migraine: The effect of onabotulinumtoxinA. *Clinical neurology and neurosurgery*, *220*, 107357. https://doi.org/10.1016/j.clineuro.2022.107357 | Clinical trial | Chronic migraine (cutaneous allodynia) | heat and cold detection thresholds on the forehead and hand measured bilaterally with [quantitative sensory testing](https://www.sciencedirect.com/topics/medicine-and-dentistry/quantitative-sensory-testing) (computer-assisted sensory examination) | √ ? Unclear? (BIO) FUNCTIONAL MEASUREMENTS? QST assess the functions of large and small myelinated and unmyelinated sensory fibres and the relevant pathways |
| de Tommaso, 2016  de Tommaso, M., Delussi, M., Ricci, K., Montemurno, A., Carbone, I., & Vecchio, E. (2016). Effects of OnabotulintoxinA on Habituation of Laser Evoked Responses in Chronic Migraine. *Toxins*, *8*(6), 163. https://doi.org/10.3390/toxins8060163 | Clinical trial double blind placebo controlled crossover design | Chronic migraine | Laser evoked potentials (LEPs)/ neurophysiologic tool changes at the main sites of injection, as frontal and trapezius sites, with a control site as the hand dorsum.  Correlate main LEPs findings with clinical outcome (MIDAS score) | √ ? Unclear? (BIO) FUNCTIONAL MEASUREMENTS? “evaluate pain processing modifications in chronic migraine patients under single BontA administration in pericranial muscles, by means of CO^2^ Laser Evoked Potentials (LEPs) obtained by the stimulation of the skin over the right frontal and trapezius injection sites and hand dorsum” |
| Lee, 2016  Lee, M. J., Lee, C., Choi, H., & Chung, C. S. (2016). Factors associated with favorable outcome in botulinum toxin A treatment for chronic migraine: A clinic-based prospective study. *Journal of the neurological sciences*, *363*, 51–54. https://doi.org/10.1016/j.jns.2016.01.054 | Clinic-based prospective study | Chronic migraine | Korean version of the Headache Impact Test-6 (HIT-6), interstitial Transcranial Doppler testing (TCD). Mean flow velocities (MFVs) of each vessel. Ratios of intracranial arteries to extracranial ones (MCA/ICA index) | √ ? Unclear? (BIO) FUNCTIONAL MEASUREMENTS? TCD only evaluated pretreatment. To calculate predicting value |
| **NO RESULTS PUBLISHED** | | | | |
| **CITATION-Hand SEARCHING** | | | | |
| Ernberg, 2023*  **https://www.clinicaltrials.gov/ct2/show/NCT05720065*** | Randomized, Controlled, Double-blind Study (Phase 2) | Peripheral TMD Pain | **Gene expression measured with bulk RNA-seq**  **Epigenetic signature measured with ATAC**  **Expression of sensory neuron markers measured with IHC** | √ (?) no results available yet |
| **ClinicalTrial.Gov** | | | | |
| NCT03381261 William G. Austen  Novel Concepts for OnabotulinumtoxinA (Botox) Mechanisms of Action: Role in Altering the Molecular Environment in Which Pain Fibers Exist | Active, not recruiting | Migraine | molecular markers in discarded tissue of migraine surgery will be analyzed. Gene Expression Code Set profiling 594 genes; 579 immunology-related human genes + 15 internal reference controls. | X no results posted |
| NCT05720065* | Not yet recruiting | Temporomandibular Disorders | Gene expression measured with bulk RNA-seq  Epigenetic signature measured with ATAC  Expression of sensory neuron markers measured with IHC | X no results yet |
| NCT00816517 | Completed | Psoriasis Vulgaris | psoriasis scoring scale, skin biopsy (3mm) | X ? no results, head and neck? |
| NCT05456087 | Recruiting | Androgenic Alopecia | hair density (count/cm2), hair shaft diameter, follicular units, among other measurements captured by the software - trichoscopy imaging using the Canfield HairMetrix® system | Inflammation? biomarkers ?  X no results yet |
| **INCLUDED – Appendix 1 (Diagnostic, Prognostic, or Predictive biomarkers – not evaluated after intervention)** | | | | |
| **CITATION-Hand SEARCHING** | | | | |
| Cernuda-Morollón, 2014  Cernuda-Morollón, E., Martínez-Camblor, P., Ramón, C., Larrosa, D., Serrano-Pertierra, E., & Pascual, J. (2014). CGRP and VIP levels as predictors of efficacy of Onabotulinumtoxin type A in chronic migraine. *Headache*, *54*(6), 987–995. https://doi.org/10.1111/head.12372 | Prospective clinical study-LOE-III | Chronic Migraine | **Plasma levels**  **Neuropeptide** – CGRP, VIP | √ blood samples were only collected before BoNTA treatment. Biomarker tested as predictor for favourable response |
| **DATABASES** | | | | |
| Leira, 2021  Leira, Y., Domínguez, C., Ameijeira, P., López-Arias, E., Ávila-Gómez, P., Pérez-Mato, M., Sobrino, T., Campos, F., Blanco, J., & Leira, R. (2021). Mild systemic inflammation enhances response to OnabotulinumtoxinA in chronic migraineurs. *Scientific reports*, *11*(1), 1092. https://doi.org/10.1038/s41598-020-80283-4 | Cross-sectional clinical study from previous observational studies – LOE-IV | Chronic Migraine and Periodontitis | **Serum levels Neuropeptide** - CGRP  **Cytokines** - IL‑6, IL‑10  **Systemic inflammatory mediator -** CRP | √ blood samples were only collected before BoNTA treatment. Biomarker tested as predictor for favourable response  “Periodontitis is a common inflammatory disease of infectious origins that often evolves into a chronic condition.” Martínez-García, M., & Hernández-Lemus, E. (2021). Periodontal Inflammation and Systemic Diseases: An Overview. *Frontiers in physiology*, *12*, 709438. https://doi.org/10.3389/fphys.2021.709438 |
| Domínguez, 2018  Domínguez, C., Vieites-Prado, A., Pérez-Mato, M., Sobrino, T., Rodríguez-Osorio, X., López, A., Campos, F., Martínez, F., Castillo, J., & Leira, R. (2018). CGRP and PTX3 as Predictors of Efficacy of Onabotulinumtoxin Type A in Chronic Migraine: An Observational Study. *Headache*, *58*(1), 78–87. https://doi.org/10.1111/head.13211 | Observational clinical prospective study LOE-III | Chronic Migraine | **Serum levels**  **Cytokines** - IL‑6, IL‑10**,** TNF-α  **Systemic inflammatory mediators –** CRP  **Endothelial dysfunction –** PTX3, sTWEAK  **Blood-brain barrier disruption –** cFN  **Brain damage –** S100b, NSE  **Trigeminal-vascular activation Inflammation Neuropeptide** – CGRP | √ biomarkers only collected before BoNTA treatment. Biomarker tested as predictor for favourable response |
| Moreno-Mayordomo, 2019  Moreno-Mayordomo, R., Ruiz, M., Pascual, J., Gallego de la Sacristana, M., Vidriales, I., Sobrado, M., Cernuda-Morollon, E., Gago-Veiga, A. B., Garcia-Azorin, D., Telleria, J. J., & Guerrero, A. L. (2019). CALCA and TRPV1 genes polymorphisms are related to a good outcome in female chronic migraine patients treated with OnabotulinumtoxinA. *The journal of headache and pain*, *20*(1), 39. https://doi.org/10.1186/s10194-019-0989-9 | Prospective, observational, multicenter clinical study | Chronic migraine | **Peripheral blood (genomic DNA)**  **Genes and 25 single nucleotide polymorphisms (SNPs):**  **Genes related to glutamate homeostasis –** MEF2D, LRP1, MTDH, EAAT2, GRIK3  **Gene which encodes for CGRP – CALCA**  **Genes of GABA system –** GABRE, GABRQ, GABRA3  **Genes which encode voltage dependent channels –** SCN9A, KCNS1, P2RX7  **Gene which encodes D2dopamine receptor –** DRD2(ANKK1)  **Gene of 5-HT2C serotonin receptor –** HTR2C  **Genes of the TRP family** – TRPV1, TRPV3, TRPM8  **Other genes –** WFS1, TGFBR2, MTHFR | √ biomarkers only collected before BoNTA treatment. Biomarker tested as predictor for favourable response |
| Domínguez Vivero, 2020  Domínguez Vivero, C., Leira, Y., Saavedra Piñeiro, M., Rodríguez-Osorio, X., Ramos-Cabrer, P., Villalba Martín, C., Sobrino, T., Campos, F., Castillo, J., & Leira, R. (2020). Iron Deposits in Periaqueductal Gray Matter Are Associated with Poor Response to OnabotulinumtoxinA in Chronic Migraine. *Toxins*, *12*(8), 479. https://doi.org/10.3390/toxins12080479 | Prospective cohort clinical study LOE III | Chronic migraine | **Plasma (**during interictal periods) **-** calcitonin gene-related peptide (CGRP) and pentraxin-3 (PTX3)  **Neuroimaging changes** - iron deposits in the red nucleus (RN), substantia nigra (SN), globus pallidus (GP), and periaqueductal gray matter (PAG), and white matter lesions (WML) | √ biomarkers only collected before BoNTA treatment. Biomarker tested as predictor for favourable response |
| Cutrer, 2006  Cutrer FM, Pittelkow MR (2006) Cephalalgic alopecia areata: a syndrome of neuralgiform head pain and hair loss responsive to botulinum A toxin injection. Cephalalgia 26(6):747–751 | Case report LOE V | Cephalalgia - alopecia areata | MRI, blood samples, scalp biopsy | √ ? no correlation between responders/ non-responders vs diagnostic biomarkers. However provided record for an included study based on the same participant  “Biopsy and Blood testing for inflammatory markers including erythrocyte sedimentation rate (ESR), rheumatoid factor (RF), antinuclear antibody (ANA) and antibodies to extractable nuclear antigens (ENA) was only mentioned for diagnosis purposes, not to evaluate BoNTA. This seems to have been done through clinical parameters” |
| **INCLUDED – Appendix 2 (Ex vivo and in vitro studies - cultured cells-derived from humans with the condition)** | | | | |
| **CITATION Hand SEARCHING** | | | | |
| Xiaoxue, 2014  Xiaoxue, W., Xi, C., & Zhibo, X. (2014). Effects of botulinum toxin type A on expression of genes in keloid fibroblasts. *Aesthetic surgery journal*, *34*(1), 154–159. https://doi.org/10.1177/1090820X13482938 | Clinical trial* | Keloid fibroblast | **Keloid-derived fibroblast:**  112 genes (S100A4, TGF-β1, VEGF, MMP-1, and PDGFA) relevant to invasive growth (microarray analysis) to study messenger RNA expression profiles (qRT-PCR) | √ experiments on cultured cells-derived from humans with the condition. head and neck? Did not specify hypertrophic scar location “Keloid and hypertrophic scars are the result of chronic inflammation” Ogawa,2017 |
| Gauglitz, 2012  Gauglitz GG, Bureik D, Dombrowski Y, Pavicic T, Ruzicka T, Schauber J: Botulinum toxin A for the treatment of keloids. Skin Pharmacol Physiol 2012;25:313–318 | Clinical trial* | Keloids fibroblasts | **Keloid-derived fibroblasts:**  **ECM markers -** fibronectin-1, laminin-β2, α-SMA. **Markers for collagen synthesis or TGF-β1, TGF-β2, and TGF-β3 proteins** | √ experiments on cultured cells-derived from humans with the condition. head and neck? Did not specify the keloid location  “Keloid and hypertrophic scars are the result of chronic inflammation” Ogawa,2017 |
| Xiao, 2010  Xiao Z, Zhang F, Lin W, Zhang M, Liu Y: Effect of botulinum toxin type A on transforming growth factor beta1 in fibroblasts derived from hypertrophic scar: a preliminary report. Aesthetic Plast Surg 2010;34:424–427. | Clinical trial* | Hypertrophic scars fibroblasts | **fibroblasts derived from hypertrophic scar:**  transforming growth factor-β1(TGF-β1) | √ experiments on cultured cells-derived from humans with the condition. head and neck? Did not specify hypertrophic scar location “Keloid and hypertrophic scars are the result of chronic inflammation” Ogawa,2017 |
| Hao, 2018  Hao R, Li Z, Chen X, Ye W. Efficacy and possible mechanisms of botulinum toxin type A on hypertrophic scarring. J Cosmet Dermatol. 2018;17(3):340‐346. | Clinical trial | Keloid fibroblast | **Main factors influencing collagen degradation of keloid tissue**: transforming growth factor (TGF)-b1, matrix metalloproteinase (MMP)-1, MMP-2, and MMP-9  **Changes in cellular morphology, viability, proliferation, cell cycle, and apoptosis** | √ experiments on cultured cells-derived from humans with the condition. Head and neck location? “Keloids and hypertrophic scars are pathological cutaneous scars. They arise from excessive wound healing, which induces chronic dermal inflammation and results in overwhelming fibroblast production of extracellular matrix.” |
| Park, 2019  Park GS, An MK, Yoon JH, et al. Botulinum toxin type A suppresses pro‐fibrotic effects via the JNK signaling pathway in hypertrophic scar fibroblasts. Arch Dermatol Res. 2019;311(10):807‐814. | Clinical trial * | Hypertrophic scar fibroblasts | **Fibroblast - proliferation, migration**  **Protein expression of pro-fibrotic factors -** transforming growth factor β1, IL-6, connective tissue growth factor | √ experiments on cultured cells-derived from humans with the condition. Head and neck location? “human scar fibroblasts were cultured” |
| Xiao, 2011  Xiao Z, Zhang M, Liu Y, Ren L (2011) Botulinum toxin type A inhibits connective tissue growth factor expression in fibroblasts derived from hypertrophic scar. Aesthetic Plast Surg 35:802–807. https://doi.org/10.1007/s00266-011-9690-3 | Clinical trial* | Hypertrophic scar | **Hypertrophic scar-derived fibroblasts**  Proliferation  Proteins of connective tissue growth factor | √ experiments on cultured cells-derived from humans with the condition. Head and neck location? |
| Jeong, 2015  Jeong, H. S., Lee, B. H., Sung, H. M., Park, S. Y., Ahn, D. K., Jung, M. S., & Suh, I. S. (2015). Effect of Botulinum Toxin Type A on Differentiation of Fibroblasts Derived from Scar Tissue. *Plastic and reconstructive surgery*, *136*(2), 171e–178e. https://doi.org/10.1097/PRS.0000000000001438 | Clinical study * in vitro | Hypertrophic scar | Fibroblasts-derived hypertrophic scars  Fibroblast differentiation to myofibroblast.  α-smooth muscle actin mRNA and protein levels | √ experiments on cultured cells-derived from humans with the condition. Head and neck location? |
| **DATABASES** | | | | |
| Shon, 2020  Shon U., Kim M.H., Lee D.Y., Kim S.H., Park B.C. The effect of intradermal botulinum toxin on androgenetic alopecia and its possible mechanism. J. Am. Acad. Dermatol. 2020;83:1838–1839. doi: 10.1016/j.jaad.2020.04.082. | Clinical study and * in vitro study | Androgenic alopecia | **Balding Scalp and *hair bulb – cultured Dermal papilla cells (DPCs)**  Transforming growth factor β1 (TGF-β1) | √ experiments on cultured cells-derived from humans with the condition. “Androgenic alopecia, also known as pattern hair loss, is a chronic progressive condition” Robert S. English,A hypothetical pathogenesis model for androgenic alopecia: clarifying the dihydrotestosterone paradox and rate-limiting recovery factors, Medical Hypotheses, Volume 111, 2018, Pages 73-81, ISSN 0306-9877, <https://doi.org/10.1016/j.mehy.2017.12.027>.  “Evidence and implications of inflammation as a characteristic feature of MPHL and FPHL are highlighted through evaluation of clinical and quantitative data.” Peyravian, N., Deo, S., Daunert, S., & Jimenez, J. J. (2020). The Inflammatory Aspect of Male and Female Pattern Hair Loss. *Journal of inflammation research*, *13*, 879–881. https://doi.org/10.2147/JIR.S275785 |
| Zhang, 2022*  Zhang, S., Li, K., Yu, Z., Chai, J., Zhang, Z., Zhang, Y., & Min, P. (2022). Dramatic Effect of Botulinum Toxin Type A on Hypertrophic Scar: A Promising Therapeutic Drug and Its Mechanism Through the SP-NK1R Pathway in Cutaneous Neurogenic Inflammation. *Frontiers in medicine*, *9*, 820817. https://doi.org/10.3389/fmed.2022.820817 | Clinical and Preclinical animal study | Hypertrophic scar | **Scar tissue**  scar thickness and deposition of collagen, cell mobilization | √ experiments on cultured cells-derived from humans with the condition. Head and neck location? pre-treatment with BoNTA – scar formation (not chronic inflammation) |
