# Supplementary material for: Botulinum toxin effects on biochemical biomarkers related to inflammation-associated head and neck chronic conditions: a systematic review of clinical research
Source: J Neural Transm (Vienna). 2025 Mar 4;132(12):1851–74. doi: 10.1007/s00702-024-02869-w (PMC12669376; doi:10.1007/s00702-024-02869-w)
Supplement: Supplementary file 5 — Supplementary file5 (DOCX 29 KB) [file 702_2024_2869_MOESM5_ESM.docx]

**Supplementary Information 4. Table 2:** Biomarkers in Clinical Research of Botulinum Toxin effects on Chronic Inflammatory State. (Diagnostic, Prognostic, or Predictive biomarkers; Not Monitoring or Pharmacodynamic/response biomarkers collected after intervention)

| **CLINICAL STUDIES** | | | | |
| --- | --- | --- | --- | --- |
| **Author, Year** | **Study Design Population**  **LOE** | **Condition**  **CIS** | **Biological sampling Biomarker** | **BoNTA**  **Key effect**  **Key mechanism** |
| **INFLAMMATION-NEUROGENIC INFLAMMATION-NEUROINFLAMMATION** | | | | |
| Leira, 2021 | (n=61) (60F,1M)  Follow-up:  24 weeks  BoNTA - 155–195 U, 31–39 precranial injections, twice over two consecutive periods of 12 weeks (PREEMPT protocol)  G1. Responders (n=7)  G2. Poor-responders (n=54)  Cross-sectional design from previous observational studies. LOE III | Chronic migraine and Periodontitis | **Serum levels Neuropeptide** - CGRP **Cytokines** - IL‑6, IL‑10  **Systemic inflammatory mediators –** CRP  measure by the enzyme-linked immunosorbent assay and immunodiagnostic IMMULITE 2000 Systems | •G1: ≥ 50% reduction in frequency of headache  •Periodontitis  70.4% G1, 28.6% G2 (P = 0.042)  •G1 ↑ levels of inflammation than G2:  IL‑6: 15.3 ± 8.7 vs. 9.2 ± 4.7 ng/mL, P = 0.016;  CGRP: 18.8 ± 7.6 vs. 13.0 ± 3.1 pg/mL, P = 0.002;  CRP: 3.9 ± 6.6 vs. 0.9 ± 0.8 mg/L, P = 0.003  •Linear positive correlation between the amount of periodontal tissue inflamed and markers of inflammation (IL‑6: r = 0.270, P = 0.035; CGRP: r = 0.325, P = 0.011; CRP: r = 0.370, P = 0.003)  •↑ systemic inflammatory markers related to periodontitis, BoNTA seems to ↓ migraine attacks.  Other measurements: Full-mouth clinical periodontal measurements |
|  | LIMITATIONS: blood inflammatory markers were determined only prior to BoNTA. some of the patients could have other undiagnosed conditions linked to increased systemic inflammation. Small sample size especially for non-responders, and short follow-up. | | | |
| Domínguez Vivero, 2020 | (n=62)  BoNTA following PREEMPT protocol  Follow-up: 12 weeks after treatment  G1. (n=47) responders (≥50% reduction in headache days)  G2. (n=15) non-responders (<50%)  Prospective observational cohort study LOE-III | Chronic migraine  (International Classification of Headache Disorders, 3rd edition criteria) | **Plasma (**during interictal periods) **-** calcitonin gene-related peptide (CGRP) and pentraxin-3 (PTX3)  **Neuroimaging changes** - iron deposits in the red nucleus (RN), substantia nigra (SN), globus pallidus (GP), and periaqueductal gray matter (PAG), and white matter lesions (WML) | •Responders to treatment were younger (mean age difference=12.2; 95% confidence interval (CI): 5.4–18.9, p=0.001), showed ↑ serum levels of CGRP (≥50 ng/mL) and PTX3 (≥1000 pg/mL) and smaller iron deposits in the GP and PAG (mean difference=805.0; 95% CI: 37.9–1572.1 μL, p=0.040 and mean difference=69.8; 95% CI: 31.0–108.6 μL, p=0.008; respectively).  •38 of 47 (80.9%) chronic migraineurs in G1 presented significantly ↑ serum levels of CGRP (≥50 ng/mL) compared to 4 out of 15 (26.7%) in G2. •Similarly, 87.2% (41/47) in G1 had ↑ serum levels of PTX3 (≥1000 pg/mL) in comparison to 20.0% (3/15) in G.  •Differences in PAG iron deposits remained significant after adjusting for age (mean difference=65.7; 95% CI: 22.8–108.6 μL, p=0.003) and remained significantly associated with poor response to BoNTA after adjustment for clinical and biochemical variables (odds ratio (OR)=0.963; 95% CI: 0.927–0.997, p =0.041).  •It was concluded that larger PAG iron deposits are associated with poor response to BoNTA in chronic migraine. A 10% ↑ in iron ground volumes in the PAG was associated with an odds ratio for poor response to treatment of 0.973 (95% CI: 0.955–0.991, p = 0.040) independently of age and GP. •After adjustment for biochemical variables, larger iron deposits in the PAG remained significantly associated with poor response to BoNTA (odds ratio (OR) = 0.963; 95% CI: 0.927–0.997, p = 0.041).  •Association was found between larger iron deposits in the PAG and a poor response to BoNTA in CM patients. These findings could be the neuroimaging correlate of clinical predictors of response such as duration of disease, frequency of attacks, or allodynia. |
|  | LIMITATIONS: short follow-up (12-week), some clinical variables that have been reported to predict efficacy of BoNTA were not used, such as laterality of pain or imploding characteristics of pain. The study used headache diaries to register headache days after treatment, but baseline frequency is based on patient recall, which could bias the results. Preventive treatments were used concurrently with BoNTA were not considered in the analysis, and they could have influenced the results. | | | |
| Domínguez, 2018 | (n=86)  Follow-up: 12, 24 weeks from 1^st^ treatment.  BoNTA  PREEMPT protocol  G1. CM (n=62)  G1a responders (n=47/75.8%)  G1b non-responders (n=15/24.2%)  G2. HG (n=24)  Observational prospective study LOE-III | Chronic migraine (IHS 2013 criteria) | **Serum levels**  **Cytokines** - IL‑6, IL‑10**,** TNF-α  **Systemic inflammatory mediators –** CRP  **Endothelial dysfunction –** PTX3, sTWEAK  **Blood-brain barrier disruption –** cFN  **Brain damage –** S100b, NSE  **Trigeminal-vascular activation Inflammation Neuropeptide** – CGRP  determined using commercial ELISA kits and immunodiagnostic IMMULITE 1000 System. | •G1a: >50% improvement in headache frequency.  Baseline blood samples only:  •G1a vs G1b:  PTX3 and CGRP levels significantly ↑ in G1a than G1b. PTX3 significantly ↑ in excellent responders vs G1b. CGRP significantly ↑ excellent responders vs moderate responders.  PTX3 levels – G1a (1455.4±487.5 pg/mL), G1b (720.3±334.1 pg/mL, P < 0.0001),  CGRP levels – G1a (133.1±86.6 ng/mL), G1b (58.2±91.7 ng/mL, P = 0.004)  No significant differences in the value of other molecules between G1a and G1b.  •Between excellent responders (n=11) and G1b: significant differences in TNF-α (8.0±1.4 vs 5.9±1.4 pg/mL; P= 0.47), IL-6 (11.7±1.3 vs 7.4±2.3 ng/mL; P=0.001) and cFn (14.9±5.3 vs 11.4±6.5 µg/mL; P = 0.025).  •Between G1b and G2: significant differences in IL-6 (7.4±2.3 vs 3.6±0.7 pg/mL; P<0.001) and cFn (11.4±6.5 vs 8.0±1.1 µg/mL; P = 0.001).  •Between G1 and G2: G1 ↑ levels of PTX3 (1455.4±487.5 vs 486.3±286.3 pg/mL; P<0.001), s TWEAK (212.0±213.3 vs 23.5±11.2 pg/mL; P= 0.03), CGRP (133.1±86.6 vs 26.9±12.5 ng/mL; P< 0.001, cFn (13.3±5.5 vs 8.0±1.1 µg/mL; P < 0.001).  G1 ↓ levels in IL-10 (1.5±1.7 vs 6.9±13.3 pg/mL; P = 0.011)  •Serum baseline levels with good response to BoNTA:  PTX3 >1000 pg/mL (AUC 0.908; 95% CI: 0.827-0.990)  CGRP >50 ng/mL (AUC 0.800; 95% CI: 0.652-0.947)  •↑CGRP and PTX3 reliable biomarkers for the selection of BoNTA in CM.  •Efficacy evaluation: diaries completed in the 3 months following BoNTA second dose. |
|  | LIMITATIONS: blood samples were only collected before BoNTA treatment. Relatively small samples from a selected clinical population. For ethical reasons, continued medications may serve as cofounders, and drug overuse was not assessed. Gender variations in CGRP levels were not accessed | | | |
| Cernuda-Morollón, 2014 | (n=114)  Follow-up:?  BoNTA  PREEMPT protocol (155-195U,31-39 injections) every 12 weeks, at least 2 cycles  G1. CM (n=81) (77F,4M)  G2. HG (n=33) (33F)  Prospective study LOE-III | Chronic migraine | **Plasma levels**  **Neuropeptide** – CGRP, VIP  determined using commercial ELISA kits. | •(n=61/75.3%) responders, (n=20/24.7%) non-responders.  •Responders: (n=41/50.6%) moderate response, (n=20/24.7%) excellent response.  •Blood samples only collected at baseline:  CGRP levels significantly ↑ in G1 (64.9 ± 31.0 pg/mL; range 11.4-157.7) vs G2 (33.3 ± 15.7 pg/mL; range 15.5-70.8; P < 10−10).  VIP levels significantly ↑ in G1 (173.7 ± 150.7 pg/mL; range 20.6-866.6) vs G2 (88.5 ± 62.3 pg/mL; range 15.5 ± 256.1; P < .001)  •CGRP levels significantly ↑ in responders (70.4 ± 31.9 pg/mL; range 12.8- 157.7;P < .005) vs non-responders (48.3 ± 21.2 pg/mL; range 11.4-110.8; P25 = 37.51, P50 = 45.03, P75 = 61.62). non-responders still significantly ↑ (P < .001) than G2.  •CGRP levels in moderate responders (66.1 ± 28.9 pg/mL; range 12.8-158.4; P25 = 42.88, P50 = 67.03, P75 = 85.48) numerically ↓ than excellent responders (79.2 ± 36.6, range 22.0-157.7; P25 = 48.27, P50 = 83.14, P75 = 95.28, P = NS).  •VIP levels, to a lesser degree, ↑ in responders (189.7 ± 162.3 pg/mL; range 20.6-866.6; P < .05) vs non-responders (115.5 ± 76.2 pg/mL; range 29.1-236.4; P25 = 53.23, P50 = 80.25, P75 = 197.31). non-responders did not differ from G2.  •VIP levels in moderate responders (160.5 ± 120.9 pg/mL, range 20.6–534.0; P25 = 81.52, P50 = 126.69, P75 = 213.99) numerically ↓ than excellent responders (245.3 ± 213.6 pg/mL; range 54.0- 866.6; P25 = 78.88, P50 = 202.08, P75 = 309.28, P = NS).  •CGRP - a threshold of 72 pg/mL positively correlated with 95% of non-responders. The probability of being a responder to BoNTA was 28 times higher in patients with a CGRP level above the threshold of 72 pg/mL.  •VIP - sensitivity for the calculated threshold for VIP was poor, but the probability that G1 with low CGRP levels will respond to BoNTA was significantly ↑ in those patients with ↑ VIP levels. •Interictal CGRP and, to a lesser degree, VIP levels in peripheral blood are reliable markers for CM diagnosis and in predicting response to BoNTA. |
|  | LIMITATIONS: unbalanced, small sample size, and a selected clinical population. Unknown true specificity of the increases in neuropeptides. Despite both patients and neurologists were blinded to the results of laboratory determinations when evaluating the efficacy of BoNTA, blood samples were only collected before treatment, and hence it could not be confirmed a correlation between clinical response and a decrease in the levels of these neuropeptides. One potential confounder (ethical reasons): oral preventatives. Unclear when was the follow-up/clinical assessments of efficacy. | | | |
| Moreno-Mayordomo, 2019 | (n=156) Caucasian Spanish females  Age 43.7±11.8ys (16-74)  BoNTA (PREEMPT protocol)  Follow-up 3 months after 2^nd^ procedure.  G1. Responders (reduction of at least 50% in the number of migraine days after two BoNTA procedures)  G2. Non-responders  Prospective, observational, multicenter clinical study – LOE-III | Chronic migraine | **Peripheral blood (genomic DNA)**  **Genes and 25 single nucleotide polymorphisms (SNPs):**  **Genes related to glutamate homeostasis –** MEF2D, LRP1, MTDH, EAAT2, GRIK3  **Gene which encodes for CGRP** – CALCA  **Genes of GABA system** – GABRE, GABRQ, GABRA3  **Genes which encode voltage dependent channels** – SCN9A, KCNS1, P2RX7  **Gene which encodes D2dopamine receptor** – DRD2(ANKK1)  **Gene of 5-HT2C serotonin receptor** – HTR2C  **Genes of the TRP family** – TRPV1, TRPV3, TRPM8  **Other genes** – WFS1, TGFBR2, MTHFR  (by genotyping using KASP probes and LightCycler-480 (Roche-Diagnostics)  Allelic, genotypic, frequencies and dominance/resistivity hypothesis of allelic variants (by Fisher´s exact test) | •Response to treatment with BoNTA was achieved in 120 patients (76,9%).  •Two polymorphisms showed differences:  **CALCA SNPrs3781719**, where allele C represents 26.9% in G1 and 40.9% in G2 (p=0.007, OR=3.11 (1.33–7.26));  **TRPV1 SNPrs222749**, where allele A represents 4.17% in G1 and 12.5% in G2 (p=0.013, OR=3.29 (1.28–8.43)).  •No significant differences in rest of polymorphisms or clinical or demographic variables were found. •Polymorphic variations of CALCA and TRPV1 genes might play a role as prognostic markers of efficacy of BoNTA in chronic migraine female patients. |
|  | LIMITATIONS: Did not consider if the presence of sympathetic or parasympathetic symptoms correlated with the genotype or if other parameters influenced the response. | | | |
| Cutrer, 2006 | (n=1) F , 34ys  5 years of numerous treatments for her scalp pain and hair loss without sustain benefit- maximally tolerated daily doses of buspirone (55 mg), propranolol (180 mg), and tizanidine (16 mg), indomethacin (225 mg), nortriptyline (50 mg), amitriptyline (50 mg) and gabapentin (2700 mg), Oral opiates (propoxyphene, codeine and hydrocodone), midodrine (5 mg).  BoNTA (100 units total), repeated at 3-months and 5-months  Case report study – LOE-V | Cephalalgia - alopecia areata | **Brain / Scalp/ Blood**  MRI/angiography (brain)  Biopsy (scalp) | • Baseline:  MRI/angiography of brain, showed that the lateral ventricles were near the upper limit of normal.  Biopsy of the affected scalp showed lymphocytic infiltration around the hair bulb characteristic of alopecia areata.  Thyroid stimulating hormone, free T4, α-androstanediol and glutamate levels were normal. Blood testing for inflammatory markers including erythrocyte sedimentation rate (ESR), rheumatoid factor (RF), antinuclear antibody (ANA) and antibodies to extractable nuclear antigens (ENA) was negative.  • BoNTA resulted in marked ↓in the frequency and intensity of the jabbing head and neck pain, partial hair regrowth which began approximately 10 days after injection and lasted 6 weeks. No improvement was seen in the myalgias and fatigue and she began to experience muscle spasm in the intrascapular and thoracic areas of her back.  •at 3-months (2^nd^ session), complete remission of the back pain for 45 days and the head pain for 60 days, Significant hair regrowth.  •By the eighth week she began to experience recurrence of the neck and head pain and loss of hair. A 3^rd^ session of BoNTA treatment has resulted in a similar remission. |
|  | LIMITATIONS: Case report. Biopsy and blood samples were only taken at baseline for diagnostic purposes (Diagnostic biomarkers) | | | |
| **LEGEND**  **AOPP**, advanced oxidation protein products; **BDNF**, brain-derived neurotrophic factor; **BoNTA**, botulinum toxin type A; **CGRP**, calcitonin gene-related peptide; **CIS**, chronic inflammatory state; **CRP**, C-reactive protein; **LOE**, level of evidence; **G**, group; **↑**, higher; **↓**lower; **FRAP**, ferric reducing antioxidant power; **SH**, thiolic groups; **CM**, chronic migraine; **IHS**, international headache society; **HG**, healthy group; **IC**, interstitial cystitis; **BPS**, bladder pain syndrome; **TN**, trigeminal neuralgia; **SP**, substance-P; **VIP**, vasoactive intestinal peptide; **[IQR]**, interquartile range; **NGF**, nerve growth factor; **MS**, multiple sclerosis; **NLUTD**, neurogenic lower urinary tract dysfunction; **IL**, interleukin; **TNF-α**, tumor necrosis factor alfa; **TGF-β1,** transforming growth factor β1; **sTWEAK**, tumor necrosis factor weak inducer of apoptosis in its soluble form; **MCP-1**, monocyte chemoattractant protein-1; **PTX3**, pentraxin 3; **PASI**, Psoriasis Area and Severity Index; **PGA**, Physician's Global Assessment; **ENF**, epidermal nerve fibre; **SNP**, subepidermal neural plexus; **RT-PCR**, real-time polymerase chain reaction; **VAS**, visual analog scale; **QoL**, quality of life; **IPSS**, International Prostate Symptom Score; **AUA**, American Urological Association score; **TRUST**, transrectal ultrasonography; **LUTS**, lower urinary tract symptoms; **BPH**, benign prostatic hyperplasia; **TPV**, total prostate volume; **Qmax**, maximal flow rate; **CP/CPPS**, refractory chronic prostatitis-associated chronic pelvic-pain syndrome; **WBC**, white blood cell; **EPS**, expressed prostatic secretion; **IIEF-5**, International Index of Erectile Function-5; **SNPs**, single nucleotide polymorphisms; **TRPV1**, Transient Receptor Potential Vanilloid 1; **MMP**, matrix metalloproteinase; | | | | |
