# Supplementary material for: Botulinum toxin effects on biochemical biomarkers related to inflammation-associated head and neck chronic conditions: a systematic review of clinical research
Source: J Neural Transm (Vienna). 2025 Mar 4;132(12):1851–74. doi: 10.1007/s00702-024-02869-w (PMC12669376; doi:10.1007/s00702-024-02869-w)
Supplement: Supplementary file 6 — Supplementary file6 (DOCX 29 KB) [file 702_2024_2869_MOESM6_ESM.docx]

**Supplementary Information 4. Table 3:** Biomarkers in Clinical Research on Botulinum Toxin effects on Chronic Inflammatory State. Ex vivo and in vitro studies - cultured cells-derived from humans with the condition.

| **CLINICAL STUDIES** | | | | |
| --- | --- | --- | --- | --- |
| **Author, Year** | **Study Design Population**  **LOE** | **Condition**  **CIS** | **Biological sampling Biomarker** | **BoNTA**  **Key effect**  **Key mechanism** |
| **INFLAMMATION-NEUROGENIC INFLAMMATION-NEUROINFLAMMATION** | | | | |
| Shon, 2020  * Also in vitro  Letter to the Editor LOE - V | (n=18) male mean age of 49.00±6.50ys  Follow-up: 12, 24 weeks  *Cultured DPCs (from balding scalps) under 10^-9^mol/L Dihydrotestosterone  Follow-up: 24h, 96h  BoNTA (total:30U) intradermal injections into 20 different sites on the balding scalp every 4 weeks for 24 weeks.  * BoNTA (2.5 U/10^6^ cells) | Androgenic Alopecia | **Balding Scalp *hair bulb -cultured Dermal papilla cells (DPCs) ***  Transforming growth factor β1 (TGF-β1)  (evaluated by RT-PCR)  Other assessment: phototrichogram image analysis | •Mean ± standard deviation of hairs per square centimetre at weeks 0, 12, and 24: 129.61 ± 28.05, 129.11 ± 28.80, and 136.22 ± 33.05, respectively.  •The number of hairs significantly ↑ at week 24 (*P* = .012) but not at week 12 (*P* = .803).  •Comparison of the pre- and posttreatment photographs showed significant improvement at week 24 (*P* = .031).  •Dihydrotestosterone ↑ TGF-β1 expression of DPCs in 96 hours, whereas BTX ↓ TGF-β1 expression in 96 hours.*  •No serious adverse events or changes in laboratory parameters were reported. |
|  | LIMITATIONS: Finding being obtained only in DPCs cultured in vitro. The in vitro environment may cause some limitations of the experimental results. Cultured cells treated with BoNTA | | | |
| Park, 2019  * Also in vitro | (n=11) human derived fibroblasts in vitro  Follow-up: 0,12,24,48h  G1. BoNTA* (cells treated with 8 units/mL)  G2. Control (untreated) | Hypertrophic scar for at least 1 year | **Fibroblasts-derived hypertrophic scar**  Proliferation  (with colorimetric)  Migration  (by scratch)  **protein expression of pro-fibrotic factors and scar-related EMC** -TGF-β1, IL-6, hPro-collagen Iα1, connective tissue growth factor  **JNK signalling pathway**  (by ELISA and western blotting) | •G1 significantly ↓ proliferation compared with G2 (after 48h (P < 0.01), but not at 24h (P > 0.05)).  •G1 ↓ migration of human scar fibroblasts compared with G2. This was most effective between 12 and 24h: areas filled with migrated HSFs - at 12h, 32.3% (G2),8.9% (G1); at 24h, 62.1%(G2), 13.7% (G1); at 48h, 97.3%(G2), 77.3%(G1)  •Protein expression levels of pro-fibrotic factors were also significantly ↓ in G1.  •JNK phosphorylation level was ↑ in G1 compared to G2, in vitro: maximum at 4h (3.1-fold), then JNK activity ↓at 8h (1.1-fold) but recovered at 24h ((3.1-fold) and was maintained at a high level (2.3-fold) until 48h.  •Activation of the JNK pathway demonstrated the inhibitory effects of BoNTA on human scar fibroblast proliferation and production of pro-fibrotic factors, suggesting that the suppressive effects of BoNTA are closely associated with JNK phosphorylation.  •Overall, this study showed that BoNTA has a suppressive effect on extracellular matrix production and scar-related factors in human scar fibroblasts in vitro, and that regulation of JNK signalling plays an important role in this process. |
|  | LIMITATIONS: Unknown location of HS (Head and Neck?) Findings obtained only in fibroblasts cultured in vitro. The in vitro environment may cause some limitations of the experimental results. Cultured cells treated with BoNTA | | | |
| Hao, 2018  * Also in vitro | (n=?) human hypertrophic scars derived-keloid fibroblasts, explant technique  Follow-up:  G1. Control + normal fibroblast  G2. Control + keloid fibroblasts  G3. Different BoNTA doses for 24h: 0.01U/L, 0.1U/L, 1U/L, 10U/L (normal or keloid fibroblasts) | Hypertrophic scarring | **Fibroblasts-derived keloids**  Cellular morphology, viability, proliferation, cell cycle, and apoptosis (by immunofluorescence, MTT assay, flow cytometry)  **protein expression of main factors influencing collagen degradation of keloid tissue** -TGF-β1, MMP-1, MMP-2, MMP-9  (by RT qPCR, western blotting) | •BoNTA significantly influences the viability and shape of keloid fibroblasts: Keloid fibroblast viability ↓ with increasing BoNTA dose. The keloid fibroblast spreading area become smaller, there was fragmentation, and number of adherent cells significantly ↓.  •After BoNTA treatment, the volume of keloid fibroblasts cells ↑, but the nucleus of cells shrunk. Long thin dendrites were formed as the concentration of BoNTA ↑.  •BoNTA promotes apoptosis and inhibits proliferation of keloid fibroblasts. The proliferation and S phase of keloid fibroblasts were ↓ by BoNTA (n=6, **P<0.01 vs control).  •Matrix metalloproteinase (MMP)-1 and -2 RNA (genes) and protein showed ↑ expression, but TGF-b1 and MMP-9 showed statistically significant ↓ expression than the control (n=5, *P<0.05 vs Control, **P<0.01 vs Control).  •The inhibitory effect of BoNTA on keloid fibroblasts only occurred when the concentration was ↑ than 0.1 U/L.  •Compared with keloid fibroblasts, BoNTA had no significant inhibitory effect on normal fibroblasts, even when the concentration was ↑ to 10 U/L. •BoNTA may promote the healing of scars by inhibiting the proliferation of keloid fibroblasts and regulating the expression of TGF-b1, which could affect the expression of MMP-1 and MMP-2. |
|  | LIMITATIONS: Finding being obtained only in fibroblasts cultured in vitro. BoNTA treated cultured cells. The in vitro environment may cause some limitations of the experimental results. Unknown location of HS | | | |
| Jeong, 2015  * Also in vitro | (n=9) patients, (n=10) tissue specimens derived from scars:  5 - normal mature scars (pale, flat),  3M, 2F;  5 - hypertrophic scars (red, protruded), 3M, 2F  Follow-up: 0, 24, 48, 72h (cellular proliferation)  Pre-treated with TGF-β1 to induce differentiation:  G1. Control (treated with TGF-β1 only)  G2. BoNTA (4U/ml)* | Hypertrophic scars (more than 2 years after suture of the wound from surgery or trauma) – neck, knee, lower abdomen, cheek | **Cultured fibroblasts-derived tissue specimens**  **Gene expression of myofibroblast marker α-SMA**  (enzyme-linked immunosorbent assay, quantitative reverse transcription polymerase chain reaction) **Fibroblast-to-myofibroblast differentiation**  (immunocytochemistry, confocal microscopy) | •α-smooth muscle actin mRNA levels were significantly ↓ in G2 than G1 of fibroblasts derived from hypertrophic scars (9.40±0.81 vs 7.47±2.00; P=0.038), but not fibroblasts derived from normal scars (9.97±2.91 vs 8.3±2.39; P=0.207).  •α-smooth muscle actin protein levels were significantly ↓ in G2 than G1 of fibroblasts derived from hypertrophic scars (2.46±0.38 vs 2.03±0.30; P=0.028), but not fibroblasts derived from normal scars (1.81±0.65 vs 1.47±0.44; P=0.086).  •Proliferation was significantly ↓ after BoNTA in fibroblasts derived from both mature scar tissue and hypertrophic scars (P<0.001).  •Immunocytochemistry results also showed that fibroblast-to-myofibroblast differentiation was significantly ↓ after BoNTA in fibroblasts derived from hypertrophic scars (P=0.012), but not in fibroblasts derived from normal scar tissue (P=0.093).  •BoNTA directly inhibits fibroblast-to-myofibroblast differentiation in vitro, and indicate its potential for use in treating wounds expected to develop into hypertrophic scars after trauma, burn, or surgery. |
|  | LIMITATIONS: Finding being obtained in fibroblasts cultured in vitro. BoNTA treated cultured cells. The in vitro environment may cause some limitations of the experimental results. | | | |
| Xiaoxue, 2014  * Also in vitro | (n=12) Northeast China patients – samples keloid lesions  G1. 3 Experimental BoNTA (1μL/10⁶ cells and 2.5μL/10⁶ cells)  G2. 3 Control (no BoNTA)  G1/2a (for 24h), G1/2b (48h), G1/2c (72h) | Keloid scarring | **Fibroblast-derived keloids**  112 genes (S100A4, TGF-β1, VEGF, MMP-1, and PDGFA) relevant to invasive growth in keloid fibroblasts  (microarray analysis to study messenger RNA expression profiles (qRT-PCR) | •BoNT-A 2.5μL/10⁶ for 48h the ideal condition for 50% ↓ of the number of viable keloid fibroblasts.  •S100A4 gene was significantly upregulated (4.01-fold) and TGF-β1, VEGF, MMP-1, and PDGFA genes were significantly downregulated in fibroblasts treated with BoNTA (3.73-, 3.65-, 3.12-, and 2.68-fold, respectively). |
|  | LIMITATIONS: unknown the location of keloids, keloid characteristics. Finding being obtained only in fibroblasts cultured in vitro. The in vitro environment may cause some limitations of the experimental results. | | | |
| Gauglitz, 2012  * Also in vitro | (n=4) 2F, 2M  Isolated primary fibroblasts from keloid patients (n=3)  BoNTA 0-140 Speywood U/session injected into keloids every 2-months for 6-months. | Keloid scars longer than 2 years and resistant to therapy. | **Keloid-derived fibroblasts**  **Extracellular matrix (ECM) markers -** fibronectin-1, laminin-β2, α-SMA.  (by RT-PCR)  **Markers for collagen synthesis/expression:** (COL)1A1, COL1A2, COL3A1, TGF-β1, TGF-β2, TGF-β3.  **Fibroblast proliferation and metabolism** (by MTT and BrdU assays) | •Intralesional administration of BoNTA did not result in regression of keloid tissue.  •No differences in expression of ECM markers, collagen synthesis, or TGF-β could be observed after BoNTA treatment of keloid fibroblasts.  •Cell proliferation and metabolism of keloid fibroblasts was not affected by BoNTA.  Other assessments: 3-D optical profiling to measure the differences in height of the keloid scars. |
|  | LIMITATIONS: unknown the location of keloids and treatment protocol according to injection site, keloid characteristics. Finding being obtained only in fibroblasts cultured in vitro. The in vitro environment may cause some limitations of the experimental results. | | | |
| Xiao, 2011  * Also in vitro | (n=8)  Follow-up: 1^st^ to 5^th^ day  G1. Fibroblasts treated with BoNTA (1 U/10^6^ cells, 2.5 U/10^6^ cells)  G2. Fibroblasts without BoNTA | Hypertrophic scar | **Fibroblasts Derived from Hypertrophic Scar**  **Downstream regulator of TGF-β1 function and independent mediator of scarring and fibrosis** - Proteins of connective tissue growth factor – CTGF  (by Western blot)  **Fibroblast proliferation**  (by flow cytometry) | •The proliferation in G1 was slower than G2 (p<0.01), which showed that BoNTA effectively ↓ the growth of fibroblasts.  PROLIFERATION:  G2 (phase g0-g1, 34.21 ± 2.05; phaseS, 19.18 ± 1.93, phase g2-M, 47.17 ± 1.85)  G1 (1 U/10^6^ cells) (phase g0-g1, 58.14 ± 1.64; phaseS, 8.67 ± 1.43, phase g2-M, 34.65 ± 1.37; p<0.01 compared with G2)  G1 (2.5 U/10^6^ cells) (phase g0-g1, 61.12 ± 1.45; phaseS, 9.34 ± 1.57, phase g2-M, 30.46 ± 1.25; p<0.01 compared with G2)  •Compared with G2, BoNTA at 1 U/10⁶ cells ↓ the expression of CTGF by 49.2% ± 12.5% (p < 0.01), and BoNTA at 2.5 U/10⁶ cells ↓ the expression of CTGF by 56.9% (p < 0.01). No significant reduction in CTGF was found between G1 of 1.0 U/10⁶ and G1 of 2.5 U/10⁶ cells (p >0.05) |
|  | LIMITATIONS: it could not be ascertained the reason why BoNTA could regulate the expression of the cytokine in fibroblasts derived from hypertrophic scar. It could not be defined whether the BoNTA effect on CTGF was specific to hypertrophic scar-derived fibroblasts. Finding being obtained only in fibroblasts cultured in vitro. The in vitro environment may cause some limitations of the experimental results. | | | |
| Xiao, 2010  * Also in vitro | (n=8)  Follow-up: 1^st^ to 5^th^ day  G1. Fibroblasts treated with BoNTA (1 U/10^6^ cells, 2.5 U/10^6^ cells)  G2. Fibroblasts without BoNTA | Hypertrophic scar | **Fibroblasts Derived from Hypertrophic Scar**  Proteins of TGF-β1  **Fibroblast proliferation**  (by MTT assay) | •The growth in G1 was slower than G2 (*p*<0.01)  •At G1, fibroblast proliferation indicated a static population - G1 effectively inhibited the growth of fibroblasts.  •Proteins of TGF-β1 between G1 and G2 were statistically significant (*p*<0.01). |
|  | LIMITATIONS: unknown the location of hypertrophic scars and treatment protocol according to injection site, scars characteristics. Finding being obtained only in fibroblasts cultured in vitro. The in vitro environment may cause some limitations of the experimental results. | | | |
| Zhang, 2022  * Also in vitro and animal study | Chinese and age: 9 to 43ys  Human fibroblasts of normal skin and hypertrophic scars:  (n=6) normal skin samples, (n=8) hypertrophic scar samples.  G1. BoNTA (4 U/mL or 10 U/mL)  G1a. NK-1R antagonist aprepitant (5μM)  G2. Capsaicin (25 μM/L) – SP inducing agent  G3. BoNTA + capsaicin  G4. Control (no treatment) | Hypertrophic scar | **Human skin- hypertrophic scars-derived fibroblasts**  Expression of SP, PCNA  (immunohistochemistry, RT-PCR, western blot)  **Collagen expression** - SP-NK1R pathway (signalling pathways in Cutaneous neurogenic inflammation) | •G1 (4 U/mL or 10 U/mL) significantly ↓ *SP* expression. Even with the presence of capsaicin (SP inducing agent), the expression of *SP* remained inhibited by BoNTA.  •G1 without induction of SP, type 1 collagen presented a significant ↓ with elevated doses of BoNTA. •Both low-dose and high-dose BoNTA successfully ↓ the overexpression of type 1 collagen induced by SP (*p* < 0.0001). This effect remained in the presence of the SP inducing agent capsaicin.  •Expression of type 1 collagen was significantly ↓ by G1 (4 or 10 U/mL). However, G1a, the expression of type 1 collagen showed no significant difference with G4 (G1a/G4, *p* = 0.1856 and BoNTA-10/aprepitant, *p* = 0.1634) – It was concluded that SP-NK1R pathway mediates BoNTA to ↓ collagen expression in hypertrophic scars. |
|  | LIMITATIONS: Specific SP antagonist treatment is needed to verify the effect of SP on fibroblast proliferation. The binding of BoNTA with SP or NK-1R has yet to be discovered. Apart from the SP-NK1R pathway, other possible mechanisms of action of BoNTA in inhibiting hypertrophic scars need to be explored, the interactions between SP-NK1R and the other pathways remain unclear. | | | |
| **LEGEND**  **LOE**, level of evidence; **CIS**, chronic inflammatory state; **BoNTA**, botulinum toxin type A; **G**, group; **↑**, higher; **↓**lower; **AOPP**, advanced oxidation protein products; **FRAP**, ferric reducing antioxidant power; **SH**, thiolic groups; **CM**, chronic migraine; **IHS**, international headache society; **HG**, healthy group; **IC**, interstitial cystitis; **BPS**, bladder pain syndrome; **TN**, trigeminal neuralgia; **CGRP**, calcitonin gene-related peptide; **SP**, substance-P; **VIP**, vasoactive intestinal peptide; **[IQR]**, interquartile range; **BDNF**, brain-derived neurotrophic factor; **NGF**, nerve growth factor; **MS**, multiple sclerosis; **NLUTD**, neurogenic lower urinary tract dysfunction; **IL**, interleukin; **CRP**, C-reactive protein; **TNF-α**, tumor necrosis factor alfa; **TGF-β1,** transforming growth factor β1; **sTWEAK**, tumor necrosis factor weak inducer of apoptosis in its soluble form; **MCP-1**, monocyte chemoattractant protein-1; **PTX3**, pentraxin 3; **PASI**, Psoriasis Area and Severity Index; **PGA**, Physician's Global Assessment; **ENF**, epidermal nerve fibre; **SNP**, subepidermal neural plexus; **RT-PCR**, real-time polymerase chain reaction; **VAS**, visual analog scale; **QoL**, quality of life; **IPSS**, International Prostate Symptom Score; **AUA**, American Urological Association score; **TRUST**, transrectal ultrasonography; **LUTS**, lower urinary tract symptoms; **BPH**, benign prostatic hyperplasia; **TPV**, total prostate volume; **Qmax**, maximal flow rate; **CP/CPPS**, refractory chronic prostatitis-associated chronic pelvic-pain syndrome; **CPSI**, chronic prostatitis symptom index; **Qmax**, maximum urinary flow rate; **WBC**, white blood cell; **EPS**, expressed prostatic secretion; **IIEF-5**, International Index of Erectile Function-5; **SNPs**, single nucleotide polymorphisms; **TRPV1**, Transient Receptor Potential Vanilloid 1; **MMP**, matrix metalloproteinase; **α-SMA,** α-smooth muscle actin; | | | | |
