# Supplementary material for: Botulinum toxin effects on biochemical biomarkers related to inflammation-associated head and neck chronic conditions: a systematic review of clinical research
Source: J Neural Transm (Vienna). 2025 Mar 4;132(12):1851–74. doi: 10.1007/s00702-024-02869-w (PMC12669376; doi:10.1007/s00702-024-02869-w)
Supplement: Supplementary file 7 — Supplementary file7 (DOCX 52 KB) [file 702_2024_2869_MOESM7_ESM.docx]

**Supplementary Information 4. Table 4:** Biomarkers in Clinical Research on Botulinum Toxin effects on Chronic Inflammatory State. Nearly included studies – Excluded with reasons

| **CLINICAL STUDIES** | | | | | | |
| --- | --- | --- | --- | --- | --- | --- |
| **Author, Year** | **Study Design Population**  **LOE** | **Condition**  **CIS** | | | **Biological sampling Biomarker** | **BoNTA**  **Key effect**  **Key mechanism** |
| **Missing Primary data (although it reported biomarkers evaluating BoNTA effect)** | | | | | | |
| Kim, 2021 | (n=9, 8 completed), 1 excluded due to hemoglobin level of 9 g/dL (3M,5F)  Age: 46.3 ± 7.8ys  Follow-up: 2-, 4-, 12-weeks after BoNTA (20U) | | Unilateral intractable chronic occipital neuralgia | **Blood**  Other assessments: pain relief (VAS), Quality of life | | • pain relief was continuously observed from the 2nd week to 12th week after injection, showing statistical significance (*p* = 0.011, *F* = 125.49) and the most notable change was observed 2 weeks after injection.  • There was no significant change in the quality of life (QOL) evaluation throughout the study, and the participants did not experience adverse reactions.  • **VAS** (baseline:7.66 ± 0.70; 2-weeks:3.38 ± 2.50 ***; 4-weeks:1.13 ± 0.99 ***; 12-weeks:0.87 ± 0.64; F =125.49 ***) *** *p* = 0.000.  • **QOL** (baseline:76.67 ± 9.95; 2-weeks:70.88 ± 8.22; 4-weeks:70.38 ± 4.41; 12-weeks:72.0 ± 4.93; F=997.15)  • No significant differences in the posttreatment hematological results, which were similar to the serologic results obtained before BoNTA. |
|  | LIMITATIONS: missing data (primary outcome – serologic values) – contact protocol (^*^Correspondence: [Seong-Taek Kim](https://pubmed.ncbi.nlm.nih.gov/?term=Kim%20ST%5BAuthor%5D)- [k8756050@yuhs.ac) 1st](mailto:k8756050@yuhs.ac)%201st) attempt: 23^rd^ Oct 2023, 10.33am | | | | | |
| **CONDITION/LESIONS NOT in HEAD & NECK (although it reported biomarkers evaluating BoNTA effect)** | | | | | | |
| Todberg, 2018  ClinicalTrials.gov, ID:NCT02577185 | (n=10, n=8 completed; 1 missed visit 4, 1 was lost to follow-up after visit 6) 9M,1F, mean BMI: 26.9 (range 21–36), mean TCS: 5.2 (±0.3), mean age: 53.5 (range 22–77)  All randomized participants were included in the analyses - up to two target lesions with Total Plaque Score (TPS) of ≥4 (sum of erythema 0–3, scaling 0–3 and infiltration 0–3, range 0–9) and a scaling score ≤1 at randomization were selected. TPS was not allowed to differ more than 1 point if two plaques were selected. Topical or systemic therapy for psoriasis was not allowed.  Follow-up: 1,3,4,8-weeks (biopsy) after randomisation  Single dose of BoNTA 36U Dysport (nine injections of 4 units)  G1. BoNTA  G2. Placebo-sodium chloride  exploratory, multicenter, RCT double-blinded. LOE-II | | Plaque Psoriasis  (According to NCT02577185: Inclusion criteria - psoriasis vulgaris with lesions located on arms and/or legs and/or trunk) | | **Lesional & perilesional skin**  Histological information from skin biopsies  Through 3 biopsies-4mm diameter-2 weeks before, 8 weeks after injection + immunostaining.  Other assessments: Primary outcome - absolute change of Total Clinical Score (against placebo and baseline) - sum of erythema 0–3, scaling 0–3 and infiltration 0–3, range 0–9), individual clinical sign score, information on safety. | •No clinical or histopathological statistically differences between G1 and G2. However, due to lack of clinical effect, not all histological analyses were performed. It was not investigated whether repeated doses of BoNTA could influence results.  •Total Clinical Score (TCS) ±SD at week 0, 1, 3, 4 and 8. N = 10. Single dose of botulinum neurotoxin (BoNTA) led to no improvement in TCS score from baseline to week 8. Mean change in TCS from baseline to end of trial, for BoNTA -0.4, for vehicle -0.25 (95% CI, -0.49 to 0.19, P = 0.34).  •Premature ending due to lack of efficacy based on 8-week blinded data obtained from 8 subjects.  •No serious adverse-effects reported, 5 adverse-effects reported, and none were judged to be related to the trial medication. |
|  | LIMITATIONS: small sample size - One subject missed visit 4, and one subject was lost to follow-up after visit 6. It was not investigated whether repeated doses of BoNTA could influence results. Funded by Pharmaceuticals. Discrepancies between trial register and published results regarding intended outcomes (i.e., histological examination was not initially planned, only Single masking – Investigator vs double-blind published results). Histological information based on 1 subject, only?  Correspondence: T. Todberg. E-mail: [tanja.todberg@regionh.dk](mailto:tanja.todberg@regionh.dk), 1^st^ attempt: 23^rd^ Oct 2023, 10.53am | | | | | |
| Aschenbeck, 2018 | (n=12, n=8 completed) 6M,2F  1-time injection BoNTA (Botox) throughout a single target plaque (average:53U; range:25-98U)  Follow up: for 10 weeks  Single-centre Pilot Study LOE-IV | Plaque Psoriasis  (Elbow, back, knee, leg, foot) | | | **Lesional & perilesional skin**  **Epidermal density** - ENFs  **Neuropeptides -** SP and CGRP  Through punch biopsy-3mm diameter-2 weeks before, 8 weeks after injection + immunostaining  Other assessments: Psoriasis Area and Severity Index (PASI) score and Physician's Global Assessment (PGA) score and clinical photography. | •After BoNTA, SP (*P*=.061) and CGRP (*P*=.053) immunoreactivity ↓ in the lesional and perilesional samples. ENF density (*P* = .10) increased. BoNTA associated with significantly ↓ PASI and PGA scores (*P* < .01).  •No serious adverse events or adverse effects. None of the subjects discontinued participation secondary to side effects.  •BoNTA clinical effect of ↓ plaque severity may be mediated by ↓ SP- and CGRP-immunoreactive nerve expression and ↑ ENF density. |
|  | LIMITATION: small sample - 40 screened, 28 excluded with reasons, 2 lost to follow-up (unable to be contacted), 2 withdrew; predominance of males, lack of a placebo control, cost of BoNTA, and variance in body site of target plaque and average ENF and neuropeptide densities by body site. | | | | | |
| Philippova, 2021 | (n=56)  Follow-up:  1,3,6-months  BoNTA 200U intradetrusor injections  G1. MS, NLUTD  (n=36) (25F,11M)  G2. HG (n=20) (10F, 10M)  Prospective cohort LOE-III | Multiple sclerosis (MS) and neurogenic lower urinary tract dysfunction (NLUTD) | | | **Serum & Urine levels**  **Neurotrophic factors** – BDNF, NGF  measured by enzyme-linked immunosorbent assay. Urinary NGF and BDNF were normalized to creatinine. | •G1 baseline (compared to G2)  Urine  NGF - G1 (1.23 ± 0.34) significantly ↑ than G2 (0.084 ± 0.02; p = .021).  •G1 follow-up  Serum - NGF and BDNF no significant changes  Urine  BDNF no significant changes  NGF:  1 month ↓ to 0.51 ± 0.12 (p = .001)  3 months ↓ 0.53 ± 0.32 (p = .005)  6 months ↑ to 1.12 ± 0.49 (p = .003)  •Urinary NGF at baseline or its reduction at the first month following BoNTA has low diagnostic accuracy in predicting a better response and no correlation with the urodynamic parameters.  Other measurements: urodynamic examination and Neurogenic Bladder Symptom Score. |
|  | LIMITATIONS: Lack of MS in G2. Fluctuations in NGF and BDNF levels connected with MS pathophysiology may have affected the results. G1 and G2 different in gender and age parameters that may have influence the results. | | | | | |
| Khatery, 2022 | (n=20) 15F, 5M Age:13-34 (mean: 19.9 ± 5.32)  (n=12) keloids, (n=8) hypertrophic scars  BoNTA injection intralesional (1 session/month) for three months.  2.5 per cubic centimetre of lesion in each.  Follow-up: 3-, 6-, 9-months  Two biopsies - baseline (0) and from the scar 1 month after the last treatment session (adjacent to the pretreatment biopsy site). | Keloids and hypertrophic scars  (Upper limb (n=14; 70%), trunk (n=4; 20%), lower limb (n=2; 10%) | | | Histologic grading scores (changes in the quality of collagen and elastic tissues)  Image analysis (quantitative morphometric changes)  Other clinical assessment: Vancouver Scar Scale (VSS), Observer Scar Assessment Scale (OSAS), and the Patient Scar Assessment Scale (PSAS). | • ↑ statistically significant difference between baseline and the result after each of the 3 sessions of injection and 3, 6 months after the last session regarding VSS, OSAS, and PSAS (p ≤0.001).  • Statistically significant difference between the histopathologic findings before injection of BoNTA and 1 month after the 3^rd^ session regarding all parameters used:  ↓ collagen area% (quantity) after BoNTA (mean from 22.7747 ± 8.96273 (baseline) to 18.5968 ± 6.36161 (1 month after the last session); ↓ collagen grading score for collagen quality (mean value of the score of collagen fibers from 3.15 ± 1.461 (baseline) to 2.75 ± 1.482 (1 month after the last session) that indicates improvement; ↑ elastic tissue area% (quantity) after BoNTA (mean from 6.7562 ± 4.17807 (baseline) to 11.2532 ± 5.41156 (1 month after the last session); ↓ Orcein stain grading score for elastic tissue quality (mean from 3.45 ± 1.395 (baseline) to 3.20 ± 1.542 (1 month after the last session) which means better outcome. • No statistically significant relations between percent change over time in VSS from baseline till 1 month after the last session in the histologic parameters examined in hypertrophic scars and keloids separately except for collagen area percent (quantitative) in hypertrophic scar that shows a significant relation (p = 0.017) and a positive correlation (r = 0.802).  • BoNTA can be a good therapeutic manoeuvre for management of keloid and hypertrophic scars with significant clinical and histologic improvement. |
| **HEALTHY INDIVIDUALS - EXPERIMENTALLY INDUCED-DISEASE** | | | | | | |
| Gazerani, 2008 | (n=14) healthy males (23-32ys, 26.3+/-2.6 years)  Pain and neurogenic inflammation induced by 4 intradermal injections of capsaicin (100mug/muL) (before, and days 1, 3 and 7 after treatments)  Blood flow taken prior and after capsaicin injection.  Subcutaneous electromyographic (EMG)-guided injection in the mirror sides of the forehead  G1. BoNTA (22.5U)  G2. isotonic saline  Randomised double-blind and placebo-controlled trial LOE-II | capsaicin-induced trigeminal pain, neurogenic inflammation and experimentally induced cutaneous pain modalities. | | | Blood flow (using laser Doppler imaging system – output signal proportional to blood cell perfusion (or flux)  Other assessments: pain intensity and duration (VAS), pain area, area of secondary hyperalgesia, area of visible flare and vasomotor reactions, cutaneous heat (thermographic images), electrical and pressure pain thresholds. | • The earliest analgesic effect of BoNTA recorded at 24h after injection.  • G1 ↓ capsaicin-induced trigeminal pain intensity compared to G2 (F=37.9, P<0.001).  • The perceived pain area was smaller for G1-treated side compared to G2 (F=7.8, P<0.05).  • G1 ↓ capsaicin-induced secondary hyperalgesia (F=5.3, P<0.05) and flare area (F=10.3, P<0.01) compared to G2.  •G1 ↓ blood flow (F(1,26)=109.5, P<0.001) and skin temperature (F(1,26)=63.1, P<0.001) at the capsaicin injection sites compared to G2 and its suppressive effect was maximal at days 3 and 7 (P<0.05, post hoc test).  •G1 ↑ cutaneous heat pain thresholds (F=17.1, P<0.001) compared to G2; however, no alteration was recorded for electrical or pressure pain thresholds (P>0.05).  •The study suggest that BoNTA appears to preferentially target Cfibers and probably TRPV1-receptors, block neurotransmitter release and subsequently reduce pain, neurogenic inflammation, and cutaneous heat pain threshold. |
| da Silva, 2014 | (n=) healthy subjects  pre-BoNTA session - injection of glutamate (1M, 0.2mL) and saline (0.2mL) into the temporalis muscles  On day 1, injection into temporalis muscle:  G1- BoNTA (5U) treated side  G2 – saline  Following intramuscular injections of glutamate (1M, 0.2mL) into the left and right temporalis muscles at 3h and 7, 30 and 60 days post-injection of BoNTA.  Randomised, split face, clinical trial LOE-II | Pain and vasomotor responses evoked by glutamate injection into human temporalis muscles – model of persistent myofascial TMD pain | | | Pain intensity, pain area, and neurogenic inflammation (skin temperature and skin blood perfusion) | • Prior to BoNTA, glutamate evoked significantly greater pain and vasomotor reactions (*P* < 0.001) than saline.  • G1 significantly ↓ glutamate-evoked pain intensity (*P* < 0.05), pain area (*P* < 0.01), skin blood perfusion (*P* < 0.05), and skin temperature (*P* < 0.001).  • The inhibitory effect of BoNTA was present at 3h after injection, peaked after 7 days and returned to baseline by 60 days.  • rapid action of BoNTA on glutamate-evoked pain and neurogenic inflammation, which is in line with animal studies. |
| **(BIO) FUNCTIONAL MEASUREMENTS** - possibly clinical outcome assessments (COAs) that are directly important to the patients rather than biomarkers that serve to link a measurement to a prediction of COAs | | | | | | |
| Reyes, 2023 | (n=12), 10 completed (1 had a post BoNTA fMRI but no follow-up clinic visit, 1 did not fill out questionnaires during their second clinic visit).  Age:53.5±9.6 4M,8F  event-related fMRI design, subjects were presented with light stimuli during two fMRI scans (before/after BoNTA).  Follow-up: 4-,6-weeks after BoNTA  BoNTA at 7 sites on the forehead, total of 35U: 5Uprocerus, 10Ucorrugators, 20Ufrontalis.  G1. (n=6) Responders - ↓unpleasantness ratings in response to light stimulation.  G2. (n=6) Non-responders; (n=3) had equivalent or (*n* = 3) ↑ reported unpleasantness with light stimulation.  Cohort study – LOE-III | Chronic (≥3 months) ocular pain over 1 week recall and photophobia (Neuropathic Pain Symptom Inventory modified for the Eye, question #9 ≥ 1) | | | **whole brain blood** oxygen level dependent (BOLD) responses to light stimuli  (measured with  Functional magnetic resonance imaging (fMRI)  Other assessments: clinical exam – 1.collected tear parameters - tear breakup time (TBUT) (measured in seconds, with lower values indicating less tear stability), fluorescein corneal staining (graded to the National Eye Institute (NEI) scale, with higher values indicating a more irregular epithelium), and tear production Schirmer strips test (measured in millimetres of wetting in 5 min, with lower values indicating less tear production)  2. questionnaire data - Dry Eye Questionnaire 5 (DEQ-5), the Ocular Surface Disease Index (OSDI), Numerical Rating Scale (NRS), Patient Health Questionnaire-9 (PHQ-9) and the Neuropathic Pain Symptom Inventory modified for the Eye (NPSI-Eye). | •At baseline, all subjects reported unpleasantness with light stimulation (average: 70.8 ± 32.0).  •4-,6-weeks after BoNTA, unpleasantness scores ↓ (48.1 ± 33.6), but the change was not significant.  • 50% of subjects had ↓unpleasantness ratings in response to light stimulation compared to baseline ("responders," *n* = 6; 87.5 ± 10.8 vs. 32 ± 32.7, paired *t*-test *t*(5) = −4.51, *p* = 0.006), while 50% had equivalent (*n* = 3) or ↑ (*n* = 3) unpleasantness ("non-responders", n=6; 54.2 ± 38.3 vs. 64.2 ± 28.0, paired *t*-test *t*(5) = 1.78, *p* = 0.136).  •At baseline, several differences were noted between responders and non-responders; responders had ↑ baseline unpleasantness ratings to light, ↑ symptoms of mild depression [PHQ-9 ≥ 5 vs. <5, χ^2^ (1, *n* = 12) =8.57, *p* = 0.003], and ↑ frequent use of antidepressants [χ^2^ (1, *n* = 12) =8.57, *p* = 0.003] and anxiolytics [χ^2^ (1, *n* = 12) = 8.57, *p* = 0.003], compared to non-responders.  •Baseline vs BoNTA, TBUT (6.9±5.0(12) vs 6.6 ± 2.4(11), P=0.82); Staining (1.9±3.9(12) vs 62.3 ± 3.4(11), P=0.75); Schirmer´s (11.3±5.9(12) vs 13.8 ± 10.1(10), P=0.31)  •No significant differences in tear film parameters were found between G1 and G2.  •Group analysis at baseline displayed light-evoked BOLD responses in bilateral primary somatosensory (S1), bilateral secondary somatosensory (S2), bilateral anterior insula, paracingulate gyrus, midcingulate cortex (MCC), bilateral frontal pole, bilateral cerebellar hemispheric lobule VI, vermis, bilateral cerebellar crus I and II, and visual cortices.  •BoNTA injections significantly ↓ light evoked BOLD responses in bilateral S1, S2 cortices, cerebellar hemispheric lobule VI, cerebellar crus I, and left cerebellar crus II. Both activation and contrast maps had an individual voxel threshold of *z* > 2.3, and cluster-threshold of *p* < 0.05.  •G1 displayed activation of the spinal trigeminal nucleus (SpV) at baseline where G2 did not. The activity of SpV during photophobia may predict the success of BoNTA injections as a therapy for individuals suffering from chronic ocular pain.  •BoNTA modulate light-evoked activation of pain-related brain systems and photophobia symptoms in some individuals with chronic ocular pain. These effects are associated with ↓ activation in areas responsible for processing the sensory-discriminative, affective, dimensions, and motor responses to pain. |
|  | LIMITATIONS: BoNTA administered irrespective of concomitant ocular surface treatments or the use of systemic pain medications and mood modulators may have influenced unpleasantness ratings (e.g., G1 more frequently reported mild or greater symptoms of depression and use of anti-depressants and anxiolytics compared to G2). Small cohort with differences in demographics and co-morbidities which may have impacted the findings. The protocol used an event-related model of light stimuli. Therefore, the observed significant BOLD activated regions may be related to acute pain processing in addition to, or rather than, regions associated with chronic ocular pain and photophobia. | | | | | |
| Sebastianelli, 2023 | (n=15) with medication overuse headache. Age: 18-65ys  Follow-up: 1-, 3-months after one session of pericranial BoNTA (PREEMPT protocol) | Chronic migraine  diagnostic criteria of the International Classification of Headache Disorders (ICHD third edition) | | | **cephalic and extracephalic (trigeminal and extratrigeminal) nociceptive and lemniscal sensory systems**  **activity of the caudal trigeminal nucleus** -nociceptive blink reflex (nBR),  **integrity of cervical motoneurones related to the trigeminal system**- trigemino-cervical reflex (nTCR),  **activity of the cingulate area**-pain-related cortical evoked potential (PREP),  **integrity of the non-pain-related somatosensory lemniscal system**- upper limb somatosensory evoked potential (SSEP)  (by electrophysiological techniques)  EMG and cortical activities were carried out using an analysis time window of 500 ms after the delivery of the electrical stimulus.  The following parameters were measured both for nBR and nTCRs: sensory thresholds (detection and pain), latency, area under the curve (AUC), and its habituation. Latency, peak-to-peak amplitude, and habituation were also measured for PREP.  Other assessments: headache diaries-percentage changes in monthly days with headache, mean severity of headache (VAS) and monthly number of acute medication intake. | •3-months after a single session of prophylactic therapy with BoNTA in CM patients: ↑ homolateral and contralateral nBR AUC, an enhancement of the contralateral nBR AUC habituation slope and the nTCR habituation slope, ↓ PREP N-P 1st and 2nd amplitude block, and no effect on SSEPs.  **nBR**: significant repeated time measurements effect for the mean pain threshold after supraorbital stimulation (F = 3.23; *p* < 0.05), which significantly increased at T3 compared to T0 (between-group test *p* = 0.04), while the mean sensory detection threshold did not change significantly (S = 1.02, *p* = 0.599); significant repeated time measurements effect for the AUC of the 1st ipsilateral (S = 6.00, *p* = 0.05) and contralateral nBR block (F = 5.31, *p* = 0.01). Between-group tests revealed that the AUC was significantly increased at T3 compared to T0 for the ipsilateral 1st block (Dunn–Bonferroni test = 5.73, *p* = 0.05) and for all the three contralateral blocks (1st block *p* = 0.010; 2nd block *p* = 0.040; 3rd block *p* = 0.042); significant repeated time measurements effect was found for the habituation slope calculated between the 1st and the 2nd block of the contralateral (S = 6.03, *p* = 0.049), but not of the ipsilateral nBR AUC (S = 1.98, *p* = 0.151). The between-group analysis revealed that the contralateral habituation slope at the 2nd block was significantly more pronounced at T3 than at T0 (*p* = 0.049). The repeated time measurements effect was not significant for the nBR AUC habituation slope calculated between the 1st and the 3rd nBR blocks, both for ipsilateral (S = 1.28, *p* = 0.528) and contralateral (F = 1.19, *p* = 0.316) responses.  **nTCR**: no significant repeated time measurement effect for nTCR onset latency (F = 0.41, *p* = 0.664), duration (F = 1.23, *p* = 0.303), grand-average AUC (S = 2.60, *p* = 0.273), 1st block AUC (S = 2.22, *p* = 0.330), or habituation slope calculated between the 1st and 2nd block (S = 1.38, *p* = 0.5). Only the nTCR habituation slope between the 1st and 3rd block (S = 5.71, *p* = 0.028) showed a repeated time measurements effect. Between-group analysis revealed that the nTCR habituation slope was more pronounced at T3 than at T0 (*p* = 0.024).  **PREP**: significant repeated time measurements effect was observed for PREP N-P 1st and 2nd block amplitude (F = 3.40, *p* = 0.043; F = 4.77, *p* = 0.014, respectively). On the between-group test, the N-P 1st and 2nd block amplitudes were significantly reduced (*p* < 0.05, *p* = 0.025, respectively) at T3 compared to T0; no significant repeated time measurements effect for N (S = 2.85, *p* = 0.240) and P (F = 0.22, *p* = 0.802) latencies, or PREP amplitude habituation at 2nd (F = 2.87, *p* = 0.069) and 3rd (F = 0.30, *p* = 0.746) block of averaging.  **SSEP**: None of the latency and amplitude parameters of the various SSEP components showed a significant repeated time measurements effect, with the same being true for the habituation slope measured at 2nd and 3rd blocks.  •electrophysiological evidence for the ability of a single session of BoNTA injections to exert a neuromodulatory effect at the level of trigeminal system through a ↓ in input from meningeal and other trigeminovascular nociceptors. Moreover, by ↓ activity in cortical pain processing areas, BoNTA restores normal functioning of the descending pain modulation systems.  •BoNTA clinical data: significantly ↓ the number of headache days at T1 (between-group test *p* = 0.014) and T3 (between-group test *p* < 0.001) compared to T0; significantly ↓ acute medication intake at T1 (between-group test *p* = 0.006) and T3 (between-group test *p* < 0.001) compared to T0; Headache severity significantly ↓ 3 months (T3 vs. T0: *p* = 0.001), but not 1 month (T1 vs. T0: *p* = 0.301).  •no statistically significant relationship between the percentage changes in neurophysiological variables at 1 or 3 months and the percentage changes in clinical variables (days with headache/month, mean pain severity and number of acute medications/month).  •Absent correlation between trigeminal (nBR and nTCR) and central (PREP) nociceptive electrophysiological responses and clinical changes suggests that the clinical efficacy of BoNTA is mainly due to peripheral modulation of the trigeminovascular sensory system. |
|  | LIMITATIONS: Funded by Allergan (Pharmaceutical), small sample size and short follow-up (3months), did not use laser stimulation for analyzing the effect of BoNTA on C-fibers, no control-goup? | | | | | |
| Ozarslan, 2022 | (n=44 out of 53) female accepted to participate and met the inclusion criteria.  G1. Chronic migraine (n=22, 21 completed T1, 19 completed T2) F, Mean age: 38.1 ± 7.2ys  Follow-up: 1–7 days (T0-1week) before, 28–35 (T1-1month), and 84–91 days (T2-3months) after treatment.  Single BoNTA injections PREEMPT protocol  G2. (n=22) healthy women/ control group, Mean age: 36.6 ± 7.6ys Follow-up: examined once  Non-randomized clinical study LOE-III | Cutaneous allodynia in Chronic migraine (Classification Committee of the International Headache Society’s (IHS) third edition of the International Classification of Headache Disorders (ICHD-3) | | | **Forehead and hand**  The heat (HDT) and cold (CDT) detection thresholds - bilateral quantitative sensory testing (QST)  Other assessments: Headache characteristics, medication intake, allodynia, presence of anxiety, and depression symptoms (through relevant scales- MIDAS, VAS, BDI, BAI, and ASC-12 scores) | •G1. migraine (average of 22.5 ± 6.1ys), CM for 6.1 ± 3.2ys, painful days/month (average 22.1 ± 4.0 days). All the patients had migraine attacks with CA (mean 5.6/month). The average allodynia symptom checklist (ASC-12) score was 7.8 ± 6.2.  **Baseline**:  •Thermal thresholds in G1 were similar to G2. Thermal thresholds did not show significant differences between the symptomatic and the asymptomatic sides at the last migraine attack.  •There was also no correlation between the allodynia revealed by the physical examination and the thermal thresholds detected by QST.  •G1 was more depressive and anxious than G2, mean BDI in G1 (24.1 ± 11.5) was significantly ↑ than G2 (9.5 ± 6.3) (p < 0.001). Depression symptoms were detected G1 (n=19), and G2 (n=9). mean BAI score G1 (23.3 ± 14.7) was also significantly ↑ than G2 (6.4 ± 8.3) (p < 0.001). symptoms of anxiety G1 (n=17) and G2 (n=4).  **After BoNTA**:.  •The ASC-12 score ↓ significantly with BoNTA (p = 0.030), but no significant change was observed in thermal thresholds after this treatment:  Hand - Heat (T0: -0.096, P=0.537, T1: 0.175, P= 0.281, T2: 0.148, P= 0.374); Cold (T0: -0.032, P= 0.836, T1: -0.027, P= 0.871, T2: 0.176, P= 0.298)  Forehead - Heat (T0: -0.207, P= 0.177, T2: -0.114, P = 0.483, T2: 0.172, P= 0.310); Cold (T0:-0.288, P= 0.058, T1: 0.007, P= 0.964, T2: 0.345, P= 0.037).  •There was no significant correlation between CA and thermal thresholds. BoNTA was successful in relieving headache and other associated symptoms, including CA, but had no significant effect on QST parameters.  • the frequency and severity of the headache and CA ↓ significantly after BoNTA, which supports the view that BoNTA reduces central sensitization. Its ineffectiveness on thermal thresholds supports the opinion that BoNTA has no effect on threshold level stimuli and preferably blocks the effects of suprathreshold stimuli that cause pain. |
| Valente, 2021 | (n=22)  G1. chronic migraine without aura (n=11, 8 completed - 1 discontinued BoNTA, 2 did not accept repeating the study protocol).  G2. episodic migraine without aura (n=11)  9F,2M in both the EM and CM groups. Mean age: 32.4ys (G2), 36.5ys (G1)(*p* = 0.17).  Follow-up: 1year  BoNTA – PREEMPT protocol (minimum dose in an individual patient was 155U, maximum dose-195U) | Chronic migraine without aura | | | **Brain-cortical**  changes in excitability and plasticity  (single- and paired-pulse transcranial magnetic stimulation - TMS)  Other assessments: clinical pain - MIDAS score | •At baseline, compared with G2, G1 had a lower threshold in both hemispheres (right hemisphere: 46% ± 7.8 vs. 52% ± 4.28, *p* = 0.03; left hemisphere: 52% ± 4.28 vs. 53.54% ± 6.58, *p* = 0.02).  •In G2, paired-pulse stimulation elicited a physiologically shaped response; G2, physiological intracortical inhibition (ICI) between 1 and 3 ms intervals was absent at baseline. On the contrary, increasing intracortical facilitation (ICF) was observed for all interstimulus intervals (ISIs).  •In G1, cortical excitability was partially ↓ after BoNTA, along with a significant ↓ observed in MIDAS score (from 20.7 to 9.8; *p* = 0.008).  •The lower motor threshold in G1 compared to G2 reflects a ↑ cortical hyperexcitability. The lack of physiological ICI in CM could indicate sensitisation of the trigeminovascular system. Although reduced, this type of response is still observable after treatment, despite a marked clinical improvement.  •The study suggests a long-term alteration of cortical plasticity due to chronic pain.  •Rest motor threshold (RMT) in CM patients was not modified by BoNTA treatment. BoNTA does not modify the corticospinal pathway excitability. Specifically, BoNTA does not change RMT, CCT, or cortical silent period (CSP) - hypothesised that the mechanism of action of BoNTA in chronic migraine is not mediated by voltage-gated sodium channel binding or by interaction with GABA_B_ receptors.  •At baseline, paired-pulse transcranial magnetic stimulation (pp-TMS) exhibited a significant lack of physiological SICI between 1- and 4-months in CM patients; conversely, in the same interval, a facilitating response occurred. Facilitation was maintained and further amplified for ISIs of 6, 10, and 15 ms. This “paradoxical” facilitation at ISIs of 1–4 ms was partially, although not significantly, ↓ by BoNTA.  •From a clinical point of view, after BoNTA treatment, marked ↓of pain in CM, with the mean MIDAS score decreasing from 20.7 to 9.8 (*p* = 0.008). A post-hoc power analysis revealed low to moderate power (0.20–0.40) for the pp-TMS variations in CM between pre-treatment and post-treatment measurements as well as a moderate to high power (0.71) for single-pulsed (sp)-TMS parameters between G2 and G1. |
| Kim, 2019 | (n=24, 23 completed) 1 excluded for protocol violation. 6M, 17F) mean age (35.2±10.9ys)  Follow-up: 2, 4, 8, 12-weeks after BoNTA (biophysical measurements), each parameter measured 3 times.  Intradermal injection split-face:  G1. BoNTA (15U) treated side  G2. saline  Prospective, single-centre, randomized (computer.generated), double-blind, split face, placebo-controlled trial LOE-II | Rosacea  (mild to moderate erythemato-telangiectatic – type rosacea and facial erythema on the cheeks) – standard guidelines of the National Rosacea Society Expert Committee | | | **Skin**  **Biophysical measurements: skin/stratum corneum hydration, transepidermal water loss (TEWL),**  **melanin content, erythema index, elasticity, and sebum secretions**  (by corneometer, mexameter, reviscometer, sebumeter device)  Other assessments - Clinician Erythema Assessment (CEA) score, Global Aesthetic Improvement Scale (GAIS) score. | • G1, the mean CEA score was significantly ↓ (week 4, 8) (P<0.01), mean GAIS score were significantly ↑ (week 2, 4, 8 ) (P<0.05, <0.01, <0.01).  • Erythema index: was ↓ throughout the 12-weeks in G1, with statistical differences at week-4, 8 (compared to baseline; P<0.01; compared to G2; P<0.05). % change showed differences, significant at week-4 (P<0.05) and week-8 (P<0.01)  • Melanin index did not differ significantly between sides during the 12-weeks.  • Skin elasticity (reviscometer): in G1, significantly ↑ compared to baseline (week-2, P<0.01); week-4, P<0.05); changes in reviscometer were significantly greater than G2 at week-2,-4, P<0.01. % change was statistically significant as compared to G2, P<0.05, P<0.01.  • Skin hydration levels (corneometer): in G1 was improved throughout the 12-weeks, with significant improvements compared to baseline at week-2, 4, 8 (P<0.01). Compared to G2, at week-2 (P<0.05). changes in corneometer values were greater in G1 compared to G2 at week-2 (P<0.05), week-4, 8 (P<0.01). % change was ↑ on G1 at week-2 (P<0.05), week-4, 8 (P<0.01).  • TEWL: did not change significantly during the 12-weeks, not significantly change between G1 and G2 (P>0.05).  • Sebum secretion: did not change significantly during the 12-weeks, no statistically significant changes between G1 and G2 (P>0.05).  • BoNTA is a favourable treatment for facial erythema and rejuvenates the skin with rosacea. |
| Hubbard, 2016 | (n=24, 23 completed) 18F, 6M; Age:18–59ys, mean=38.92 ± 12.75)  BoNTA (150U) injected intramuscularly to 13 different facial and pericranial sites.  At least 2 cycles of BoNTA injections, some patients had 3 treatments, occurring at 3-month intervals.  Follow-up: 4 weeks pre-treatment, 2 weeks posttreatment (interictal)  G1. (n=11;8F,3M) Responders (50% reduction in migraine frequency and <15 headache days/month)  G2. (n=12;9F,3M) Non-responders (frequency remained unchanged)  Retrospective clinical study. LOE-IV | Chronic migraine  (International Classification of Headache Disorders-II) | | | **Brain**  cortical thickness  (using surface-based morphometry)  Other assessments: areas showing group differences in cortical thickness displayed altered resting-state functional connectivity (RS-FC) - seed-to-voxel analyses | •G1 showed significant cortical thickening in the right primary somatosensory cortex (SI) and anterior insula (aINS), and left superior temporal gyrus (STG) and pars opercularis (ParsOp) compared to G2.  •Disease duration was negatively correlated with cortical thickness in fronto-parietal and temporo-occipital regions in G1 but not G2, except for primary motor cortex (MI) that showed the opposite pattern.  •Disease duration was positively associated with MI cortical thickness in G1 versus G2. Seed-based RS-FC analyses revealed anti-correlations between the SI seed and lateral occipital (LOC) and dorsomedial prefrontal cortices (DMPFC) in G1, whereas non-responders showed ↑ connectivity between the ParsOp seed and LOC.  •Overall, findings revealed distinct morphometric and functional brain changes in CM patients that reverted to episodic migraine (EM) following prophylactic treatment compared to CM patients that showed no change in disease status. |
|  | LIMITATIONS: small sample size, retrospective study design and the lack of headache diaries documenting the treatment effect, given that patient retrospective self-reports are unreliable and may be prone to memory related biases. It is unclear the extent to which treatment responsiveness was due to placebo or prophylactic treatment effects, or the premorbid brain state (i.e., no baseline cortex measurements) to clarify whether drug or placebo and its contributions to the observed morphological and functional brain changes and whether these changes represent reliable markers of migraine chronification and reversal. | | | | | |
| de Tommaso, 2016 | (n=20)  Follow-up: T0-baseline, T1-7 days, T2- 1 year (clinical outcome)  G1. BoNTA single administration in pericranial muscles - PREEMPT protocol  G2. Saline  Double blind placebo controlled crossover design LOE-II | Refractory Chronic Migraine | | | **Skin – right frontal and trapezius injection sites and hand dorsum**  Neurophysiological method - CO2 Laser Evoked Potentials (LEPs) obtained by the stimulation of the skin  N1, N2 and P2 amplitude and latencies and N2P2 habituation index were evaluated and correlated with the percent change of headache frequency | •The LEPs performed at T0 and T1 before G1 or G2 were almost super-imposable, and statistical analysis of Student’s t-test did not report relevant differences. The N1, N2 and P2 amplitudes and latencies in basal conditions were within normal limits for all the stimulation sites.  •T0, subjective pain induced by laser stimuli was significantly ↑ in CM patients compared to healthy controls at trigeminal level (controls trigeminal Visual Analogic Scale (VAS): 41.32 ± 12.2; CM patients 54.78 ± 21.84: ANOVA F 4.90 p = 0.02).  •T1, G1 caused a slight and not significant laser pain perception ↓ by the hand, trapezium and frontal levels, and also G2 exerted no relevant effect.  •T0, latencies were within normal limits. In T1 - G1 had significant prolongation of N2 and P2 latencies at the hand level, in respect to T0 and T1 – G2, with a similar though not significant trend for N1 component. When the recordings from the skin over trapezius and frontal muscles were considered, no significant difference was found among the different conditions.  •T0, LEPs amplitudes were not different from the G2. Moreover, the N1 and N2P2 amplitudes were similar between T0, T1 - G2 and T1 - G1.  •T0, the vertex complex showed ↓ habituation in respect to control group when the face and the hand were considered, while a not significant trend was observed in the skin over the trapezius stimulation (in the controls, habituation index for face stimulation was 48.8% ± 22.2% ANOVA F 5.71, p = 0.011; hand stimulation 49.9 ± 27.7 F 4.68, p = 0.023; and trapezius 41.1 ± 21.2 F 3.19, p = 0.065). At trigeminal level, habituation index was significantly ↑ in T1 - G1, while at the hand and shoulder levels, there was a not significant trend toward a habituation index ↑ in G1. In CM patients, at trigeminal level, G1 restored habituation within normal limits. In addition, the effect of BontA of trigeminal habituation prevailed in patients with more evident LEPs abnormalities, as there was a significant correlation between the habituation index at T0 and percent degree of habituation changes induced by BontA in T1 condition.  •T1. In G1 a normalization of the trigeminal habituation index was observed, which was correlated with the clinical outcome in T2.  •Patients displaying trigeminal LEPs facilitation at T0 showed a more efficient therapeutic outcome. •Neurotoxin may exert a modulating effect on trigeminal nociception and altered pattern of habituation, normalizing central neurotransmission. |
|  | LIMITATIONS: The small case series reduced the reliability of results, poor collaboration for the neurophysiological procedures. The low compliance in clinical diaries also caused the exclusion of many patients. The placebo-controlled study design caused half patients to be recorded after three months from the first toxin cycle, though neurophysiological pattern was similar to the basal condition for the long time elapsing from the injection. Patients were under symptomatic and preventive therapies, giving that the study was observational under the clinical point of view, so we were not allowed to discontinue treatments. Finally, the seven days after effects of BontA on LEPs could not completely explain the long-term efficacy mechanism in chronic migraine. | | | | | |
| Borodic, 2014 | (n=1) 1F 62ys  Biopsy showed inflammation – muscle and skin (diagnosis of linear scleroderma)  Follow up: 12-, 22-ys  BoNTA injections into forehead and scalp (total 60 50U divided over 6 locations in the afflicted side) | Parry-Romberg syndrome vasculopathy with chronic pain – right brow, forehead, and scalp – and atrophy brow, forehead - and progressive hair loss | | | **Face, Orbit, Brain**  Atrophy – MRI  Blood flow – MRI spectroscopy and technetium-99m single-photon emission computed tomography scan | •Baseline. Pain that was intermittent become constant, rated 9 out of 10 in severity and causing inability to maintain gainful job. After BoNTA, there was dramatic improvement of the pain.  •After 12 years, MRI (face and orbit) – severe right facial atrophy involving eyelid, orbit, muscle of mastication and progression of right enophthalmos; MRI (brain) – bilateral sulcal prominence suggestive of cerebral gyral atrophy associated to blood flow abnormality of the affected side.  •10 years later, further injections were instituted and resulted again in dramatic relief of pain, hair loss, and improvement in memory. |
| Bumb, 2013* | (n=111)  BoNTA – PREEMPT protocol (smaller dose - 100IU vs 155IU)  G1. (n=47) Responders (patients who underwent 3 or more treatments)  G2. (n=64) Non-responders (1 or 2 treatments)  Retrospective observational study – LOE IV | Chronic migraine | | | **Axial T2 and coronary FLAIR (fluid attenuated inversion recovery) sequences**- white matter lesions (WML) small (3–5 mm), medium (6–9 mm) or large (>10 mm)  (by MRI) | •Response rate to BoNTA in migraineurs with WML was 55.3%, in migraineurs without WML 44.7%.  •In the investigated items “age”, “age at onset”, “gender”, “attack duration”, “frequency”, “aura”, “WML”, “size of WML”, there was no statistically significant difference between the two groups.  •55% in G1, 50% in G2 showed WML. All WML were located supratentorially, anteriorly, mostly of small size (3–5 mm).  •WML on MRIs cannot serve as a marker to predict a positive response to BoNTA. |
|  | LIMITATIONS: retrospective study design, small sample size and quantity of available MRIs might be too small. Definition of responders and non-responders may contribute to some false results. * marker collected only before BoNT administration | | | | | |
| Lee, 2016* | (n=83, 73 completed, 3 excluded with vascular abnormalities)  Follow-up: 4-6 weeks  BoNTA PREEMPT protocol  Responders (n=42, 60.0%) - (1) ≥50% reduction of headache days, (2) ≥50% reduction of acute abortive medication intake frequencies, or (3) ≥50% reduction of headaches with moderate-to-severe intensities  Clinic-based prospective study LOE III | Chronic migraine (international classification of headache disorders, 3^rd^ edition beta version (ICHD-3 beta) | | | **Brain**  **Cerebral blood flow** (by interstitial Transcranial Doppler testing (TCD) of bilateral middle cerebral arteries (MCAs), posterior cerebral arteries (PCAs), basilar artery (BA), and extracranial ICAs (ICAs).  Mean flow velocities (MFVs) of each vessel.  MCA/ICA index - ratio of MCA MFV over the ipsilateral ICA MFV on each side.  Korean version of the Headache Impact Test-6 (HIT-6) | • Mean monthly headache days: baseline (25.9 ± 6.0), post-treatment (17.0 ± 11.01). Mean differences of monthly headache days were 8.9 ± 10.7.  • Responders (n=42, 60.0%):: 50% reduction of headache days (n = 31, 44.2%), 50% reduction of acute abortive medications (n = 26), and 50% reduction of headaches with moderate-to severe intensities (n = 24)  • Longer disease duration was associated with poor treatment outcome (p = 0.019).  • The ratio of the mean velocity of middle cerebral artery to that of ipsilateral internal carotid artery (MCA/ICA index) was significantly ↑ in responders than non-responders (p = 0.027) and remained significant after controlling for covariates (p = 0.025).  • Neurosonographic predictors of BoNTA treatment response: shorter disease duration and higher pretreatment MCA/ICA index on TCD are associated with good early outcomes of BoNTA treatment. |
|  | LIMITATION: * marker collected only before administration | | | | | |
| **NO RESULTS** | | | | | | |
| Ernberg, 2023  ClinicalTrials.gov Identifier: NCT05720065 | (n=40) 20-45 Years, Adult female  Follow-up:1,6-months  G1. BoNTA single bilateral injection into 3 standardized points of masseter, 2 of anterior temporalis (100U,10U/point- 0.1ml solution/point).  G2. Single injection of 1 mL isotonic saline (0.9mg/mL)  Phase 2, Randomized, Controlled, Double-blind Study – LOE- II | Myogenous temporomandibular disorders | | | **Gene expression** - bulk RNA-seq  **Epigenetic signature** – ATAC  **Sensory neuron markers** measured with IHC  Other assessments: pain intensity (Brief Pain Inventory), global improvement (Patient Global Impression of Change Scale (PGIC), pain quality (McGill Pain questionnaire (MPQ), adverse events. | •No results yet  Other measurements: physical and emotional function (Axis II questionnaire,DC/TMD, Brief Pain Inventory, Jaw Function Limitation Scale, Oral Behavior Checklist, Beck's Depression inventory, Generalized Anxiety Disorder, Patient Health Questionnaire, Pain Catastrophizing Scale, Perceived Stress Scale, Insomnia Severity Index), pressure pain threshold (PPT), conditioned pain modulation (CPM), temporal summation pain. |
| Austen, 2017  ClinicalTrials.gov Identifier: NCT03381261 | (n=49) Age: 18-65ys  Follow-up: 1 year  BoNTA injection on one side of the back of the head.  Open label, single group assignment LOE-III | Chronic Migraine Classification of Headache Disorders (ICHD-III) criteria | | | **Discarded tissue of migraine surgery** -  **Molecular markers.**  Gene Expression Code Set profiling 594 genes; 579 immunology-related human genes + 15 internal reference controls. | •Completed without published results |
| Hordinsky, 2016  ClinicalTrials.gov Identifier: NCT00816517 | (n=8) Age: 18-65 Years  BoNTA (35-100U) injected around a skin lesion (plaque) one time.  Follow-up: baseline, 3-months  Single Group Assignment Pilot clinical trial LOE-III | Psoriasis Vulgaris  (at least 1 area that has been intolerant/ recalcitrant to recognized topical/ systemic treatments, at least a score of 2 for keratoderma and erythema) | | | **Skin**  3mm skin biopsy  Other assessments: psoriasis scoring scale | •Completed without published results |
| Zelickson 2022  ClinicalTrials.gov Identifier: NCT05456087 | (n=20) 10F, 10M Age: 22-45 years  One time session, BoNTA 30 injection sites evenly distributed within the hair loss area (5U/site, max-150U)  Follow-up: Day 30, 90, 180, and 270  Open label, Single Group Assignment Clinical trial LOE-III | Androgenic Alopecia | | | **Scalp**  Other assessments: Hair density (count/cm2), hair shaft diameter, thickness, hair count follicular units – Trichoscopy imaging (Canfield HairMetrix® system) | Recruiting, no results yet |
