# Supplementary material for: Botulinum toxin effects on biochemical biomarkers related to inflammation-associated head and neck chronic conditions: a systematic review of clinical research
Source: J Neural Transm (Vienna). 2025 Mar 4;132(12):1851–74. doi: 10.1007/s00702-024-02869-w (PMC12669376; doi:10.1007/s00702-024-02869-w)
Supplement: Supplementary file 8 — Supplementary file8 (DOCX 68 KB) [file 702_2024_2869_MOESM8_ESM.docx]

**Supplementary Information 5:** Biomarkers in Clinical Research on Botulinum Toxin effects on Chronic Inflammatory State. GRADE consistency assessment. RoB Report. Reporting Bias.

| **Biomarker**  **(CIS)**  **Biological**  **sampling** | | **Author**  **Year** | **Unit measure** | **BoNT Key effect (Outcome)**  **Summary** | **Justification / Criteria (*)** | | | | **Overall**  **GRADE** |
| --- | --- | --- | --- | --- | --- | --- | --- | --- | --- |
|  |  |  |  |  | **1. Risk of bias**  **2. Imprecision**  **3. Inconsistency**  **4. Indirectness**  **5. Publication bias** | **Downgrade**  **(RCT)** | **Upgrade**  **(nRT)** | **1.** **Large effect**  **2. Dose-response gradient**  **3. Opposing plausible residual confounding and bias** |  |
| **NEUROPEPTIDE** | | | | | | | | | |
| **CGRP** | **Plasma** | | | | | | | | |
|  | **CM** | **Cernuda-Morollón, 2015** | (pg/mL) | 2 nRT (n=128 BoNT; n = 30 control)  RoB: low - moderate  B-T1 (1 month) - (Mean (range) 34.30±14.99 (18–52 days): (--) | Start: Low quality (nRCT) due to well-designed studies with low to moderate risk of bias. | | | | 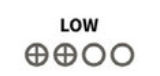 |
|  | **TN** | **Zhang, 2020** |  |  |  |  |  |  |  |
|  | **Saliva** | | | | | | | | |
|  | **CM** | **Cady, 2014**  **NCT01071096** | (pmol/mg total protein) | 1RCT (n=9 BoNT; n=10 control)  RoB - High  B-T1 (1^st^ month): NS  B-T2 (3^rd^ month): (-) | Start: High quality (RCT)  Downgraded: Moderate quality - (1) High Risk of bias.  Downgraded: Low quality - (2) Imprecision - estimate comes from only one small study.  (5) One author received grant from a pharmaceutical. It was not judged as problematic to objective outcome. | | | | 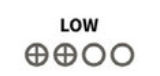 |
|  | **Skin** | | | | | | | | |
|  | **CM** | **Cady, 2016**  **NCT02037425** | Biomarker change (0–3, scale) | 1 nRT (n=32, 14 completed endpoint)  RoB - Serious  B-T1 (12 weeks): NR  NS | Start: Very low quality (nRCT) with high risk of bias | | | | 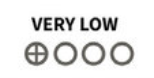 |
|  | **Scalp skin (SNP, Super-Fol, BULGE)** | | | | | | | | |
|  | **CA** | **Cutrer, 2010** | Average number of immunoreactive fibres.  Scale bar =100 mm | 1nRT (n=1)  RoB - Moderate  B-T1 (34 days): CGRP (SNP): NS  CGRP (Super-Fol): (+)  CGRP (BULGE): considerable (+) | Start: Low quality (nRCT) due to well-design study with moderate risk of bias. | | | | 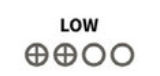 |
| **SP** | **CA** | **Cutrer, 2010** | Average number of immunoreactive fibres.  Scale bar =100 mm | 1nRT (n=1)  RoB - Moderate  B-T1 (34 days):  SP (SNP): considerable (+)  SP (Super-Fol): (+)  SP (BULGE): considerable (+) | Start: Low quality (nRCT) due to well-design study with moderate risk of bias | | | | 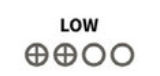 |
| **MONOAMINE NEUROTRANSMITTER / 5 - HYDROXYTRYPTAMINE** | | | | | | | | | |
| **Serotonin** | **Tear** | | | | | | | | |
|  | **DED** | **Choi, 2019** | detected ≥ {} of 0.30 ng/mL | 1 RCT (n=13/26 eyes BoNT; n=13/26 eyes control)  RoB - Low  B-T1 (1 month): (--) | Start: High quality (RCT)  Downgraded: Moderate quality - (2) Imprecision - estimate comes from only one small study.  (5) One author received grant from a pharmaceutical. It was not judged as problematic to objective outcome. | | | | 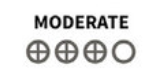 |
|  | **Serum and peripheral blood platelet** | | | | | | | | |
|  | **CD(p)-A/D** | **Karakulova, 2017** | {}  (ng/mL) | 1nRT (n=48 BoNT; n=15 control)  RoB - Moderate  B-T1 (1-month):  Serum: (-)  Peripheral blood platelet: (++) | Start: Low quality (nRCT) due to well-design study with moderate risk of bias | | | | 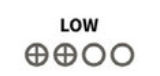 |
| **PLAYERS IN INFLAMMATORY PROCESSES** | | | | | | | | | |
|  | **Scalp skin (SNP, Super-Fol, BULGE)** | | | | | | | | |
| **Mast cells degranulation** | **CA** | **Cutrer, 2010** | Biomarker change scale 0-4 score | 1nRT (n=1)  RoB - Moderate  B-T1 (34 days):  SP (SNP): NS  SP (Super-Fol): NS  SP (BULGE): (↓) | Start: Low quality (nRCT) due to well-design study with moderate risk of bias | | | | 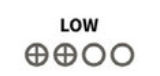 |
| **Expression of inflammatory genes -** **cytokine signaling, lymphocyte activation, innate immune response, TNF family signaling**, **TLR signaling**, **altered genes** | **Muscle, fascia, periosteum - Tissues from neck and occiput (occipitalis, spenius capitis, semispinalis capitis, trapezius) - RNA** | | | | | | | | |
|  | **CBOH-MF** | **Gfrerer, 2022** | 579 pro-inflammatory genes (mean number, 95% CI)  * data prepared (site with highest effect reported) | 1nRT(n=18, 108 samples)  RoB - Low  B-T1 (1-month):  Periosteum responders (--)  Periosteum non-responders (+) | Start: Low quality (nRCT)  Upgraded: Moderate Quality – large effect in the periosteum, based on a well-done observational study without important risk of bias or other limitations | | | | 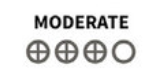 |
| **OXIDATIVE STRESS BIOMARKERS** | | | | | | | | | |
| **AOPP** | **Plasma** | | | | | | | | |
|  | **CM** | **Dini, 2019** | nmol/mL | 1nRT (n=27 BoNT; n=27 control)  RoB - Moderate  B-T1 (6-months): (--) | Start: Low quality (nRCT) due to well-design study with moderate risk of bias | | | | 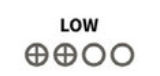 |
| **FRAP** | **CM** | **Dini, 2019** | nmol/mL | 1nRT (n=27 BoNT; n=27 control)  RoB - Moderate  B-T1 (6-months): (++) | Start: Low quality (nRCT) due to well-design study with moderate risk of bias | | | | 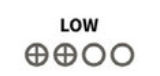 |
| **SH** | **CM** | **Dini, 2019** | µmol/mL | 1nRT (n=27 BoNT; n=27 control)  RoB - Moderate  B-T1 (6-months): (++) | Start: Low quality (nRCT) due to well-design study with moderate risk of bias | | | | 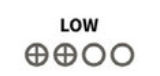 |
| **IMMUNE CELL CLASSES** | | | | | | | | | |
| **Cytokines** - C5/C5a, CD40 Ligand, G-CSF, GRO-α, sICAM-1, IFN-y, IL-1α, IL-1β, IL-1ra, IL-8, IL-16, IL-17E, & IL-23, IP-10, I-TAC, MIF, Serpin E1, and RANTES | **Saliva** | | | | | | | | |
|  | **CM** | **Cady, 2014**  **NCT01071096** | Florescent Unit-Fold change  Mean (SD)  * data prepared (reported subclass of cytokine with min and max value) | 1RCT (n=9 BoNT; n=10 control)  RoB - High  B/interictal (a) – onset pain (b) (T1-T3-month)  NR | Start: High quality (RCT)  Downgraded: Moderate quality - (1) High Risk of bias.  Downgraded two levels: Very low quality - (2) Imprecision - estimate comes from only one small study, unusable samples and missing samples.  (5) One author received grant from a pharmaceutical. It was not judged as problematic to objective outcome. | | | | 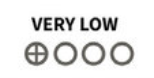 |
| **T-cells: CD8, Th1** (CD4+ T-cells),  **NK cells,**  **B-cells, neutrophils, macrophages, dendritic cells** | **Muscle, fascia, periosteum - Tissues from neck and occiput (occipitalis, spenius capitis, semispinalis capitis, trapezius) - RNA** | | | | | | | | |
|  | **CBOH-MF** | **Gfrerer, 2022** | immune cell type abundance scores | 1nRT(n=18, 108 samples)  RoB - Low  B-T1 (1-month):  Periosteum responders (--)  Periosteum non-responders (+) | Start: Low quality (nRCT)  Upgraded: Moderate Quality – large effect in the periosteum, based on a well-done observational study without important risk of bias or other limitations | | | | 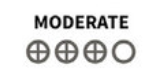 |
| **MMP-9** | **Tear** | | | | | | | | |
|  | **DED** | **Choi, 2019** | detected levels ≥ 40 ng/mL, n (%) | 1 RCT (n=13/26 eyes BoNT; n=13/26 eyes control)  RoB - Low  B-T1 (1 month): (--) | Start: High quality (RCT)  Downgraded: Moderate quality - (2) Imprecision - estimate comes from only one small study.  (5) One author received grant from a pharmaceutical. It was not judged as problematic to objective outcome. | | | | 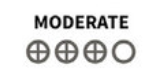 |
| **NEURONAL REGROWTH** | | | | | | | | | |
|  | **Skin** | | | | | | | | |
| **Beta Tublin** | **CM** | **Cady, 2016**  **NCT02037425** | Biomarker change (0–3, scale) | 1 nRT (n=32, 14 completed endpoint)  RoB - Serious  B-T1 (12 weeks): NR  NS | Start: Very low quality (nRCT) | | | | 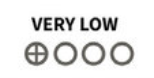 |
| **SNAP-25** | **CM** | **Cady, 2016**  **NCT02037425** | Biomarker change (0–3, scale) | 1 nRT (n=32, 14 completed endpoint)  RoB - Serious  B-T1 (12 weeks): NR  NS | Start: Very low quality (nRCT) | | | | 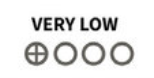 |
| **NERVE FIBRES** | | | | | | | | | |
| **ENF density** | **Scalp skin (SNP, Super-Fol, BULGE)** | | | | | | | | |
|  | **CA** | **Cutrer, 2010** | ENF/mm² | 1nRT (n=1)  RoB - Moderate  B-T1 (34 days): ↑ | Start: Low quality (nRCT) due to well-design study with moderate risk of bias | | | | 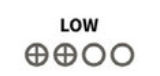 |
| **Nerve fibres morphology** | **CA** | **Cutrer, 2010** | Thickness | 1nRT (n=1)  RoB - Moderate  B-T1 (34 days): ↑ thickness | Start: Low quality (nRCT) due to well-design study with moderate risk of bias | | | | 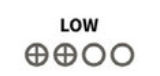 |
| **LEGEND: BoNT,** botulinum toxin; **↑,** higher; **↓,** lower; **↑↑,** significantly higher; **↓↓,** significantly lower; **(++),** increased significantly; **(+)** increased but not significantly; **(--),** decreased significantly; **(-);** decreased but not significantly; **{}**, concentration; **x,** not observed;  **i),** reporting ≤ 10 weeks of benefit; **ii),** reporting no or minimal benefit (≤30%, 3weeks); **NS,** not statistically significant; **RCT,** randomised controlled trial; **nRT,** non-randomised clinical trial; **CM,** chronic migraine; **TN,** trigeminal neuralgia; **CA;** cephalgia alopecia; **CBOH-MF,** Chronic bilateral occipital headache with migraine features; **DED,** dry eye disease; **CD(p)-A/D,** Cervical dystonia with pain in the neck and anxiety/depression; **NR,** not reported; **IT,** injected tissues; **NIT,** non-injected tissues; **log2FC;** log2 fold change; **CI,** confidence interval; **[IQR],** interquartile range; **SE,** standard error; **SD,** standard deviation; **Tx,** treatment; **AOPP,** advanced oxidation protein products; **FRAP,** ferric reducing antioxidant power; **SH,** thiol groups; **G-CSF,** Granulocyte Colony Stimulating Factor; **GRO-α,** Growth Regulated Oncogene alpha; **sICAM-1,** Soluble Intercellular Adhesion Molecule; **IFN-y,** Interferon gamma; **IP-10,** Interferon Gamma-Induced Protein 10; **I-TAC,** Interferon-inducible T cell-α chemoattractant; **MIF,** Macrophage Migration Inhibitory Factor; **RANTES,** Regulated Upon Activation Normal T-cell Expressed; **CGRP,** calcitonin gene-related peptide; **SP,** substance-P; **IL,** interleukin; **TNF-α,** tumor necrosis factor alfa; **TGF-β1,** transforming growth factor β1; **ENF,** epidermal nerve fibre; **SNP,** subepidermal neural plexus; **Super-Fol,** superficial dermis surrounding a hair follicle; **BULGE,** bulge area of hair follicles; **PA,** pain and alopecia; **NPA,** no pain or alopecia; **PPA,** previous pain and alopecia; **MMP,** matrix metalloproteinase.  **KEY:**  **(*)** [based on “What is GRADE? - BMJ Best Practice”. Exception: the level of certainty of non-randomised trials (nRT) were increased one levels (from very low to low) when well-designed studies with low to moderate risk of bias.](https://www.google.com/url?sa=i&url=https%3A%2F%2Fbestpractice.bmj.com%2Finfo%2Fus%2Ftoolkit%2Flearn-ebm%2Fwhat-is-grade%2F&psig=AOvVaw01k7ld5vbphuVgpjOMc9tW&ust=1714676641920000&source=images&cd=vfe&opi=89978449&ved=0CAcQrpoMahcKEwiQo47lku2FAxUAAAAAHQAAAAAQBA)  **GRADE certainty ratings**  **Very low** The true effect is probably markedly different from the estimated effect  **Low** The true effect might be markedly different from the estimated effect  **Moderate** The authors believe that the true effect is probably close to the estimated effect  **High** The authors have a lot of confidence that the true effect is similar to the estimated effect | | | | | | | | | |

**RoB**

**CGRP**

One non-randomised clinical trial and one crossover RCT evaluating CGRP in chronic migraine were judged at low and high risk of bias, respectively (24, 29, 32). Another non-randomised clinical trial was judged at serious risk of bias, given the lack of information on participants baseline characteristics (e.g., potential comorbidities and medications used approved by the investigator) and the collection of data at different time points (not at the time of intervention), being unclear if it was possible to avoid differential misclassification (30). There were also some concerns for the remaining studies in different clinical settings (23, 26). When evaluating CGRP level in trigeminal neuralgia scenarios, two patients in the intervention group discontinued the study due to refusal to drawn blood (23). The control group presented less participants than the intervention group and limited information on sex and age variables, although it was reported that these variables did not influence the outcomes. Data collection was performed at different time points according to treatment response classification (responders, immediately after response confirmation; non-responders, at the end of 2 months), which cannot exclude the risk of introducing systematic errors in measuring the outcome. There was also lack of information on the reported differences in intervention protocol regarding BoNT dose and it is important to bear in mind that BoNT has not been approved by the FDA for the treatment of trigeminal neuralgia. Moreover, the BoNT formulation used in this study has only been approved in China, Brazil, and Russia (23). As for the CGRP in the context of cephalgia alopecia (26), the moderate risk of bias was only ascribed to the potential bias in measurement of outcomes, i.e., it was unclear if the risk surrounding classification of the biomarker change on a scale could be considered negligible.

**SEROTONIN**

One individually parallel-group RCT and one non-randomised controlled clinical trial reported on serotonin levels. The first was judged at low risk of bias, whereas the latter was considered at moderate risk owning to the potentially differences in intervention protocol (dose and site), demographic discrepancies between intervention and control groups, that also presented an unbalanced number of participants, which were not taken into account. It was also unclear if the patients were naïve to BoNT and if medications for comorbidities or preventives were continued throughout the study, variables that could have had a role on the biomarkers change levels reported.

**OXIDATIVE STRESS BIOMARKERS**

One non-randomised study included 27 chronic migraineurs reporting medication overuse and compared to 27 healthy participants. The study was judged at moderate risk due to potential bias in measurement of outcomes, i.e., inappropriate assessment of the weight of continued medication on the biomarker’s levels after treatment. Moreover, the 27 healthy participants that served as control, although matched for age and sex, had significant different biomarker values at baseline.

**PLAYERS IN INFLAMMATORY PROCESSES AND IMMUNE CELL CLASSES**

Two exploratory “within subject” follow-up studies (n = 19), and the two RCTs (n = 45) presented discrepant judgement of risk of bias. The only study evaluated at high risk of bias was the protocol with the RCT crossover design (29), reporting laboratory errors related to processing of samples for cytokine levels in saliva, and hence missing data and re-processing of samples requirements.

**SUBSTANCE P**

Potential bias in measurement of outcomes resulted in the overall score of moderate risk of bias.

**NERVE FIBRES**

The single case experimental study was judged at moderate risk of bias.

**NEURONAL REGROWTH**

The non-randomised trial was judged at moderate risk of bias for domain 1 (covering confounding and focusing on issues before the interventions start) and domain 3 (relating to the intervention itself). Domains concerning to issues after the interventions start, such as domain 5 (missing data, given that only a subset of subjects (14/30) completed the endpoint of interest – neuronal regrowth) and domain 6 (measurement of outcomes) were considered at serious risk of bias.

**Reporting bias**

Interictal (baseline) saliva levels of CGRP and changes between responders and non-responders on interictal levels of cytokines namely, C5/C5a, CD40 Ligand, G-CSF, GRO-α, sICAM-1, interferon gamma (IFN-y), interleukin (IL)-1α, 1β, 1ra, 8, 16, 17E, & 23, interferon gamma-induced protein 10 (IP-10), I-TAC, MIF, serpin E1, and regulated upon activation normal T-cell expressed (RANTES), were assessed in Cady 2014 trial, NCT01071096 (29). However, results for cytokines changes were not reported in the authors´ published report of this RCT (32). The authors concluded that the number of participants analysed was low due to some unusable samples and missing samples making comparisons impossible; only five responders and five non-responders provided enough samples at all time points for comparison. Nevertheless, in the published report based on a small number of 19 participants (one female from the intervention group was lost to follow-up) the authors showed a reduction in baseline/interictal saliva CGRP levels after BoNT administration, from 39.64 ± 7.5 pg CGRP/mg total protein after the first month to 25.5 ± 4.1 pg after the third month (32). This difference did not reach significance nor was it significant when compared to the placebo control, although it narrowly missed statistical significance which can be related to the small sample size. We considered this to be low quality evidence due to imprecision and reporting bias. There were more inconsistencies between the study published report and the registered protocol. For example, different cut off values have been provided to classify subjects-responders to BoNT (≥50% versus >30% in number of headache-days/month from their initial baseline levels). In 2016, the same author conducted a non-randomised trial (NCT02037425) and reported mean changes in CGRP, beta Tubulin, and SNAP-25 after BoNT therapy in chronic migraine (30). However, we could not find a published record providing a comprehensive discussion of the outcomes apart from the clinical trial register with published results. We have unsuccessfully tried to contact the corresponding author for additional information. (CASP critical appraisal checklists with the assessment of missing or incomplete data for each study are available upon request).
